# Combinatorial Assembly of Small Molecules into Bivalent Antagonists of TrkC or TrkA Receptors

**DOI:** 10.1371/journal.pone.0089617

**Published:** 2014-03-06

**Authors:** Fouad Brahimi, Eunhwa Ko, Andrey Malakhov, Kevin Burgess, H. Uri Saragovi

**Affiliations:** 1 Lady Davis Institute-Jewish General Hospital, Montreal, Quebec, Canada; 2 Department of Chemistry, Texas A&M University. Texas, United States of America; 3 Department of Pharmacology and Therapeutics, McGill University, Montreal, Quebec, Canada; 4 Department of Oncology and the Cancer Center, McGill University, Montreal, Quebec, Canada; University of São Paulo, Brazil

## Abstract

A library of peptidomimetics was assembled combinatorially into dimers on a triazine-based core. The pharmacophore corresponds to β-turns of the neurotrophin polypeptides neurotrophin-3 (NT-3), nerve growth factor (NGF), or brain-derived neurotrophic factor (BDNF). These are the natural ligands for TrkC, TrkA, and TrkB receptors, respectively. The linker length and the side-chain orientation of each monomer within the bivalent mimics were systematically altered, and the impact of these changes on the function of each ligand was evaluated. While the monovalent peptidomimetics had no detectable binding or bioactivity, four bivalent peptidomimetics (**2c**, **2d**, **2e**, **3f**) are selective TrkC ligands with antagonistic activity, and two bivalent peptidomimetics (**1a**, **1b**) are TrkC and TrkA ligands with antagonistic activity. All these bivalent compounds block ligand-dependent receptor activation and cell survival, without affecting neuritogenic differentiation. This work adds to our understanding of how the neurotrophins function through Trk receptors, and demonstrates that peptidomimetics can be designed to selectively disturb specific biological signals, and may be used as pharmacological probes or as therapeutic leads. The concept of altering side-chain, linker length, and sequence orientation of a subunit within a pharmacophore provides an easy modular approach to generate larger libraries with diversified bioactivity.

## Introduction

Neurotrophins are dimeric polypeptide growth factors that regulate the peripheral and central nervous systems and other tissues. Neurotrophins (Nerve Growth Factor (NGF), Brain-Derived Neurotrophic Factor (BDNF), and Neurotrophin-3 (NT-3)), as well as their cell surface receptors (p75, TrkA, TrkB, and TrkC) are validated targets for therapeutics in a variety of pathologies ranging from cancer to neurodegeneration [1]–[4].

Neurotrophic activities arise from selective ligand binding to the Trk family of receptors. For example, NGF docks with TrkA, BDNF binds preferentially to TrkB [5], whereas NT-3 interacts preferentially with TrkC but can also bind to TrkA [6]. Trk receptors are typical receptor tyrosine kinases (RTKs), with an ectodomain, a single transmembrane region, and an intracellular tyrosine kinase catalytic domain. The extracellular domain of Trk binds the ligand, leading to activation of the tyrosine kinase, phosphorylation (pTyr) of the Trk intracellular domain, and signal transduction cascades involving kinases mitogen-activated protein kinase (MAPK) and AK mouse thymoma (Akt) [7] that are activated by phosphorylation. Activated MAPK and Akt regulate whether a cell enters a growth, or a survival, or a differentiation pathway [7], [8].

In addition, all the neurotrophins bind to the p75 receptor, a member of the tumor necrosis factor (TNF) receptor superfamily [9]. The p75 receptor has multiple functions depending on the cells in which it is expressed, whether a ligand engages it, and many other variables [10], [11]. We sought to develop Trk-selective binding ligands that exclude p75 signals, and to use these agents to study receptor biology.

Previously mimicry of neurotrophin β-turns was used to develop β-turn cyclic peptides [12]–[15] and β-turn cyclic peptidomimetics [16], [17] of neurotrophins. Recently, the cyclic peptidomimetics [18] and minimalist mimics [19]–[21] were assembled into bivalent mimics based on the hypothesis that this would modify the activity of the compounds, because the target Trk receptors are tyrosine kinases that act as dimers.

Here, we further explore the concept of valency, by altering the linker length and the side-chain orientation of the mimetics within the bivalent compounds. We generated a combinatorial library of bivalent peptidomimetics on a triazine core, and a pharmacophore based on β-turns from NT-3, NGF and BDNF. The resulting compounds were tested in binding and biological screens which identified peptidomimetics blocking ligand-dependent receptor activity of TrkC or TrkA receptors, but which do not affect TrkB receptors.

## Materials and Methods

### Cells

NIH-3T3 cells are mouse fibroblasts that do not express any neurotrophin receptors. Parental NIH-3T3 cells were transfected with the indicated receptors. Stable clones of NIH-TrkC express ∼100,000 TrkC receptors/cell, NIH-TrkA express ∼200,000 TrkA receptors/cell, and NIH-IGF-1R express ∼100,000 insulin-like-growth factor-1 (IGF-1) receptors/cell. These cells, and their functional responses to the appropriate growth factor have been reported [17].

Neuronal PC12 express TrkA and p75 neurotrophin receptors and respond to NGF. The nnr5-TrkC cells are a variant of PC12 that lost TrkA expression, and into which human TrkC cDNA was stably transfected, and these cells respond to NT-3 [6], [22]. The 4-3.6 cells are B104 rat neuroblastoma stably transfected with human TrkA cDNA and express equal levels of p75 and TrkA (TrkA^+^ p75^+^) [23]. SY5Y-TrkB cells are human neuroblastoma SY5Y stably transfected with TrkB.

### Cell Survival Assays

The growth/survival profile of the cells were quantified in 96-well plates using the tetrazolium salt reagent 4-[4,5-dimethylthiazol-2-yl]-2,5-diphenyltetrazolium bromide (MTT; Sigma) 48–72 hours after plating; by reading the optical density (OD), as previously described [24]. Cells cultured in serum-free-medium (SFM) die by apoptosis with well-established kinetics, but they can be rescued if they are supplemented with the appropriate growth factor. NGF protects TrkA-expressing cells from death, and NT-3 protects TrkC-expressing cells from death. Cells did not receive growth factor supplementation, or were supplemented with suboptimal or with optimal concentrations of the indicated growth factor. Test peptidomimetics or vehicle controls were added to the culture conditions above, for 72 h. Cellular controls are provided by lack of effect on some cells with effect on other cells. Additional controls for lack of general toxicity were done by testing compounds on all cells growing in normal serum conditions (data not shown). All assays were repeated independently at least three times (n = 4 for each assay). MTT data are standardized to optimal dose of neurotrophin = 100% survival, and serum-free medium (SFM) = 0% survival, using the formula [(OD_test_−OD_SFM_)×100/(OD_optimal NTF_−OD_SFM_)], with a signal-noise ratio >4.

### Primary Neuronal Cultures

Cortex from day 14 C57/BL6 embryos were dissected and placed on ice-cold HHGN dissection solution (1X HBSS (Cellgro 20-021-CV), 2.5 mM HEPES (pH 7.4), 35 mM glucose, 4 mM sodium bicarbonate). They were then resuspended in 3 ml of Trypsin 0.125% in HHGN and incubated for 20 minutes at 37°C. Trypsinization was stopped with fetal calf serum. The tissue was collected and resuspended in 1 mL of culture medium, triturated with 5× passes on a 1000P pipette, then 5× passes each in a 3 ml syringe with 18G, 20G and 22G needles. The primary cortical neurons were counted and plated in a 6 well-plate at 500,000 cells/well in Neurobasal media containing B27 supplement, Penicillin/Streptomycin and Glutamax. After 48 h neuronal cultures were treated with vehicle, 2 nM NT-3 alone, or NT-3+10 µM compounds for 20 min. Cells were then lysed in detergent for biochemical studies.

### Signal Transduction Assays

Cells were stimulated with vehicle (negative control), suboptimal concentrations of growth factor alone (positive control), or growth factor plus compounds at 10 µM for 20 min. Detergent lysates were resolved in SDS-PAGE and analyzed by Western blotting with anti-phosphotyrosine (pTyr) antibody 4G10 (Upstate Biotechnology, Lake Placid, NY), anti-phospho-p44/42 MAPK (Thr 202/Tyr204) (Cell Signaling Technology), or anti-phospho-Akt (Ser473) antibody (Cell Signaling Technology). After stripping, membrane was probed with anti-actin (Sigma) to standardize protein loading. Total TrkC was also identified with highly selective monoclonal antibody 2B7 [6], [17], [18], [22]. Quantification was done from each membrane versus actin loading control by densitometric analysis [23].

### Neuritogenic Differentiation assay

The assays were performed as described [22]. Briefly, nnr5-TrkC cells were plated at low density in complete medium and allowed to adhere to the dish. Then the cells were treated with vehicle ± peptidomimetics or the appropriate growth factor ± peptidomimetics for 72 h. The morphology of the cells was scored from pictures taken randomly in a blinded fashion. Neurite outgrowth was determined as percentage of cells with neurites (defined as length >2 cell bodies).

### Fluorescent Activated Cell Scan (FACS) analysis

The assays were performed as described [18]. Briefly, cells expressing the indicated receptor were first bound, at 4°C, with biotin-tagged peptidomimetics (20 µM), washed, followed by addition of fluorescein-avidin at 4°C. After washing, cells were analyzed by FACScan/CellQuest. The mean channel fluorescence (MCF) background of NIH-IGF-1R was subtracted to analyze the specific MCF binding to test cells.

## Results

### Design and Synthesis of peptidomimetics

**Figure 1:**
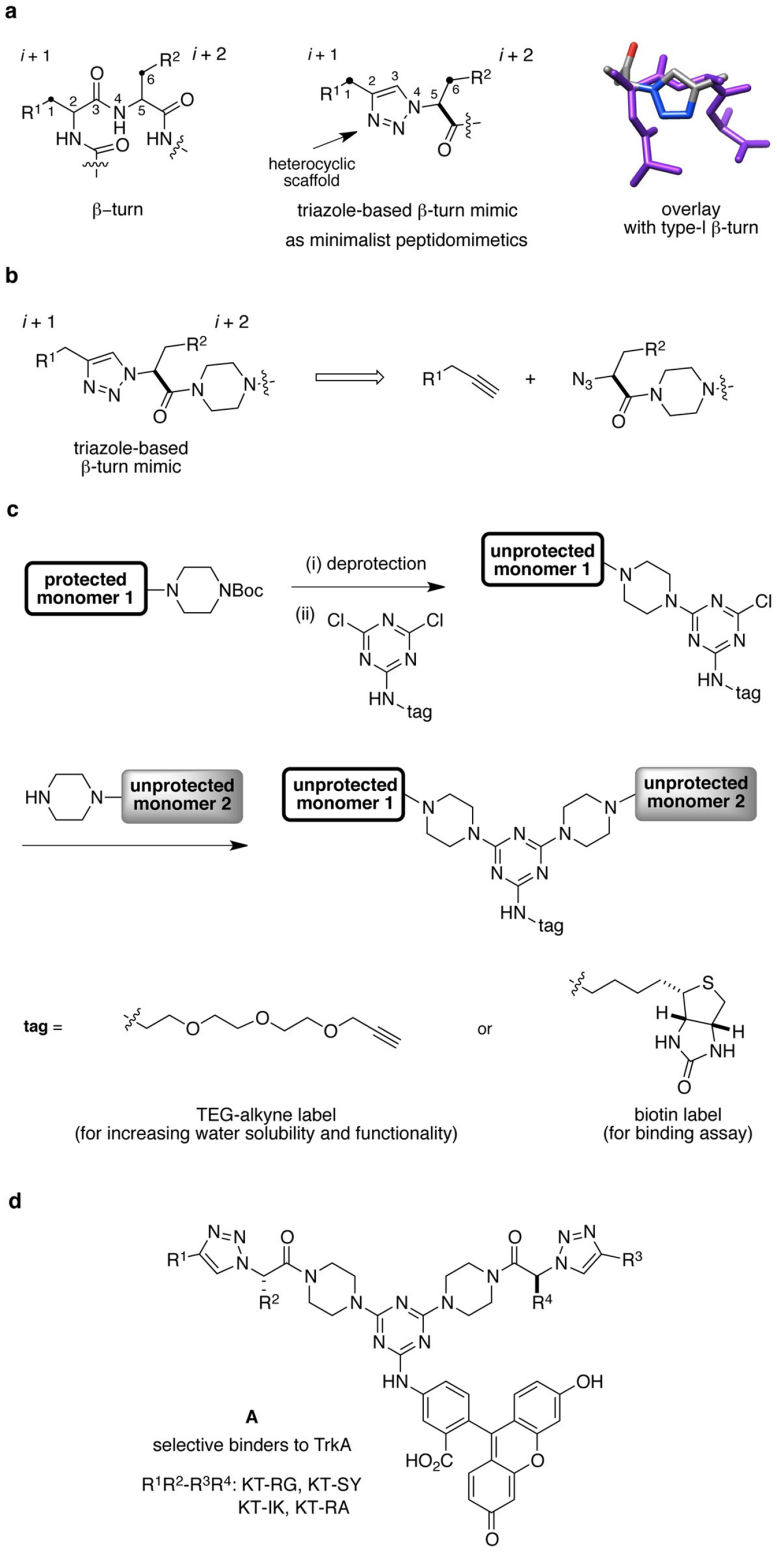
Compound design and chemistry. (**a**) Design of minimalist peptidomimetics with triazole scaffolds, and overlay of triazole-based mimic with type-I β-turn. (**b**) Strategy to build triazole-based β-turn mimics by click chemistry. (**c**) A general scheme to prepare bivalent mimics. (**d**) Structures of compounds, **A** that bind to TrkA.

Minimalist mimics express only side-chains of secondary structures and a heterocyclic scaffold is used instead of a peptide backbone (Figure 1a) [25]. The scaffold gives rigidity to limit the degrees of freedom for bond rotations but, ideally, without excluding conformations that correspond the targeted secondary structures. Our first minimalist mimics were built on triazole-based scaffolds as β-turn mimic (Figure 1b) [19]. The triazole core was obtained by click chemistry between azido amino acids and alkynes having side-chains of amino acids (Figure 1c). The triazole-based β-turn mimics have piperazine linkers to build bivalent peptidomimetics that are obtained by S*_N_*Ar reactions on a triazine. Combinatorial methods shown in Figure 1d were used to combine two different monovalent units to give bivalent mimics (hetero- or homo-bivalent mimics). This combinatorial method has several advantages. First, combination of *n* monovalent compounds gives *n*(*n*+1)/2 bivalent compounds; a lot of bivalent mimics can be made from relatively few monovalent ones. Second, the products can be formed without using any protecting group on monovalent components. Finally, the third site in triazine core can be used to support tags for biological assays, e.g. dyes, biotin, and polyethylene glycol (PEG). Previously we reported the first triazole-based β-turn bivalent mimics **A** selectively bind to TrkA just as NGF does, but their activities in cellular assays are weak. Thus, this present study focused on discovering related compounds to improve binding to “Trk” receptors and the activities of these compounds in cellular assays.

**Figure 2:**
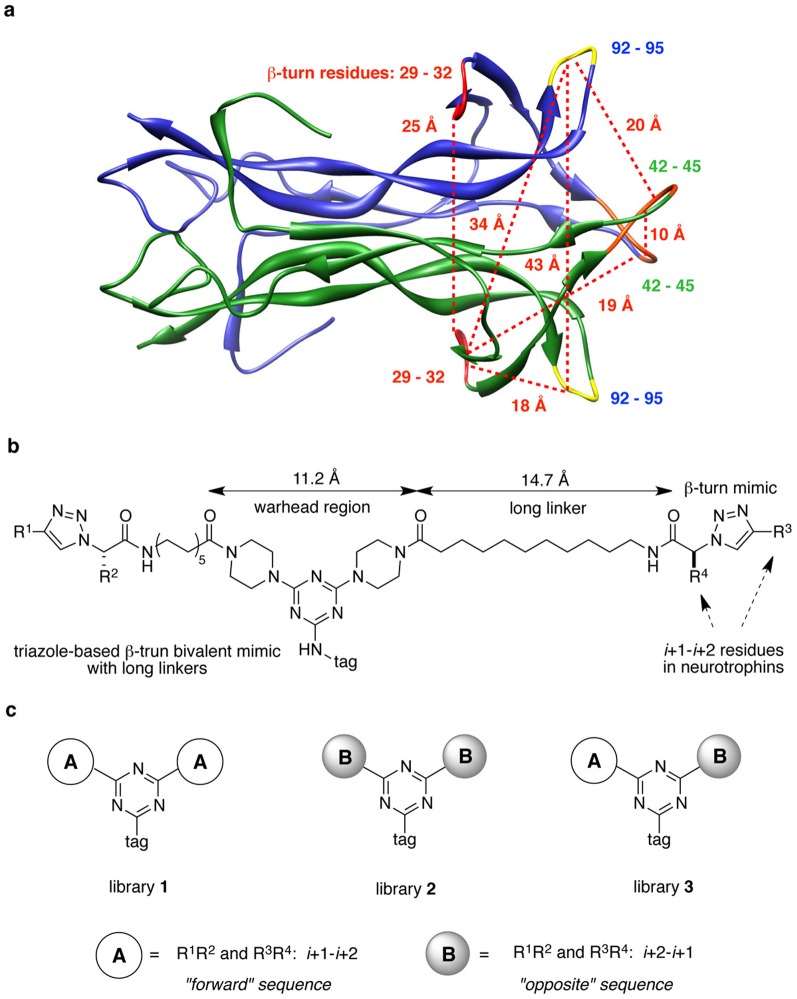
Relative structure of mimetics within neurotrophin proteins. (**a**) Distances between hot-spots (highlighted with red, orange, and yellow) in NT-3. (**b**) General structures of triazole-based β-turn mimics with long linkers, and lengths of warhead region and linker. (**c**) Strategy to build bivalent mimics with different orientation sequences in triazole core.

In particular, we were interested in using long-linkers between triazole core and triazine core in bivalent mimics to provide more distance variation for separation of the monovalent units on binding. Distances between hot-spots in neurotrophins are from 10 Å to 43 Å (Figure 2a). Consequently, we reasoned that if each monovalent mimic were connected to flexible chains of about 15 Å length, the total separation would be about 41 Å maximum, and the warhead regions could orient like any pair of turns in neurotrophins (Figure 2b). This strategy might especially increase the chances for mimicry of β-turns that are far from each other. While a flexible linker has an entropic cost, the cooperative binding of a bivalent ligand could compensate and enhance the affinity.

Another new line of investigation for this study was to use side-chains on the peptidomimetics that correspond exactly to the *i*+1 and *i*+2 residues in the neurotrophins turn regions [19]. Thus, in the present work, seven dipeptide sequences corresponding to *i*+1 and *i*+2 residues in the neurotrophins were targeted; IK, EK, and GK from NGF, MS and SK from BDNF, and IR and TG from NT-3 (Table S1 in Information S1, highlighted with red). Sequences that contain asparagine, e.g. NN or NK from NGF and NT-3 were not chosen because of synthetic difficulty.

A third variable tested in this study was the orientation of the side-chains in the mimics. Monovalent mimics with the long linkers were prepared in two different orientations, e.g. “forward” and “opposite” sequences. The forward sequence follows the orientation of natural ligands, *N*- to *C*-terminal (R^1^R^2^ and R^3^R^4^ = *i*+1-*i*+2, e.g. TG), but the opposite sequence has the orientation of *C*- to *N*-terminal (R^1^R^2^ and R^3^R^4^ = *i*+2-*i*+1, e.g. GT). Therefore, bivalent mimics by combination of the monovalent mimics were prepared in three different library sets; library **1** (forward only): R^1^R^2^ and R^3^R^4^ = *i*+1-*i*+2 and *i*+1-*i*+2, e.g. TG-TG, library **2** (opposite only): R^1^R^2^ and R^3^R^4^ = *i*+2-*i*+1 and *i*+2-*i*+1, e.g. GT-GT, and library **3** (forward-opposite): R^1^R^2^ and R^3^R^4^ = *i*+1-*i*+2 and *i*+2-*i*+1, e.g. TG-GT as Figure 2c.

A library of 120 bivalent compounds with either a triethylene glycol (TEG) based label or biotin label was prepared from 14 monovalent compounds and morpholine using a procedure similar to a previously reported one [19], [20]. The morpholine was used as a control for triazole-based mimics, and a linker part.

The modified reaction schemes for monovalent and bivalent peptidomimetics are described in Supplemental (Tables S2 and S3 in Information S1). The purification and characterization procedures and data (HPLC and ^1^H-NMR and ^13^C-NMR) for monovalent mimics are in Supplemental (Section C in Information S1). The morpholine linker was used as a control for triazole-based mimics.

### Selective inhibition of TrkC and TrkA mediated cell survival

A total of 134 monomeric and the corresponding bivalent peptidomimetics including control compounds were tested in cell survival assays. Cells undergo apoptotic death when cultured in serum-free medium (SFM). Growth-factor protection from death in SFM is dose-dependent and time-dependent. Optimal concentrations of growth factor (4 nM) afford maximal protection standardized to 100%. Suboptimal concentrations of growth factor (0.2 nM) protect at ∼30–40% of maximal.

**Figure 3:**
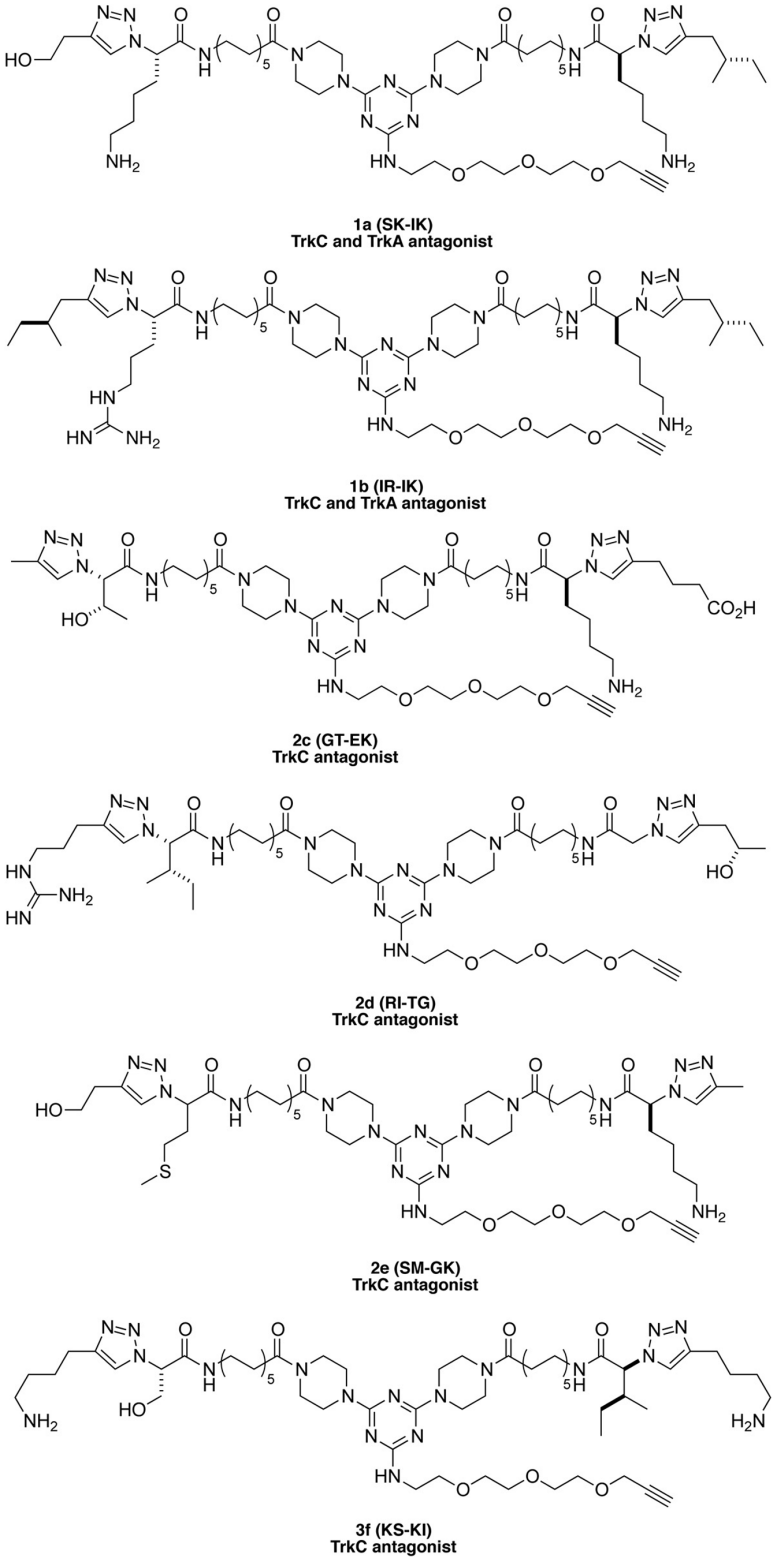
Structures of antagonists for TrkC or TrkA.

**Figure 4:**
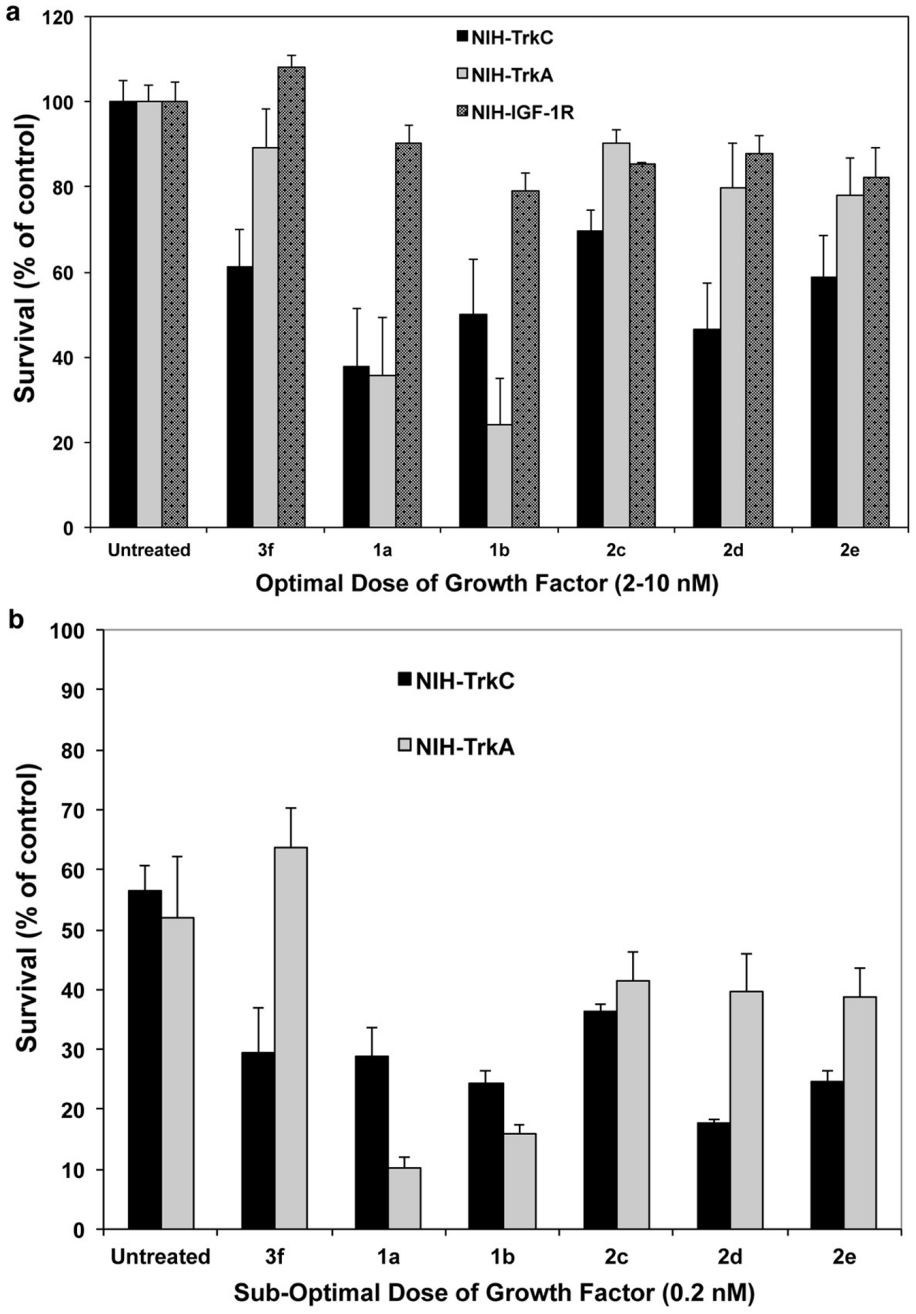
Selective antagonism of ligand-dependent cell survival. NIH-3T3 cells expressing either TrkA or TrkC or IGF-1R were cultured in SFM supplemented with the indicated peptidomimetic (10 µM) with optimal (**a**) or suboptimal (**b**) concentrations of the appropriate growth factor for 72 h. Survival was measured in MTT assays, and was calculated relative to optimal growth factor-mediated survival (100%). Results shown are average + SEM, from at least three independent experiments. One-way ANOVA, p<0.05 considered significant. All “hits” shown were significant for inhibition of TrkC, only **1a** and **1b** “hits” were significant for inhibition of TrkA.

The peptidomimetics were tested for their effect on NGF or NT-3-mediated survival. From the 134 compounds, **1a**, **1b**, **2c**, **2d**, **2e**, **3f** (Figure 3) significantly reduce the survival of NIH-TrkC cells responding to the cognate growth factor NT-3 at both optimal and suboptimal doses (Figure 4) (p<0.05). In counter-assays testing NIH-TrkA cells responding to the cognate growth factor NGF, compounds **2c**, **2d**, **2e**, **3f** have no effect (hence they are selective for TrkC). However, compounds **1a**, **1b** significantly reduce the survival of NIH-TrkA cells induced by NGF at both optimal and suboptimal doses (Figure 4) (p<0.05).

In controls, none of these compounds had an effect on the survival of NIH-IGF-1R cells responding to a different growth factor, IGF-1 (Figure 4a). Lack of effects on the growth of NIH-IGF-1R cells responding to IGF-1 indicate a selective reduced metabolic activity of NIH-TrkC cells responding to NT-3 or NIH-TrkA cells responding to NGF. This is likely due to reduced TrkC or TrkA signaling.

The selectivity of the inhibition of ligand-dependent survival suggests lack of generalized toxicity. In addition, lack of general toxicity was further determined because when the three cells lines are grown in normal serum there is no effect on growth (data not shown) and when the three cells lines are grown in serum-free medium without supplemented growth factors there is not accelerated death above that seen in serum-free medium (data not shown).

### Co-expression of p75 does not influence the antagonism of peptidomimetics

**Figure 5:**
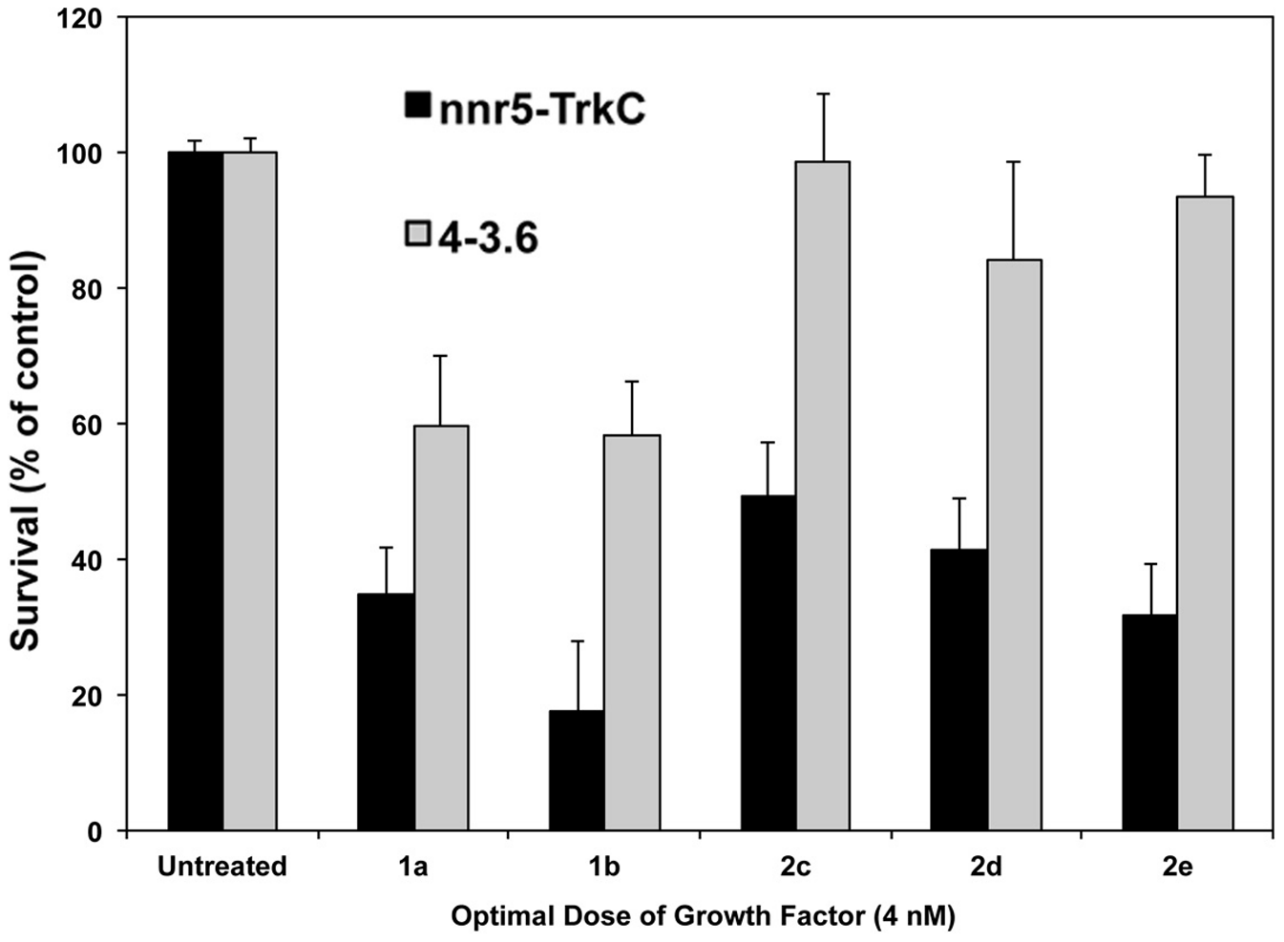
Co-expression of p75-receptors does not influence antagonism of ligand-dependent cell survival. Neuronal nnr5-TrkC cells expressing (TrkC and p75) and 4-3.6 cells (TrkA and p75) were cultured in SFM supplemented with the indicated peptidomimetics (10 µM) with or optimal concentration of the appropriate growth factor for 72 h. Survival was measured in MTT assays, and was calculated relative to optimal growth factor-mediated survival (100%). Results shown are average + SEM, from at least three independent experiments. One-way ANOVA, p<0.05 considered significant. All “hits” shown were significant for TrkC, only **1a** and **1b** “hits” were significant for inhibition of TrkA.

MTT survival data in nnr5-TrkC cells (expressing TrkC and p75) and rat neuroblastoma 4-3.6 cells (expressing TrkA and p75) are similar to the data in fibroblast cells NIH-TrkC, and NIH-TrkA, indicating that p75 co-expression does not interfere in the antagonism of survival by these peptidomimetics (Figure 5).

### Selective inhibition of TrkC and TrkA-mediated signal transduction

To confirm the antagonistic activity of these compounds we analyzed signal transduction in biochemical assays. The tyrosine phosphorylation of the Trk receptors (p-Trk), the phosphorylation of Akt (p-Akt), and the phosphorylation of MAPK (p-MAPK) were studied by western blots of cell lysates prepared after stimulation of cells with the appropriate growth factor ± compounds for 20 min. The 20 min time point was chosen because NT-3 and NGF are known to induce sustained activity of these signal pathways, but without causing downregulation or internalization of the Trk receptors that could affect the overall signals [22].

**Figure 6:**
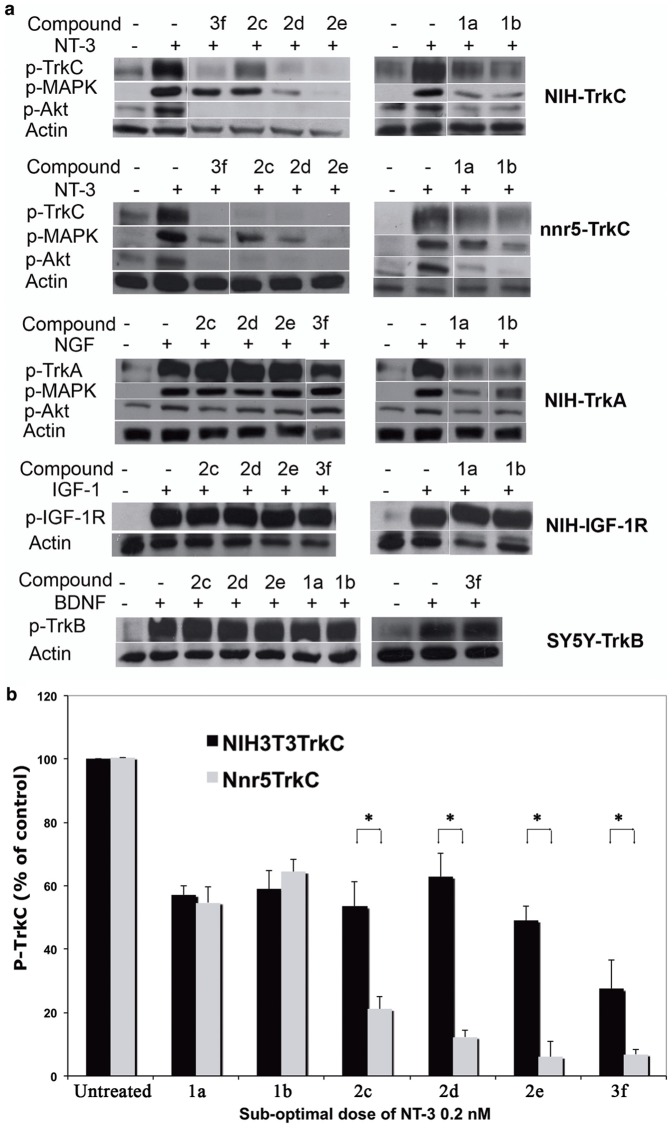
Antagonists of TrkC or TrkA receptor inhibit ligand-dependent signaling pathways in cell lines. (**a**) NIH-TrkC (a), nnr5-TrkC (b), NIH-TrkA (c), NIH-IGF-1R (d) and SY5Y-TrkB (e) cells were exposed to the indicated peptidomimetic (10 µM) and NT-3 (0.2 nM) (a, b), NGF (0.2 nM) (c), IGF-1 (10 nM) (d) and BDNF (0.2 nM) (e) for 20 min. Detergent lysates were analyzed by Western blotting with anti-pTyr mAb 4G10 or anti-phospho-MAPK or anti-phospho-Akt. After stripping, membrane was re-probed with anti-actin antibodies to standardize loading. (**b**) Quantification of p-TrkC in NIH-TrkC and nnr5-TrkC by densitometry from 3 independent experiments. Data are expressed as the mean + SEM relative to growth factor. One-way ANOVA, p<0.05 considered significant. All “hits” shown were significant for inhibition of TrkC compared versus drug-untreated. * indicates differences in efficacy comparing the two cell lines.

In NIH-TrkC cells NT-3 induces strong p-TrkC, p-Akt and p-MAPK. **1a**, **1b**, **2c**, **2d**, **2e**, **3f** decreased significantly these ligand-dependent signals (p<0.05). Similar results were obtained in nnr5-TrkC expressing p75, indicating that p75 co-expression may not interfere with antagonism by the compounds. However, the inhibition of TrkC signals was significantly more pronounced in nnr5-TrkC compared versus NIH-TrkC cells (Figure 6). This may be because NIH-TrkC cells express >3-fold more TrkC receptors/cell than nnr5-TrkC cells [17], [20].

As expected from the cell survival assays, **2c**, **2d**, **2e**, **3f** do not reduce the p-TrkA activation by NGF. However, **1a**, **1b** reduce p-TrkA by NGF. In controls, none of the compounds affect the activating signals that IGF-1 affords through IGF-1R (Figure 6).

In further counter-assays, we also studied TrkB receptor activation by BDNF in neuronal cells SY5Y-TrkB. These peptidomimetics do not reduce the pTrkB activation by BDNF (Figure 6a), further indicating their selectivity for TrkC and TrkA.

These biochemical data are consistent with the bioassays, and indicate that an NT-3 or NGF derived peptidomimetics can inhibit NT-3-dependent or NGF-dependent activation and tyrosine phosphorylation of the target receptor. Reduced signals downstream of the receptor result in lower cell survival in bioassays.

**Figure 7:**
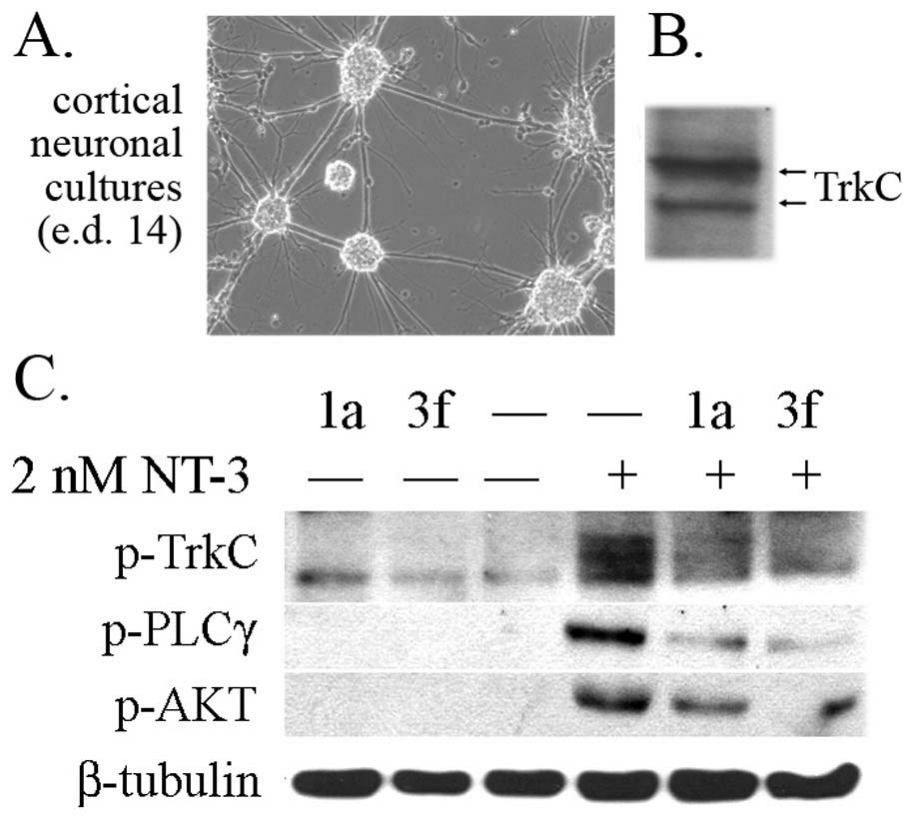
Antagonists of TrkC receptor modulate ligand-dependent signaling pathways in primary neuronal cultures. Primary neuronal cultures were prepared from the cortex of embryonic day 14 mice (ed 14). (A) representative picture of a culture. (B) TrkC is detected in western blots of lysates obtained from these neurons, using anti-TrkC mAb 2B7. (C) Treatment with NT-3 (2 nM for 20 min) induces biochemical signals (p-TrkC, p-PLCγ, p-AKT) that are inhibited by co-treatment with compounds **1a** or **3f** (10 µM). In the absence of NT-3, the compounds did not have an effect on signals. Control β-tubulin III is loading standard.

To verify the data above, similar assays were done using primary neuronal cultures prepared from the cortex of embryonic day 14 mice (Figure 7). These neurons express TrkC (Figure 7b). Treatment with NT-3 (2 nM for 20 min) induces biochemical signals (p-TrkC, p-PLCγ, p-AKT) that are inhibited by co-treatment with 10 µM TrkC antagonistic compounds **1a** or **3f** (Figure 7c). In the absence of NT-3 compounds did not induce signals (Figure 7c), suggesting that they are not partial agonists or inverse antagonists.

### Lack of effect in differentiation assays

**Figure 8:**
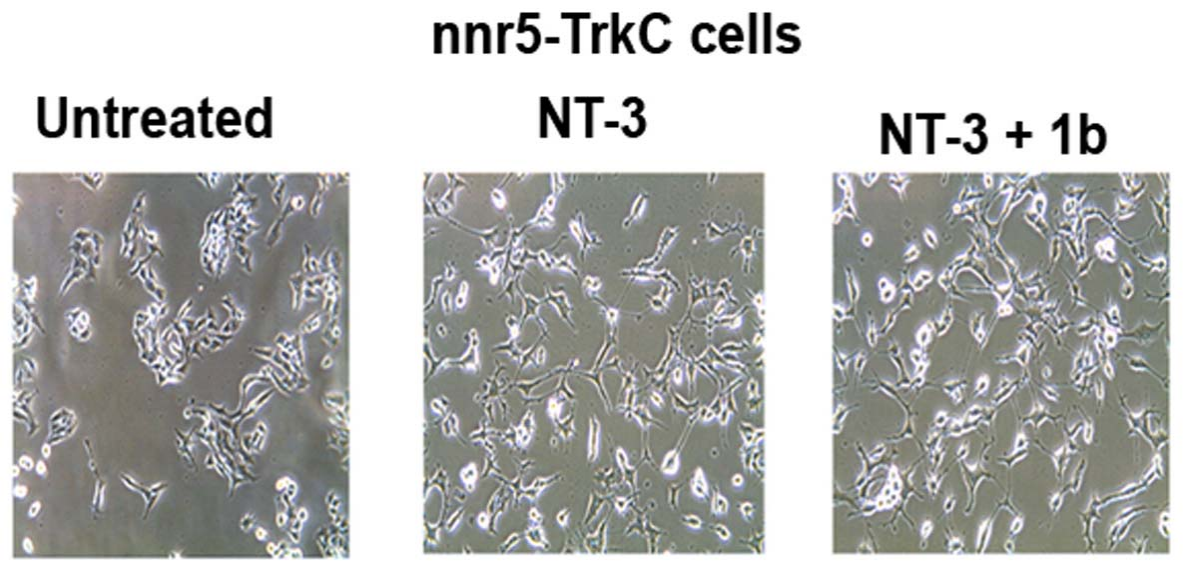
Antagonists do not affect ligand-dependent neuritogenesis. Cells expressing TrkC and p75 (nnr5-TrkC) were plated in full medium ± neurotrophin ± peptidomimetics (10 µM). Fields were photographed 72 h later. Neurite outgrowth was determined as percentage of cells with neurites (defined as length >2 cell bodies). Representative pictures from nnr5-TrkC for compound **1b** are shown.

While **1a**, **1b**, **2c**, **2d**, **2e**, **3f** reduce the survival induced by NT-3, they do not inhibit the NT-3-induced differentiation of neuronal cells nnr5-TrkC. Representative pictures from nnr5-TrkC cells treated with compound **1b** are shown (Figure 8).

### Binding assays with biotin-tagged compounds

The peptidomimetics were tagged with biotin in order to perform binding studies. The new biotin-compounds are named **1a′**, **1b′**, **2c′**, **2d′**, **2e′**, **3f′** and are respectively equivalent to **1a**, **1b**, **2c**, **2d**, **2e**, **3f**.

**Figure 9:**
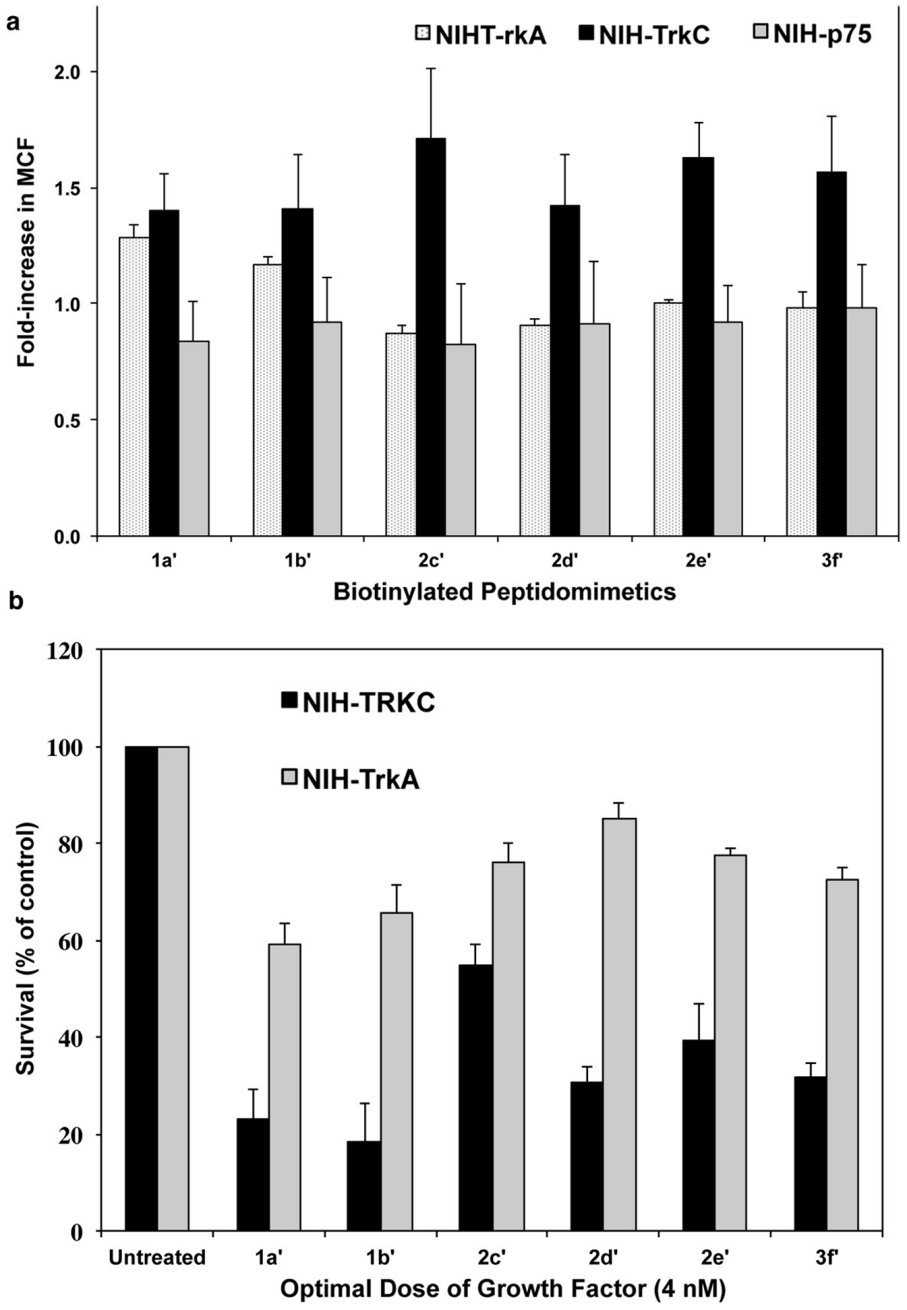
Biotinylated peptidomimetics have similar activity as untagged compounds. (**a**) Cells expressing the indicated receptor were exposed at 4°C to biotinylated ligand (20 µM), followed by fluorescein-avidin. After washing, cells were analyzed by FACScan/CellQuest. The background MCFs of NIH-IGF-1R were subtracted to analyze the specific MCF binding to test cells. Results shown are average MCF+SEM, n = 3 independent experiments. ANOVA, p<0.05 considered significant. All “hits” shown were significant for TrkC, only **1a′** and **1b′** “hits” were significant for TrkA. (**b**) Biotinylated peptidomimetics were tested in cell survival bioassays. NIH-3T3 cells expressing either TrkA or TrkC receptor were cultured in SFM supplemented the indicated peptidomimetics (5 µM) with or without optimal concentrations of the appropriate growth factor for 72 h, as indicated. Survival was measured in MTT assays, and was calculated relative to optimal growth factor-mediated survival (100%). Results shown are average + SEM, from at least three independent experiments. One-way ANOVA, p<0.05 considered significant (**a**, **b**). All “hits” shown were significant for inhibition of TrkC, only **1a′** and **1b′** “hits” were significant for inhibition of TrkA.

FACS binding indicated that **1a′**, **1b′**, **2c′**, **2d′**, **2e′**, **3f′** bind preferentially to TrkC receptor. **1a′**, **1b**′ also bind to TrkA but to a lesser degree than TrkC (Figure 9a).

To ascertain that replacement of TEG with biotin has no influence on the function of the compounds, **1a′**, **1b′**, **2c′**, **2d′**, **2e**′, **3f′** were also tested in biochemical and biological assays already described. The results are equivalent whether the compounds carry a TEG or a biotin tag (Figure 9b).

## Discussion

We report on the biological characterization of a library of 134 triazole-based peptidomimetics with the potential to mimic NT-3, NGF, or BDNF because they analog relevant β-turn sequences (*i*+1-*i*+2). Six bivalent mimics in the library showed biological activities for TrkA or TrkC, but none of the monovalent mimics and a morpholine bivalent mimic that was used as a control for triazole-based mimics showed any significant activities for Trk receptors. Bivalent mimics **2c**, **2d**, **2e**, **3f** are selective TrkC ligands, and **1a**, **1b** are TrkC and TrkA ligands in binding assays (FACS). The compounds act as antagonists in bioassays (survival in SFM) and in biochemical assays (receptor signal transduction). These peptidomimetics do not block the activity of IGF-1R or TrkB, and are not affected by p75 co-expression.

In previous work, we reported triazole-based β-turn bivalent mimics with related sequences in the β-turn region of neurotrophins, connected by a relatively rigid piperazine linker [19]. Four peptidomimetics showed selective binding activity for TrkA. However, the compounds did not show significant cellular activity in bioassays. To improve the cellular activity, and selectivity for Trk receptors, we modified the triazole-based β-turn mimics to use a long alkyl linker, *i*+1-*i*+2 sequences, and sequence orientation. We expected the long linker would give more chances to mimic β-turns that are far from each other. In direct comparison between the previous library and the present library, a triazole-based bivalent mimic with the same residue set, RI-TG, with **2d**, but a short linker (only piperazine linker) did not bind to any Trk receptors [19].

In addition, we hypothesized that amino acid side-chains corresponding to β-turn in neurotrophins might give selectivity of the compounds for Trk receptors. Comparison between sequences of active compounds and neurotrophins are summarized in Table 1.

**Table 1:** Comparison between sequences of the bivalent mimics and neurotrophins.

compounds	1a	1b	2c	2d	2e	3f
bind to	both TrkC and TrkA		only TrkC			
R^1^R^2^-R^3^R^4^	SK-**IK**	IR-**IK**	GT-**EK**	RI-TG	SM-**GK**	KS-**KI**

Bold letters in sequences correspond to sequences in NGF, normal letters correspond to BDNF, and letters that are underlined correspond to NT-3.

Compound **1b** binds to both TrkC and TrkA and have dipeptide sequences corresponding to *i*+1-*i*+2 of NGF and NT-3. Compound **1a** binds to both TrkC and TrkA also but has a dipeptide that corresponds to a turn in NT-3 (e.g. it does not have NGF sequences). Since NT-3 does bind to TrkA (albeit with lower affinity than NGF) it is possible that this dipeptide is involved in the binding. Both **1a** and **1b** follow the orientation found in the natural ligands (sequences *i*+1 and *i*+2, *N*-terminal to *C*-terminal).

Compounds **2c** and **2d**, which selectively bind to TrkC, have sequences corresponding to NT-3, the natural ligand of TrkC. However, **2e** and **3f** which also bind to TrkC selectively have sequences corresponding to NGF and BDNF, and this was unexpected because NGF binds TrkA and BDNF bind TrkB. This suggests that the sequences at the β-turns of neurotrophins may be promiscuous (Table S1 in Information S1). but constrained either by their orientation or by the overall conformation of the protein.

Overall, the data suggest that the orientation of the β-turn side-chains are relevant to the selectivity of these compounds. Consistent with the concept that orientation affects selectivity **2c** (GT-EK) and **2d** (RI-TG) share one set of side-chains but in the opposite orientation (sequences *i*+2 and *i*+1, *C*-terminal to *N*-terminal), and inversion of the sequence of **1a** (SK-IK) binding both TrkC and TrkA results in **3f** (KS-KI) with selectivity to TrkC. However, their hybrid mimics (SK-KI and KS-IK) do not show any significant binding or activity for the receptors.

Previously, we reported NT-3 mimics with partial agonistic activity. They are β-turn cyclic peptidomimetics [17], [26] with ring-fused C^1^- motif or β-turn triazole-based minimalist mimics [20] that have extra amino group on the triazole core, and side-chains that were commonly found at hot-spot interactions in general. Changes of the pharmacophores, such as the simplest triazole-based minimalist mimics, combinatorial assembly, different side-chains, and longer linker length, in the present work gave different biological effect, partial antagonists of TrkC, or both TrkC and TrkA.

Antagonism of ligand-dependent activation can be most easily explained in terms of competitive antagonism, meaning that the small molecule prevents the binding of the natural ligand to the receptor. Biological data are consistent with this notion. The fact that these peptidomimetics do not reduce the baseline TrkC or TrkA receptor activity in the absence of NT-3 or NGF further suggest that they act as ligand competitors. Unfortunately labeled NT-3 was not of a quality sufficient to perform direct competition studies.

The antagonism of these peptidomimetics is limited to ligand-dependent cell survival, because they do not affect neuritogenic differentiation. Based on the literature, we offer two possible explanations.

First, there are at least two receptor “hot-spots” associated with discrete biological activities [6], [22], [27]. Agonists binding to such hot-spots can activate one pathway (e.g. the “survival hot-spot”) but not to another (e.g. the “differentiation hot-spot”), and vice versa [17], [20]. Thus, it is possible that the mimetics herein prevent ligand binding to (or ligand activation of) one hot-spot of the receptor (e.g. the “survival hot-spot”) but not the other (e.g. the “differentiation hot-spot”).

Second, the discriminating effect upon survival versus differentiation may be related to the kinetics of receptor activation leading to each biological effect. Fast and transient kinetics of receptor activation transduce signals leading to survival, whereas slow and sustained kinetics of receptor activation transduce signals leading to differentiation [28], [29]. We speculate that the antagonists perhaps alter the kinetics of receptor activation, or perhaps alter the kinetics of receptor endocytosis or recycling [30], in such a way that survival is inhibited.

Conceptually, we demonstrate that altering side-chain, linker length, and sequence orientation of a neurotrophin pharmacophore can supply an easy modular approach to generate larger libraries with diversified bioactivity. We propose that the approach may be applicable to other target peptides and growth factors.

## Supporting Information

Information S1 Schemes and General Methods for Syntheses. Schemes and General Procedure and Preparation of Monovalent Mimics. Schemes and General Procedure and Preparation of Bivalent Mimics. Table S1. β-Turn Sequence in Neurotrophins. Table S2. Characterization of Compounds with TEG-alkyne Label. Table S3. Characterization of Compounds with Biotin.
(PDF)
