# Supplementary material for: Combinatorial Assembly of Small Molecules into Bivalent Antagonists of TrkC or TrkA Receptors
Source: PLoS One. 2014 Mar 6;9(3):e89617. doi: 10.1371/journal.pone.0089617 (PMC3945644; doi:10.1371/journal.pone.0089617)
Supplement: Information S1 — Schemes and General Methods for Syntheses. Schemes and General Procedure and Preparation of Monovalent Mimics. Schemes and General Procedure and Preparation of Bivalent Mimics. Table S1. β-Turn Sequence in Neurotrophins. Table S2. Characterization of Compounds with TEG-alkyne Label. Table S3. Characterization of Compounds with Biotin. (PDF) [file pone.0089617.s001.pdf]

SUPPORTING INFORMATION

Combinatorial Assembly of Small Molecules into Bivalent Antagonists of TrkC or TrkA Receptors

Fouad Brahimi [a,1], Eunhwa Ko [b,1], Andrey Malakhov [b], Kevin Burgess [b], and H. Uri Saragovi [a,c,d,*]

[a] Lady Davis Institute-Jewish General Hospital, Montreal, Quebec, Canada H3T 1E2

[b] Department of Chemistry, Texas A&M University. Texas, USA 77842.

[c] Department of Pharmacology and Therapeutics, McGill University, Montreal, Quebec, Canada H3T 1E2.

[d] Department of Oncology and the Cancer Center, McGill University, Montreal, Quebec, Canada H3T 1E2.

[1] equal first authors

[*] corresponding author. H.U. Saragovi (uri.saragovi@mcgill.ca)

TABLE OF CONTENTS

A. Table S1. β-Turn Sequence in Neurotrophins[1] 2
B. General Methods for Syntheses 2
C. General Procedure and Preparation of Monovalent Mimics 3
D. General Procedure and Preparation of Bivalent Mimics 41
E. Table S2. Characterization of Compounds with TEG-alkyne Label 42
F. Table S3. Characterization of Compounds with Biotin. 46
G. References 46

A. Table S1. β-Turn Sequence in Neurotrophins[1]

protein	source	β-turn sequence		
		30-33	44-47	93-96
NGF	murine	DIKG	INNS	DEKQ
	human	DIKG	INNS	DGKQ
	bovine	DIKG	INNS	DNKQ
	guinea pig	DIKG	VNNN	DGKQ
		30-33	44-47	93-96
BDNF	pig	DMSG	VSKG	DSKK
	human	DMSG	VSKG	DSKK
		29-32	42-45	92-95
NT-3	mouse	DIRG	KTGN	ENNK
	human	DIRG	KTGN	ENNK

The side-chains of monovalent mimics were *i*+1 and *i*+2 residues in the β-turn sequences of neurotrophins from different sources. Red color residues were chosen for this research. NN and NK were excluded because of synthetic issue.

B. General Methods for Syntheses

All reactions were carried out under an atmosphere of dry nitrogen. Glassware was oven-dried prior to use. Unless otherwise indicted, common reagents or materials were obtained from commercial source and used without further purification. All α-amino acids used were of the L-configuration. Triethylamine (TEA) was obtained anhydrous by distillation over calcium hydride and tetrahydrofuran (THF) was distilled over sodium metal and benzophenone. Dichloromethane (CH_2Cl_2) was dried by a Mbraun solvent drying system.

Flash column chromatography was performed using silica gel 60 (230-400 mesh). Analytical thin layer chromatography (TLC) was carried out on Merck silica gel plates with QF-254 indicator and visualized by UV. ^{1}H and ^{13}C NMR spectra were recorded on a Varian 500 (500 MHz ^{1}H; 125 MHz ^{13}C) spectrometer at room temperature. Chemical shifts were reported in ppm relative to the residual $CDCl_3$ (δ 7.27 ppm ^{1}H; δ 77.0 ppm ^{13}C), CD_3OD (δ 3.31 ppm ^{1}H; δ 49.86 ppm ^{13}C), or *d*^{6}-DMSO (δ 2.49 ppm ^{1}H; δ 39.5 ppm ^{13}C). NMR chemical shifts were expressed in ppm relative to internal solvent peaks, and coupling constants were measured in Hz. (br = broad)

Analytical HPLC analyses were carried out on 150 x 4.6 mm C-18 column using gradient conditions (10 – 90% B, flow rate = 0.75 mL/min). Preparative HPLC was carried out on 100 x 21.2 mm C-18 column using gradient conditions (10 – 70% B, flow rate = 10.0 mL/min). The eluents used were: solvent A (H_2O with 0.1% TFA) and solvent B (CH_3CN with 0.1% TFA).

C. General Procedure and Preparation of Monovalent Mimics

A total of 14 monovalent mimics were prepared by the following procedure in different sequence order of β-turn regions, e.g. *i*+1 and *i*+2 (TG) and *i*+2 and *i*+1 (GT).

Scheme S1. Syntheses of Monovalent Mimics

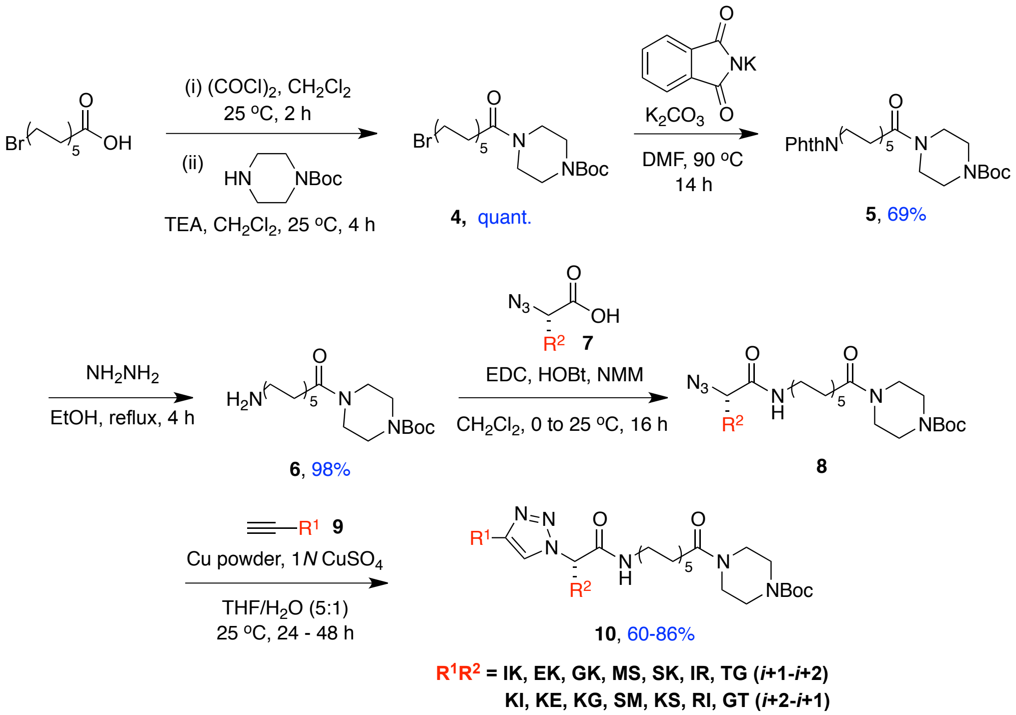

General Procedure for Compound 5

To a solution of 11-bromoundecanoic acid (1.0 eq.) in CH_2Cl_2 (1.0 M) was added oxalyl chloride (10.0 eq.). The mixture was stirred at 25 °C for 2 h, and then the solvent was removed. To remove the excess oxalyl chloride, the resulting residue was dissolved in CH_2Cl_2, and the solvent was removed under vacuum (x2). The resulting residue was dissolved in CH_2Cl_2 (1.0 M), and then Boc-piperazine (1.0 eq.), and TEA (2.5 eq.) were added to the solution. The mixture was stirred at 25 °C for 4 h. After the solvent was removed under vacuum, the reaction mixture was diluted with H_2O and extracted with EtOAc (x3). The combined organic phases were washed with brine and then dried over $MgSO_4$. After completely removing the solvent, the compound **4** was purified by flash chromatography (1:3 EtOAc/Hexanes). To a solution of compound **4** (1.0 eq.) in DMF (0.09 M) was added K_2CO_3 (1.05 eq.), and then the mixture was stirred at 90 °C for 14 h. The reaction mixture was diluted with H_2O and extracted with EtOAc (x3). The combined organic phases were dried over Na_2SO_4. After completely removing the solvent, the compound **5** was purified by flash chromatography (2:5 EtOAc/Hexanes).

Scheme S2. Synthesis of Compound **5**

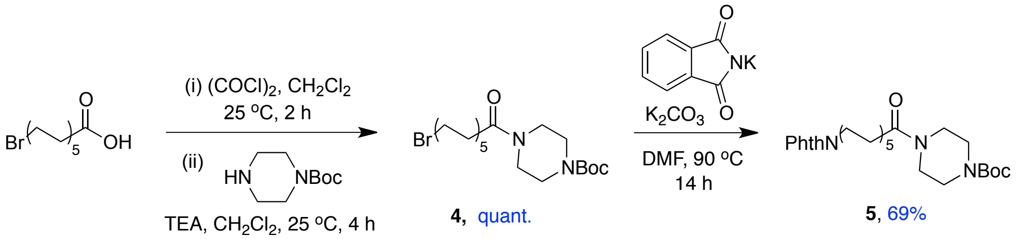

^{1}H NMR (500 MHz, DMSO-d_6) δ 7.83-7.80 (m, 4H), 3.53 (t, 2H, J = 7.0 Hz), 3.40-3.37 (m, 4H), 3.30-3.35 (m, 2H), 3.24 (br, 2H), 2.25 (t, 2H, J = 7.5 Hz), 1.58-1.51 (m, 2H), 1.46-1.42 (m, 2H), 1.39 (s, 9H), 1.24-1.20 (m, 12H)

^{13}C NMR (125 MHz, DMSO-d_6) δ 171.4, 168.6, 154.5, 135.0, 132.3, 123.7, 79.8, 45.3, 41.4, 38.0, 33.0, 29.6, 29.5(2), 29.4, 29.2, 28.7, 28.5, 26.9, 25.4

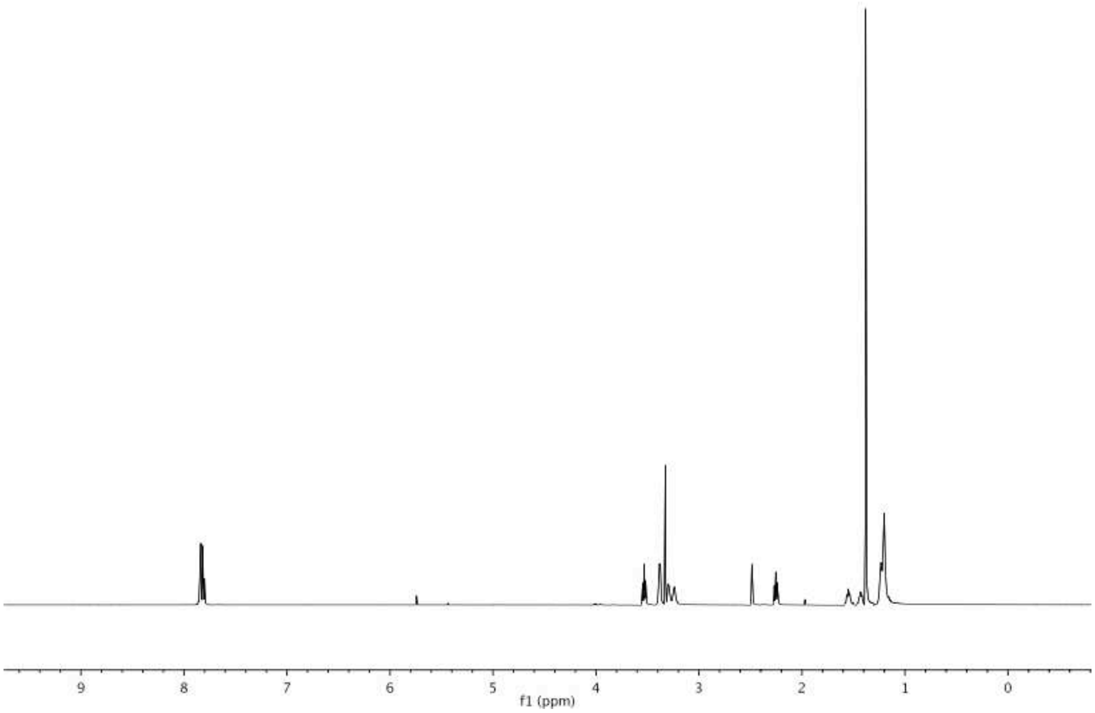

^{1}H NMR of **5**

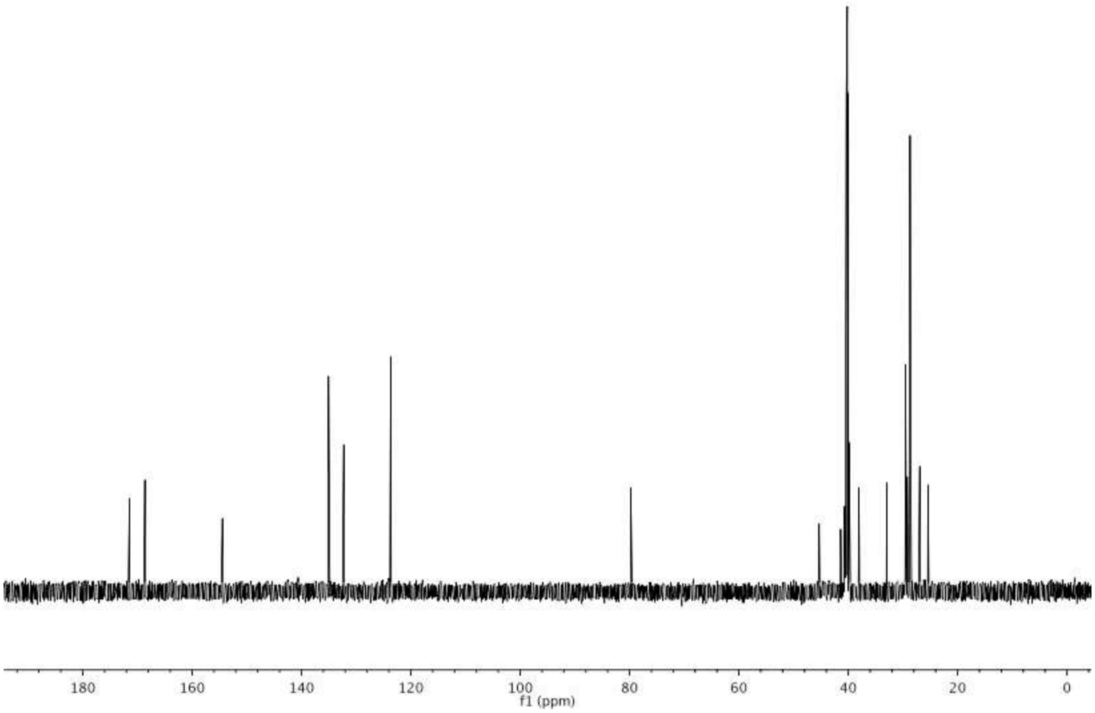

^{13}C NMR of **5**

General Procedure for Compound 6

Compound **5** (1.0 eq.) was dissolved in EtOH (0.16 M), and then hydrazine (4.0 eq.) was added to the solution. The mixture was refluxed at 90 °C for 4 h. After the reaction, the mixture was filtered, and then the solution was concentrated. Compound 6, thus obtained, was used for the next step without further purification.

Scheme S3. Synthesis of Compound **6**

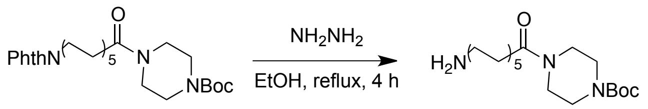

5 **6**, 98%

^{1}H NMR (500 MHz, DMSO-d_6) δ 3.40-3.38 (m, 4H), 3.30 (br, 2H), 3.24 (br, 2H), 2.48 (t, 2H, J = 5.5 Hz), 2.27 (t, 2H, J = 7.5 Hz), 1.45 (t, 2H, J = 6.0 Hz), 1.39 (s, 9H), 1.30 (t, 2H, J = 6.0 Hz), 1.22 (br, 12H)

^{13}C NMR (125 MHz, DMSO-d_6) δ 171.5, 154.5, 79.8, 45.3, 42.3, 41.4, 34.0, 33.0, 29.8, 29.7(2), 29.6, 29.5, 28.7, 27.1, 25.4

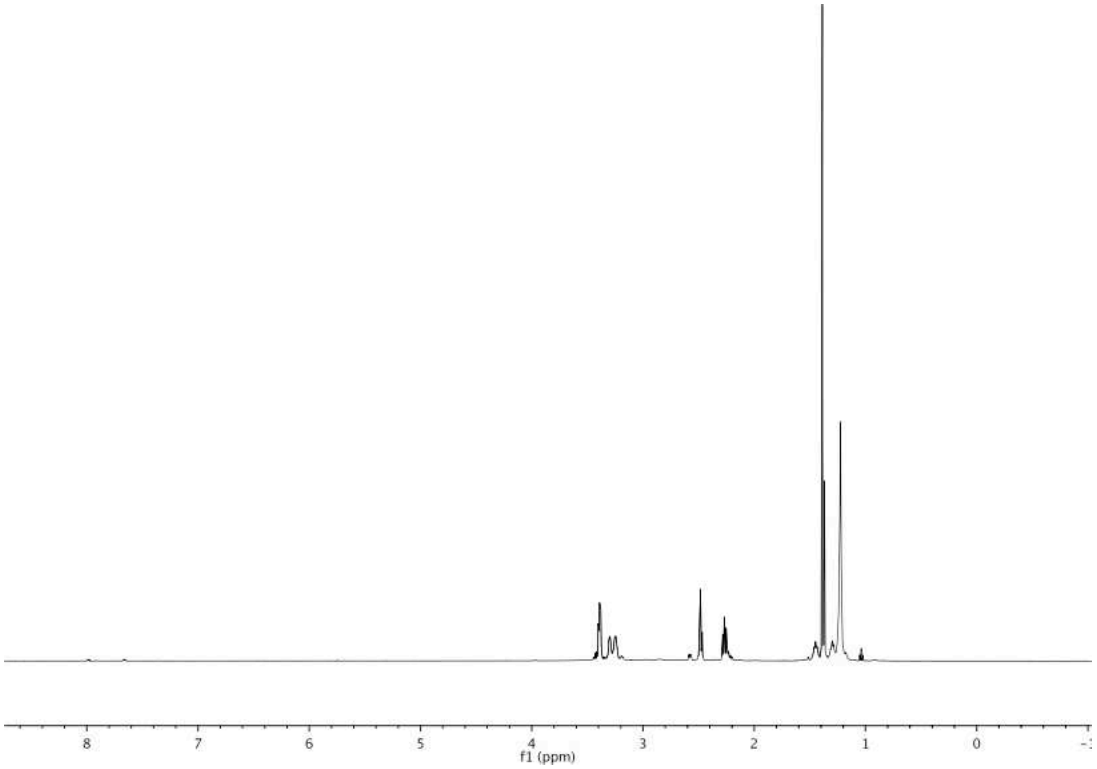

^{1}H NMR of **6**

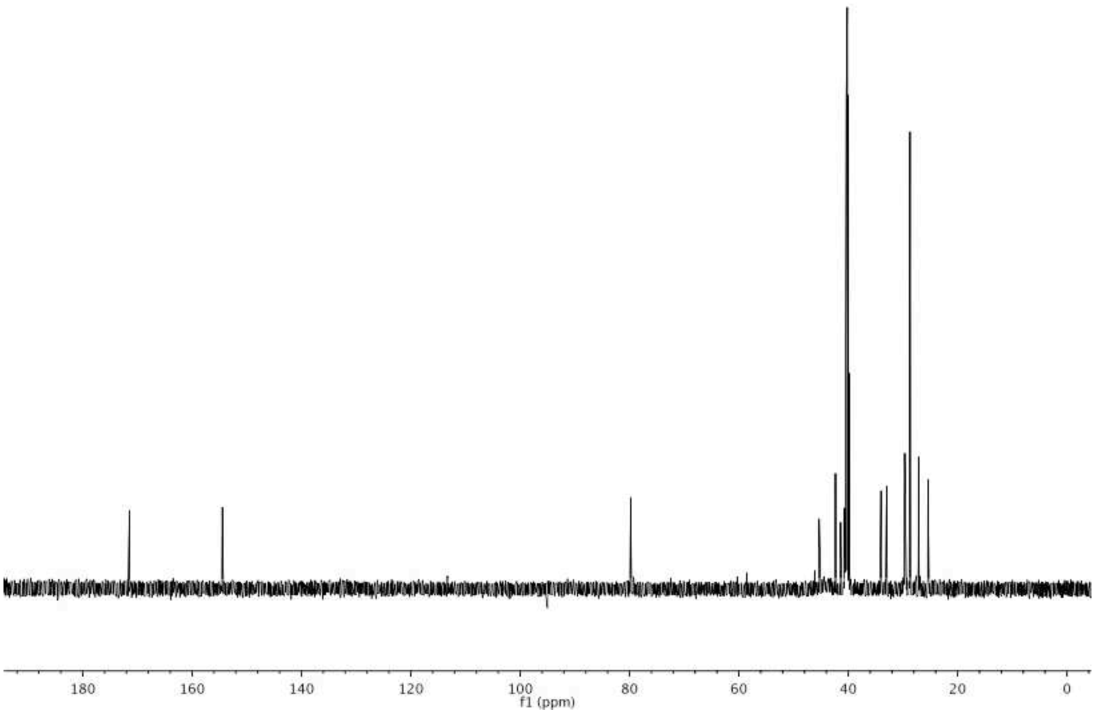

^{13}C NMR of **6**

General Procedure for Compounds 8

HOBt (1.1 eq.) and EDCI (1.1 eq.) were added to the solution of azide **7** (1.0 eq.) in CH_2Cl_2 (0.2 M) at 0 °C. The mixture was stirred at 0 °C for 15 min, and then NMM (2.0 eq.) and compound **6** (1.0 eq.) were added into the mixture. The solution was warmed up to 25 °C, and the mixture was stirred for 16 h. The solvent was removed, and then the resulting residue was diluted with H_2O and extracted with EtOAc (x3). The combined organic layer was washed with 5% HCl (aq.), followed by 5% Na_2CO_3 (aq.), and brine, and then dried over Na_2SO_4. After completely removing the solvent, the compound **8** derivatives were purified by flash chromatography (EtOAc/Hexanes).

Scheme S4. Synthesis of Compounds **8**

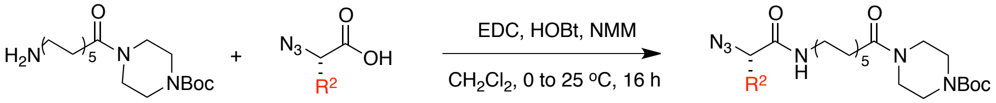

6 **7** **8**

R^2 = G, S, K, R, T, M, E, I

Compound 8_G

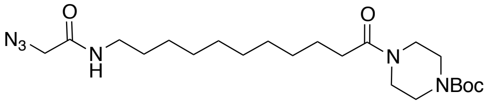

^{1}H NMR (500 MHz, $CDCl_3$) δ 6.44 (br, 1H), 3.93 (s, 2H), 3.56-3.53 (m, 2H), 3.43-3.34 (m, 6H), 3.25 (td, 2H, J = 12.0 Hz, J = 21.5 Hz), 2.29 (t, 2H, J = 12.5 Hz), 1.63-1.53 (m, 2H), 1.50-1.48 (m, 2H), 1.43 (s, 9H), 1.25 (br, 12H)

^{13}C NMR (125 MHz, $CDCl_3$) δ 172.1, 166.7, 154.8, 80.5, 52.9, 45.6, 41.5, 39.7, 33.6, 29.6(3), 29.4(2), 29.3,28.6, 27.0, 25.5

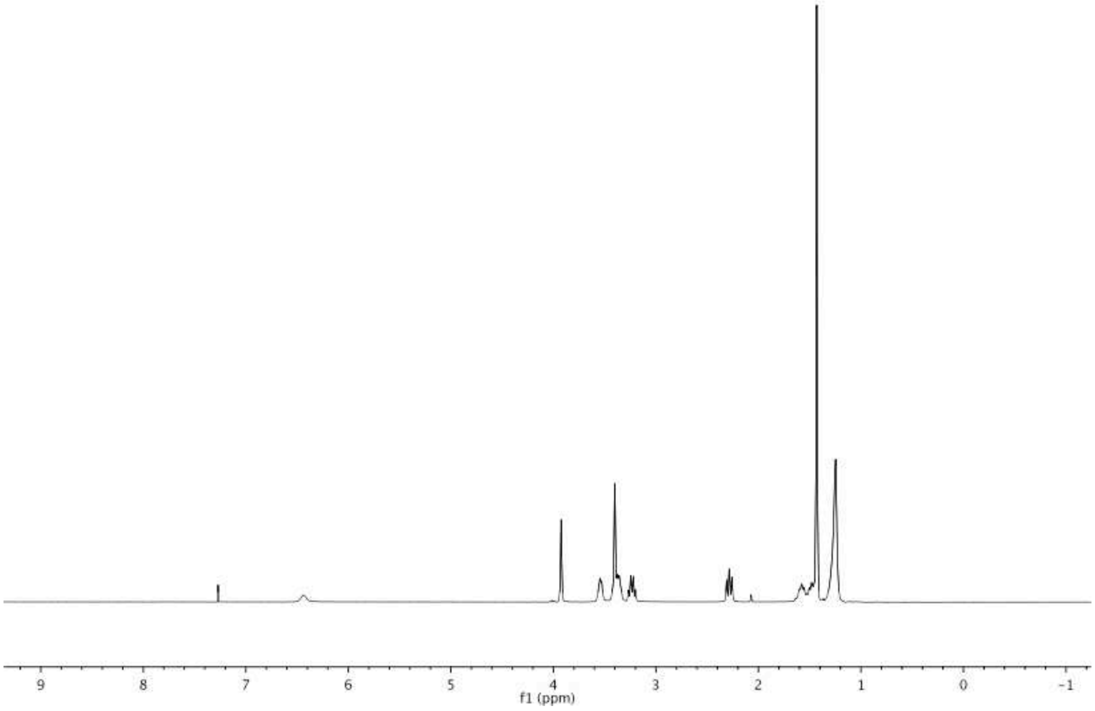

^{1}H NMR of **8_G**

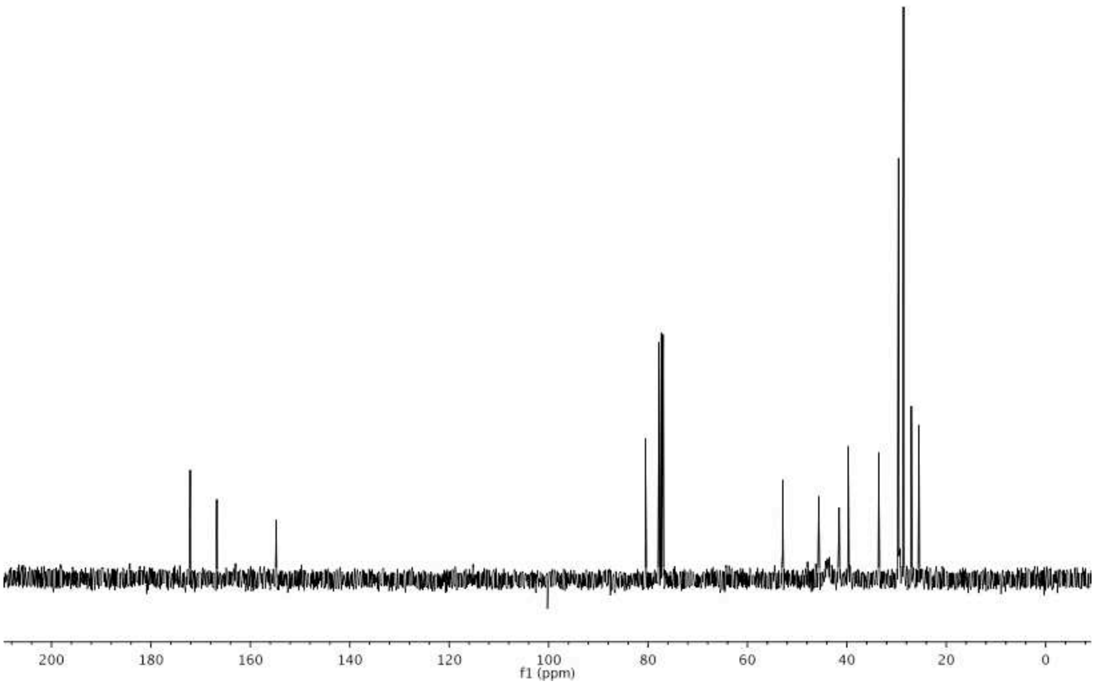

^{13}C NMR of **8_G**

Compound 8_S

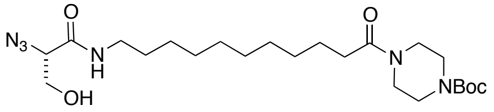

^{1}H NMR (500 MHz, $CDCl_3$) δ 6.56 (br, 1H), 4.08-4.01 (m, 1H), 3.97-3.91 (m, 1H), 3.60-3.56 (m, 2H), 3.43 (s, 4H), 3.41-3.37 (m, 2H), 3.33 (br, 1H), 3.28 (td, 2H, J = 9.5 Hz, J = 21.5 Hz), 2.31 (t, 2H, J = 14.5 Hz), 1.86 (br, 1H), 1.67-1.58 (m, 2H), 1.56-1.47 (m, 2H), 1.46 (s, 9H), 1.27 (br, 12H)

^{13}C NMR (125 MHz, $CDCl_3$) δ 172.3, 168.1, 154.8, 64.9, 63.6, 45.7, 41.6, 39.7, 33.6, 31.8, 29.6, 29.5(3), 29.4, 29.3, 28.6, 26.9, 25.4

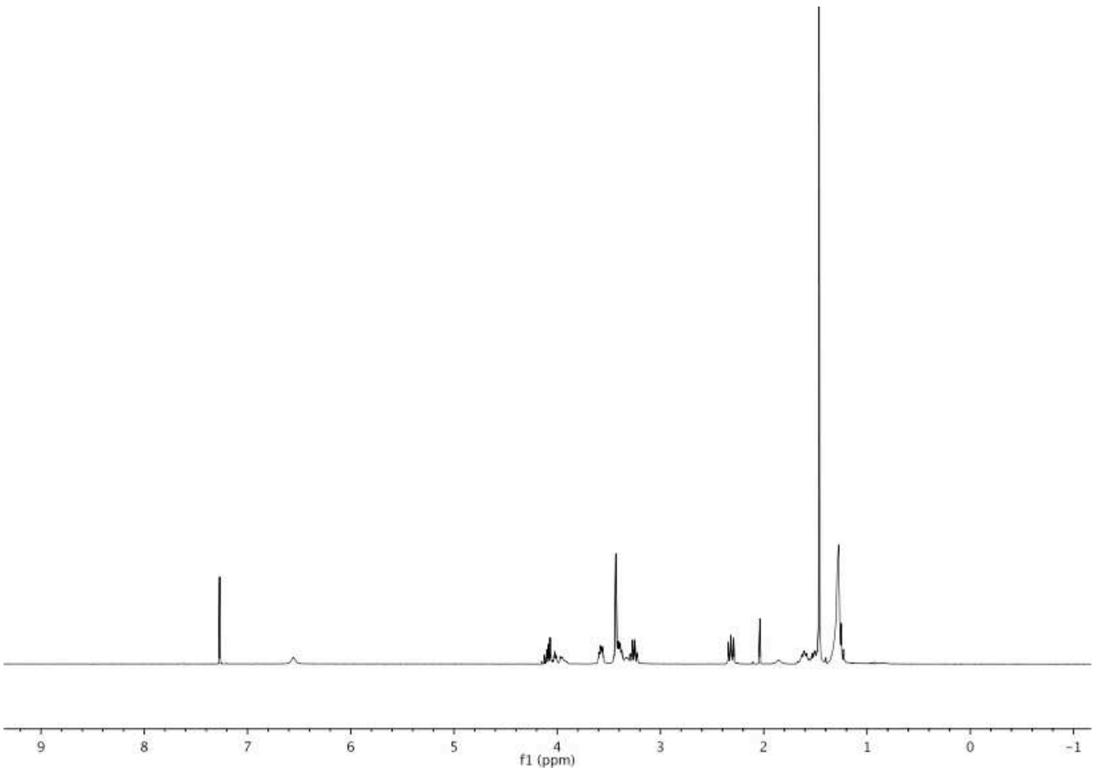

^{1}H NMR of **8_S**

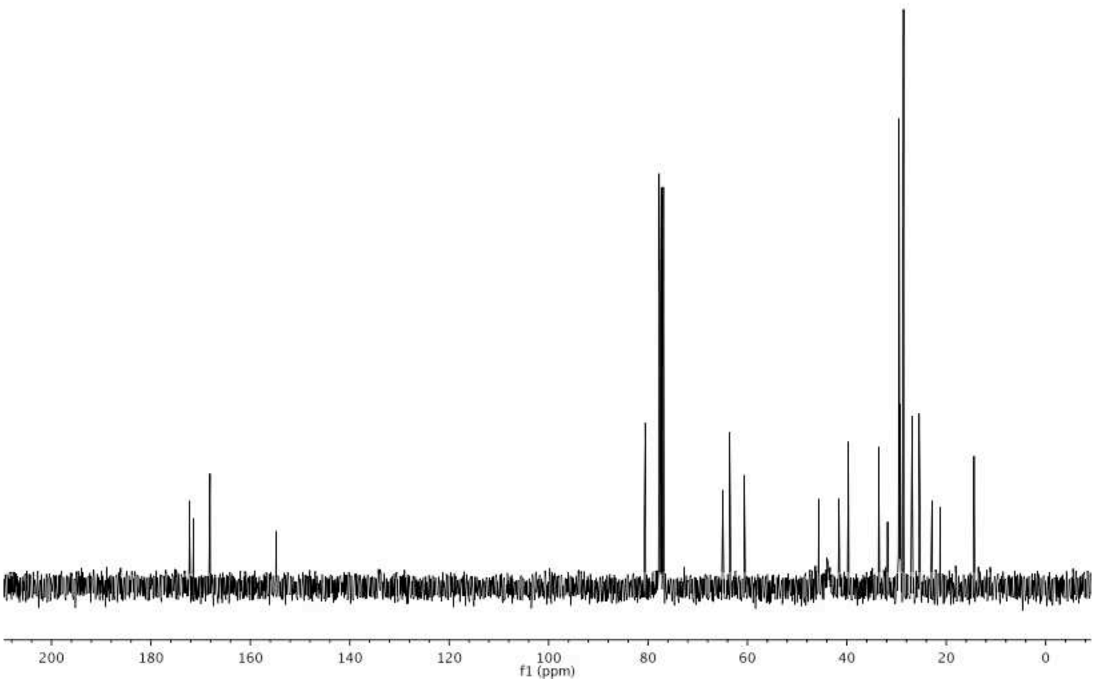

^{13}C NMR of **8_S**

Compound 8_K

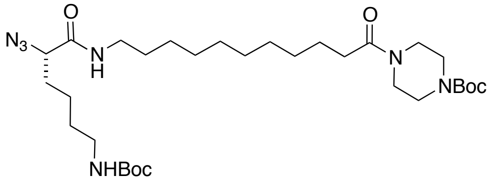

^{1}H NMR (500 MHz, $CDCl_3$) δ 6.68 (t, 1H, J = 9.5 Hz), 4.90 (t, 1H, J = 7.5 Hz), 3.77-3.73 (m, 1H), 3.46-3.42 (m, 2H), 3.31 (s, 4H), 3.31-3.25 (m, 2H), 3.09 (td, 2H, J = 11.5 Hz, J = 22.5 Hz), 2.95 (td, 2H, J = 10.0 Hz, J = 21.0 Hz), 2.19 (t, 2H, J = 12.5 Hz), 1.83-1.61 (m, 2H), 1.50-1.44 (m, 2H), 1.41-1.35 (m, 4H), 1.33 (s, 9H), 1.29 (s, 9H), 1.14 (br, 14H)

^{13}C NMR (125 MHz, $CDCl_3$) δ 171.9, 169.4, 156.2, 154.6, 80.3, 78.9, 64.0, 45.5, 43.7, 41.4, 40.3, 39.6, 33.42, 31.8, 29.8, 29.5(2), 29.4, 29.3(2), 28.5, 28.4, 26.9, 25.4, 22.8

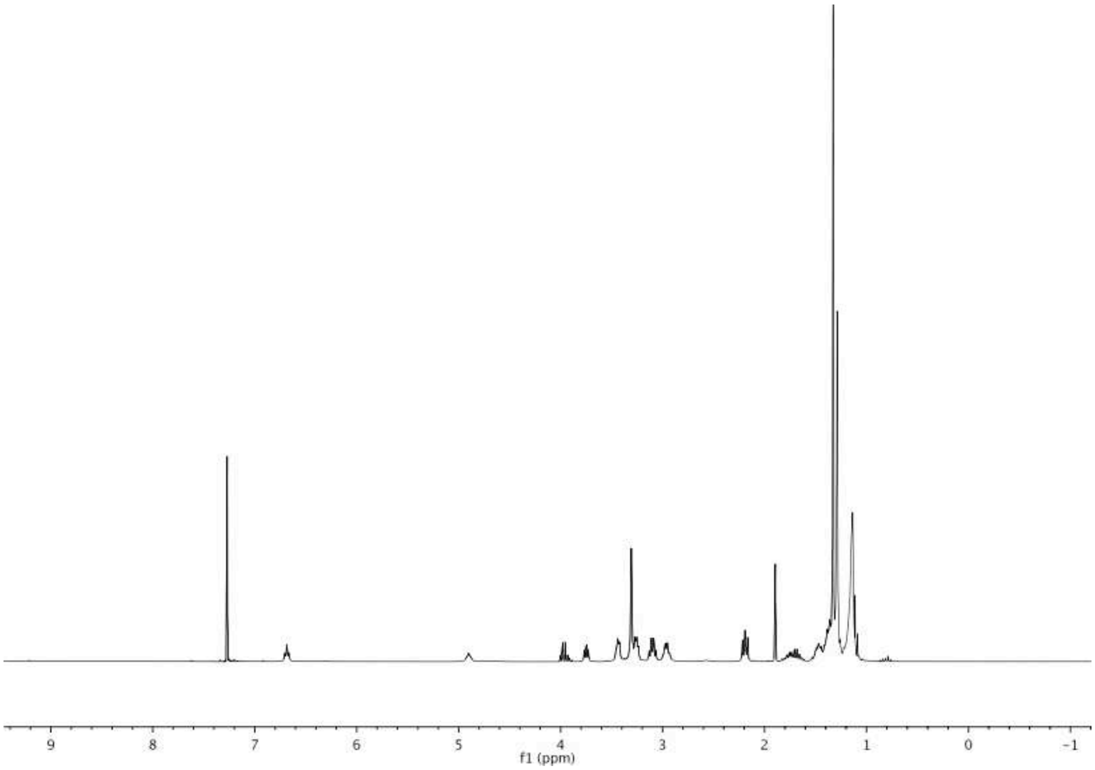

^{1}H NMR of **8_K**

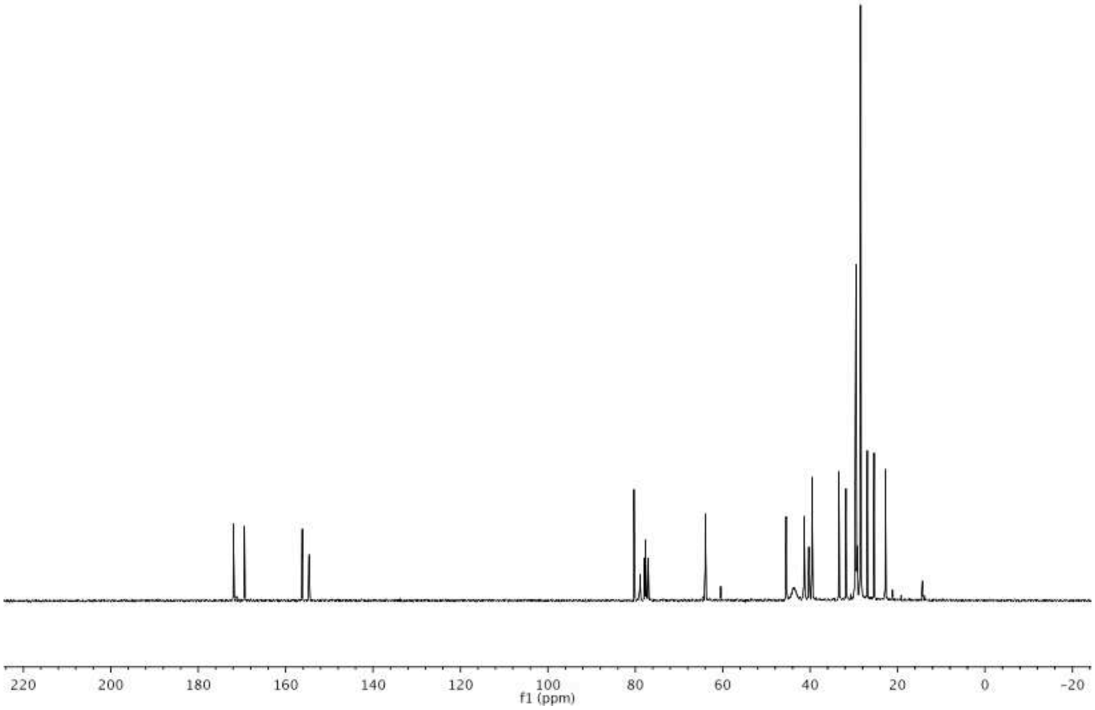

^{13}C NMR of **8_K**

Compound 8_R

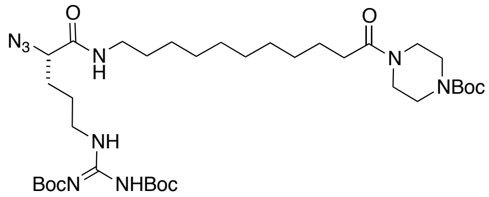

^{1}H NMR (500 MHz, $CDCl_3$) δ 8.39 (t, 1H, J = 9.5 Hz), 6.43 (t, 1H, J = 9.5 Hz), 4.03 (t, 1H, J = 8.5 Hz), 3.61-3.3.57 (m, 2H), 3.44 (s, 4H), 3.41-3.35 (m, 2H), 3.30-3.19 (m, 2H), 2.33 (t, 2H, J = 12.5 Hz), 1.98-1.82 (m, 4H), 1.75-1.67 (m, 2H), 1.62-1.59(m, 2H), 1.50 (s, 18H), 1.47 (s, 9H), 1.28 (br, 12H)

^{13}C NMR (125 MHz, $CDCl_3$) δ N.A.

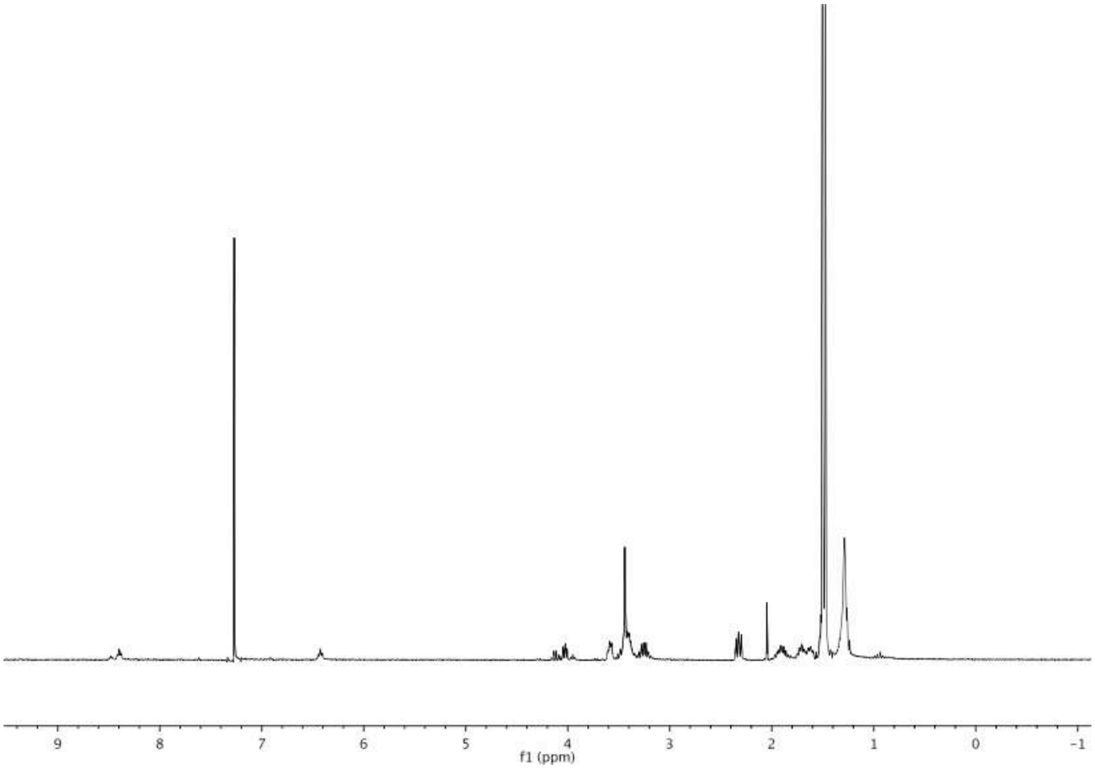

^{1}H NMR of **8_R**

Compound 8_T

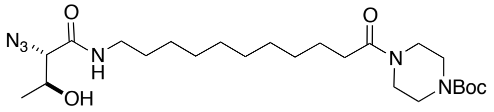

^{1}H NMR (500 MHz, DMSO-d_6) δ 8.13 (t, 1H, J = 5.5 Hz), 5.18 (d, 1H, J = 5.0 Hz), 3.93-9.89 (m, 1H), 3.41-3.38 (m, 5H), 3.30 (br, 2H), 3.24 (br, 2H), 3.11-2.98 (m, 2H), 2.27 (t, 2H, J = 7.5 Hz), 1.48-1.42 (m, 2H), 1.39-1.36 (m, 11H), 1.23 (br, 12H), 1.06 (d, 3H, J = 5.5 Hz)

^{13}C NMR (125 MHz, DMSO-d_6) δ 171.5, 168.6, 154.5, 79.8, 68.4, 67.6, 45.3, 33.0, 29.6(3), 29.5(2), 29.4, 29.3, 28.7, 27.0, 25.4, 20.9

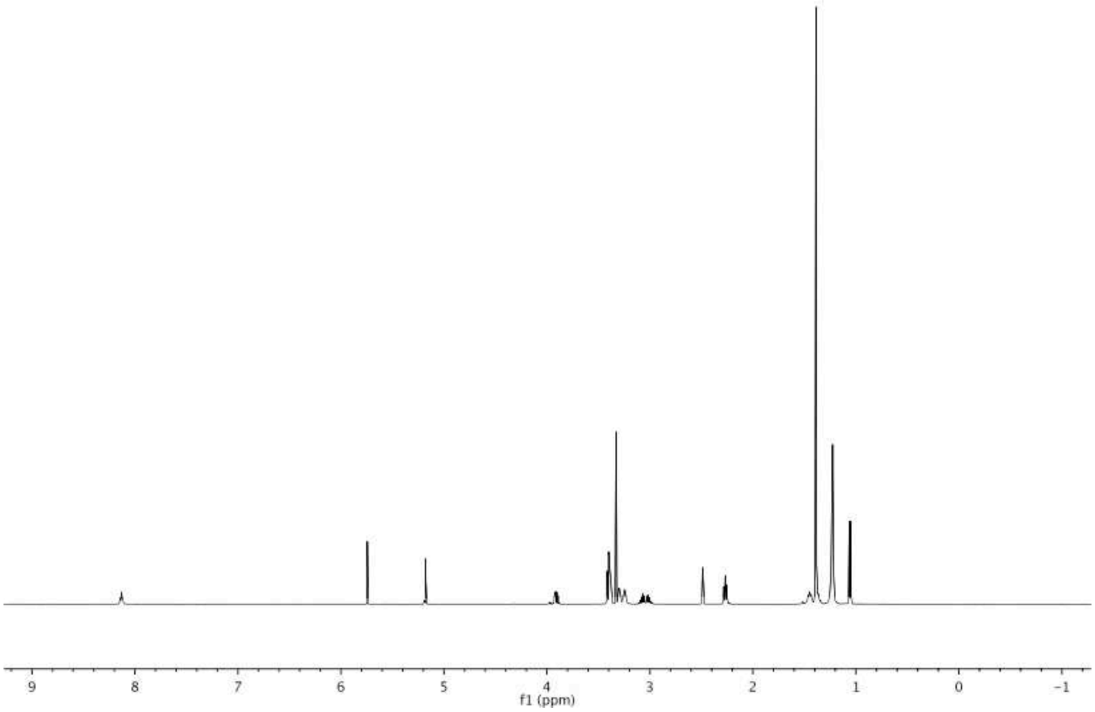

^{1}H NMR of **8_T**

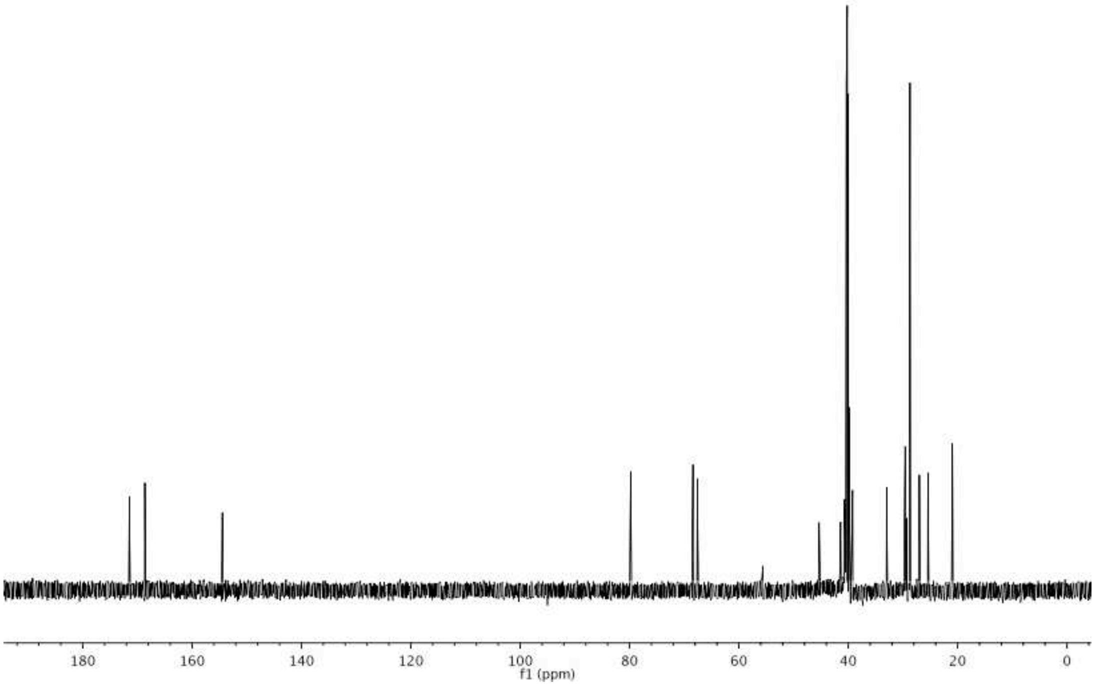

^{13}C NMR of **8_T**

Compound 8_M

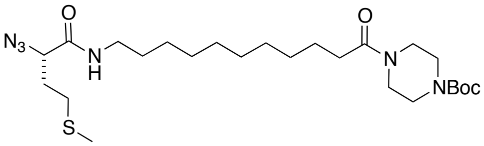

^{1}H NMR (500 MHz, DMSO-d_6) δ 8.22 (t, 1H, J = 5.5 Hz), 3.82 (dd, 1H, J = 6.0 Hz, J = 8.0 Hz), 3.40-3.38 (m, 4H), 3.31-3.29 (m, 2H), 3.24 (br, 2H), 3.11-3.01 (m, 2H), 2.48-2.41 (m, 2H), 2.27 (t, 2H, J = 7.5 Hz), 2.04 (s, 3H), 1.97-1.84 (m, 2H), 1.46-1.43 (m, 2H), 1.39 (s, 11H), 1.23 (br, 12H)

^{13}C NMR (125 MHz, DMSO-d_6) δ 171.5, 169.3, 154.5, 79.8, 61.3, 45.3, 41.4, 33.0, 31.4, 30.1, 29.7, 29.6(2), 29.5(2), 29.3, 28.7, 27.0, 25.4, 15.3

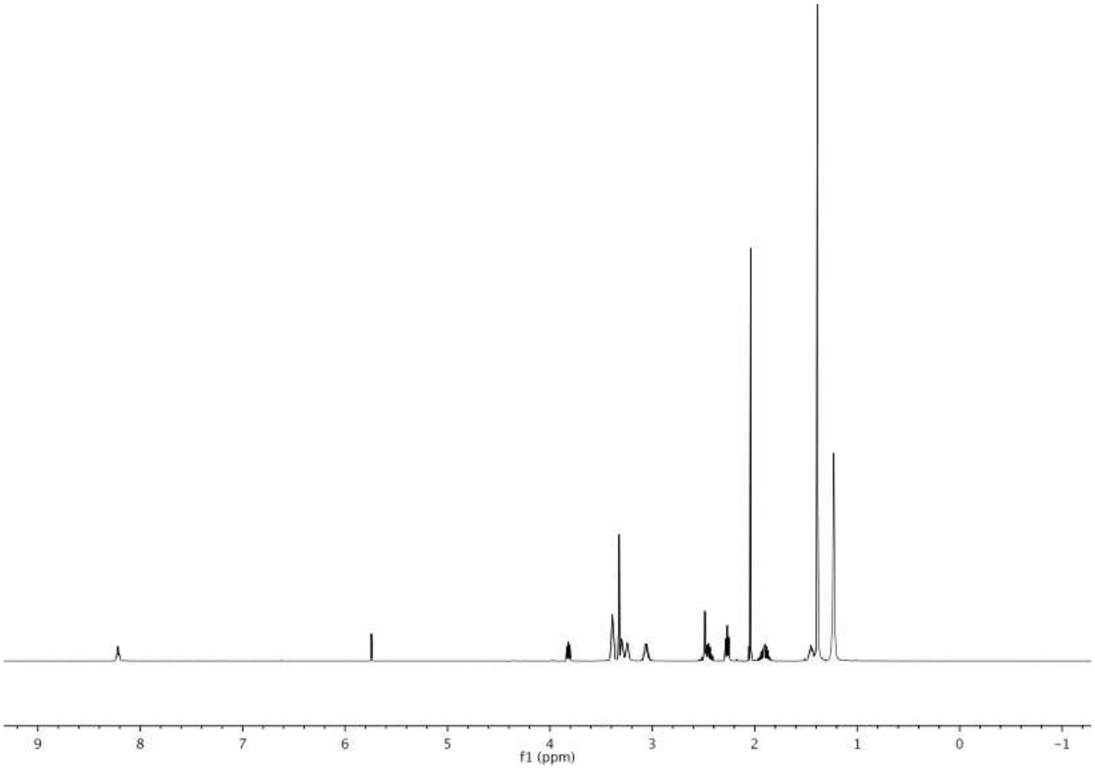

^{1}H NMR of **8_M**

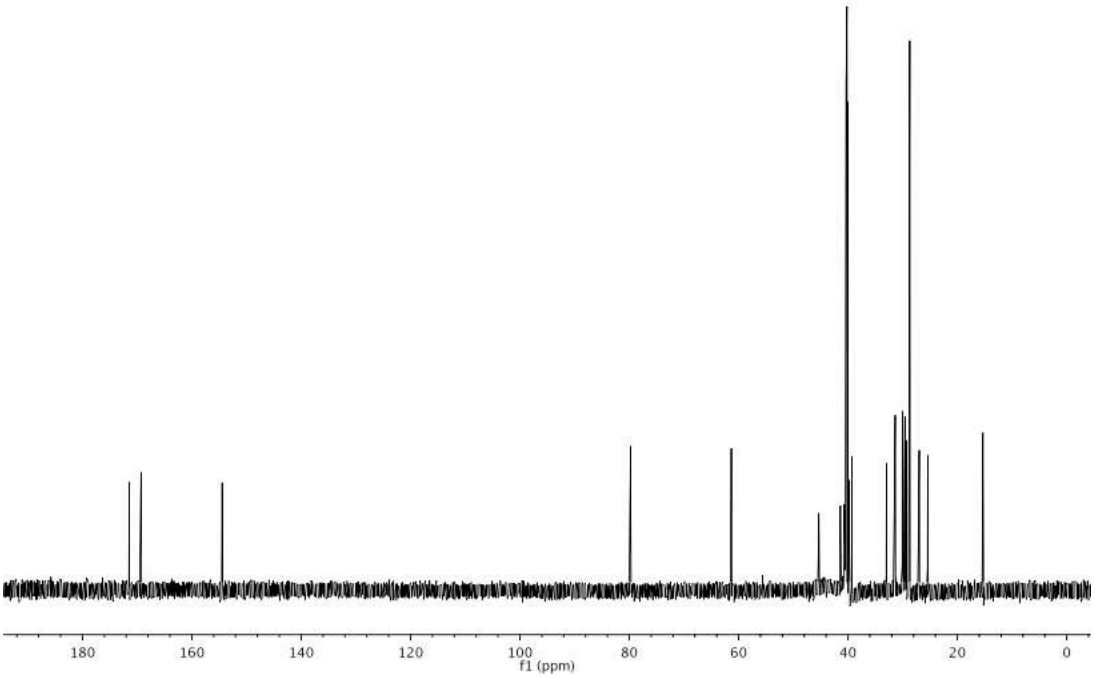

^{13}C NMR of **8_M**

Compound 8_E

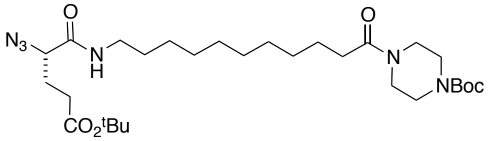

^{1}H NMR (500 MHz, DMSO-d_6) δ 8.19 (t, 1H, J = 5.5 Hz), 3.73 (dd, 1H, J = 6.0 Hz, J = 7.5 Hz), 3.40-3.38 (m, 4H), 3.30 (br, 2H), 3.24 (br, 2H), 3.07-3.03 (m, 2H), 2.27 (t, 2H, J = 7.5 Hz), 2.22 (t, 2H, J = 7.5 Hz), 1.93-1.82 (m, 2H), 1.45 (br, 1H), 1.40-1.37 (m, 21H), 1.26-1.21 (m, 12H)

^{13}C NMR (125 MHz, DMSO-d_6) δ 172.2, 171.9, 171.5, 169.2, 80.6, 79.8, 61.7, 45.3, 42.5, 41.8, 39.2, 33.0, 31.7, 29.7, 29.6,(2), 29.5(2), 29.4, 28.7, 28.4, 27.7, 27.2, 26.9, 25.4

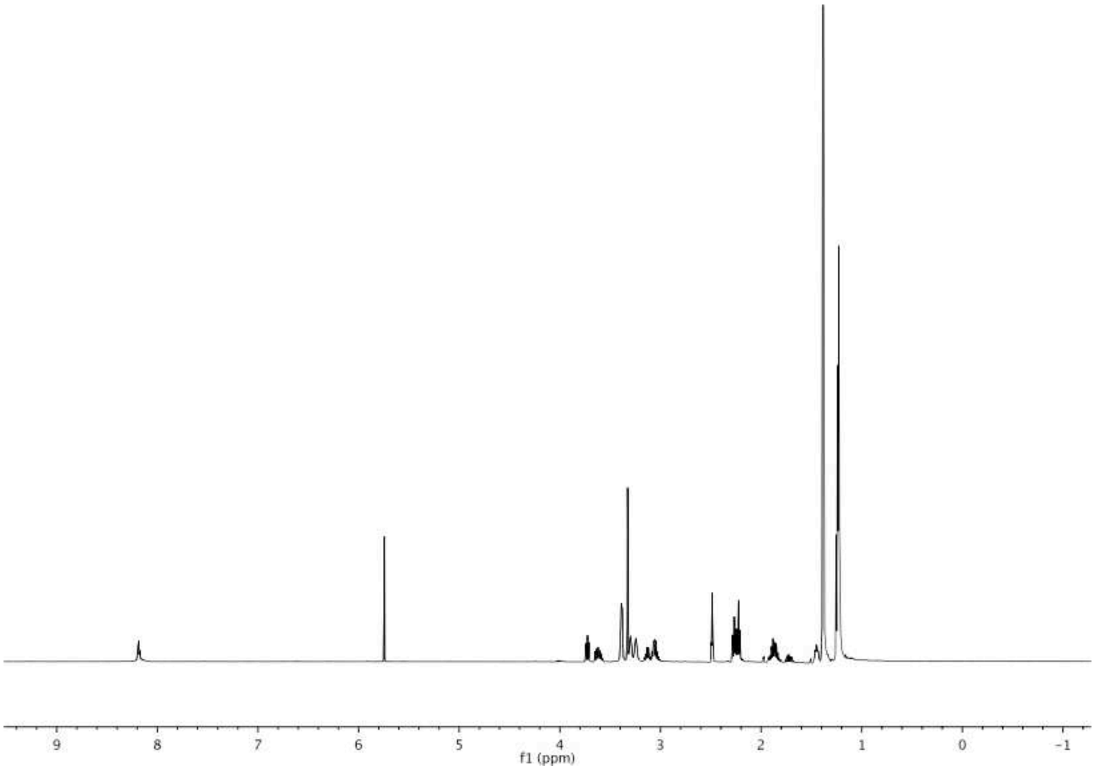

^{1}H NMR of **8_E**

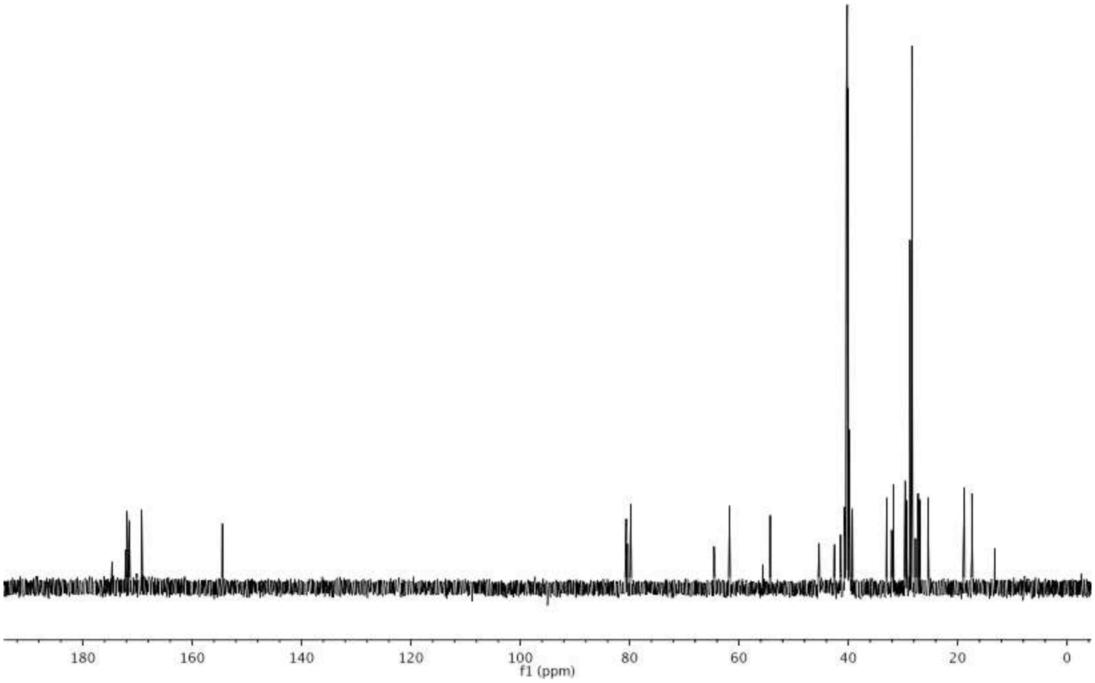

^{13}C NMR of **8_E**

Compound 8_I

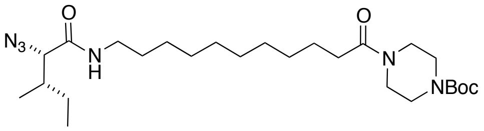

^{1}H NMR (500 MHz, DMSO-d_6) δ 8.16 (t, 1H, J = 5.5 Hz), 3.43-3.38 (m, 5H), 3.30 (br, 2H), 3.24 (br, 2H), 3.14-3.08 (m, 1H), 3.04-3.00 (m, 1H), 2.27 (t, 2H, J = 7.5 Hz), 1.86-1.81 (m, 1H), 1.53-1.42 (m, 3H), 1.39 (s, 12H), 1.23 (br, 11H), 1.19-1.10 (m, 1H), 0.86-0.82 (m, 6H)

^{13}C NMR (125 MHz, DMSO-d_6) δ 171.5, 169.4, 154.5, 79.8, 67.0, 45.3, 41.4, 39.1, 36.3, 33.0, 29.6(3), 29.5(2), 29.3, 28.7, 27.0, 25.4, 25.3, 15.9, 11.2

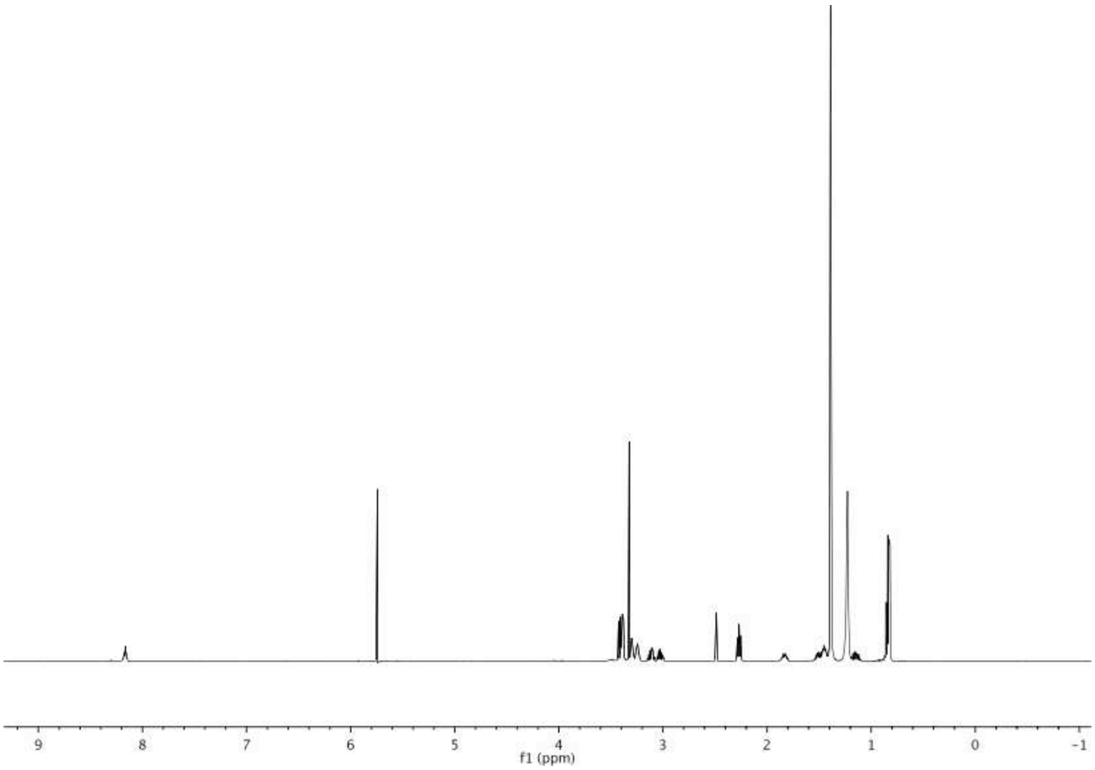

^{1}H NMR of **8_I**

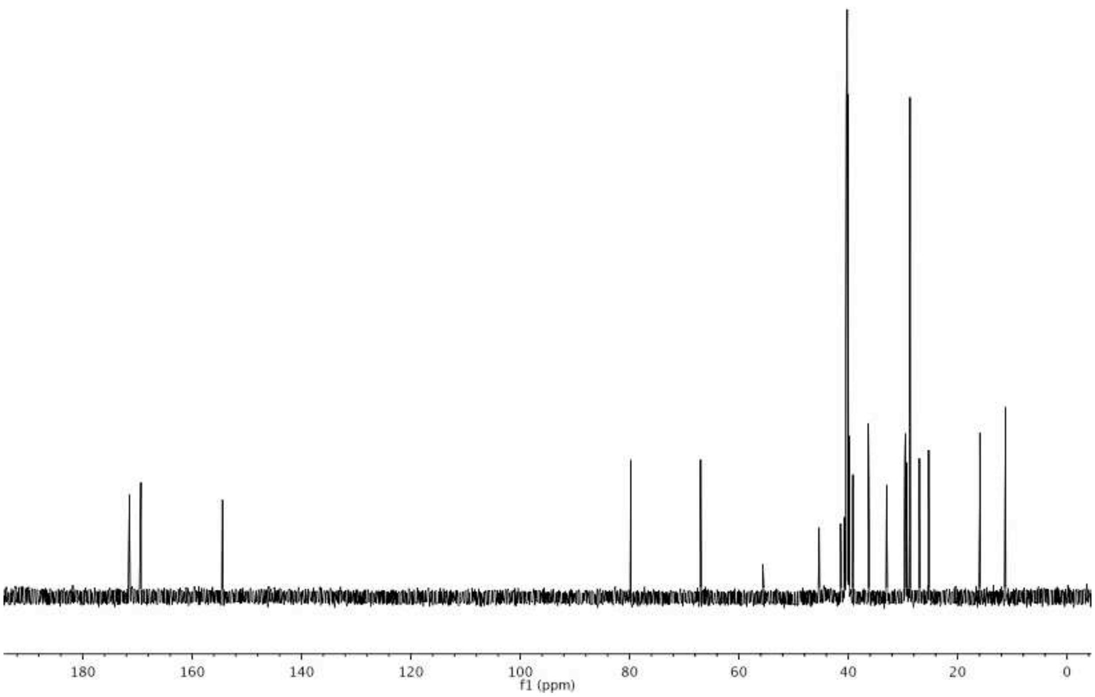

^{13}C NMR of **8_I**

General Procedure for Compounds 9

Alkynes with amino acid side-chains are forms changed from amino acids as the following.

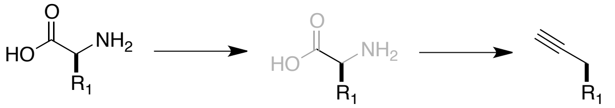

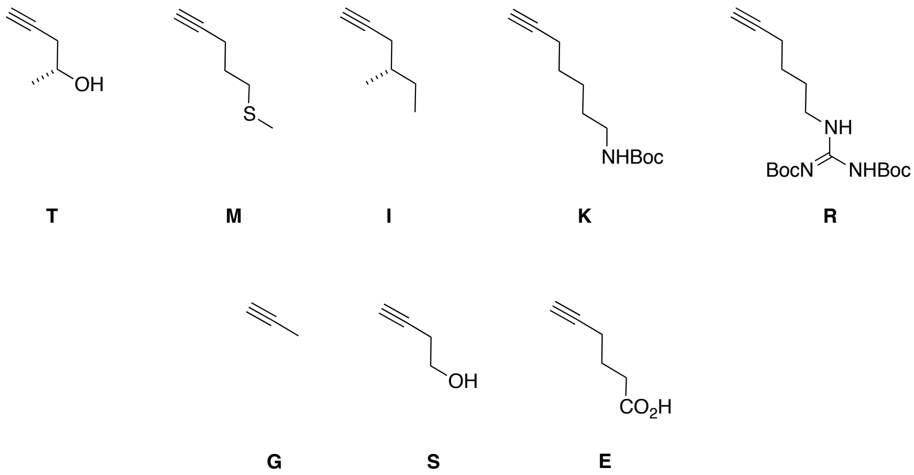

Alkynes corresponding to Gly, Ser, and Glu were obtained from commercial source and used without further purification. Thr[2], Ile[3], Lys[4,5] are known compounds.

Compound 9_M

To a solution of 5-chloro-1-pentyne (1.0 eq.) in DMF (0.2 M) was added sodium thiomethoxide (1.0 eq), and then the mixture was stirred at 50 °C for 4 h.[7] The reaction mixture was diluted with H_2O and extracted with ether (x3). The combined organic phases were dried over Na_2SO_4. After completely removing the solvent, the compound **9_M** was purified by flash chromatography (EtOAc/Hexanes).

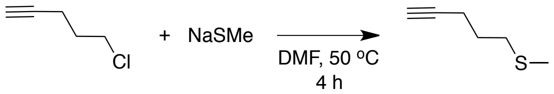

^{1}H NMR (500 MHz, $CDCl_3$) δ 2.60 (t, 2H, J = 11.5 Hz), 2.35-2.29 (td, 2H, J = 4.5 Hz, J = 12.0 Hz), 2.10 (s, 3H), 1.96 (t, 1H, J = 4.5 Hz), 1.86-1.77 (m, 2H)

^{13}C NMR (125 MHz, $CDCl_3$) N.A.

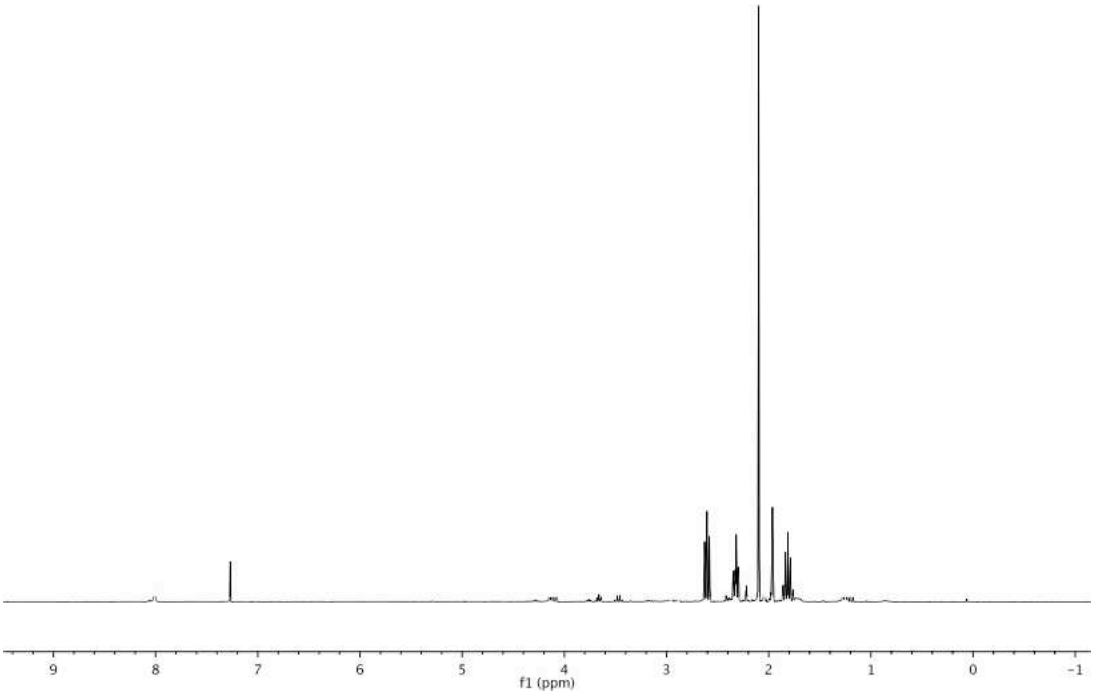

^{1}H NMR of **9_M**

Compound 9_R

To a solution of *N*-Boc-hex5-ynylamine (1.0 eq) in EtOAc:MeOH(10:1, 0.25 M) was slowly added acetyl chloride (7.0 eq) at 0 °C. The mixture was stirred at 0 °C for 2h. After 2h, the mixture was warmed to room temperature, and stirred for 4 h. The solvent was removed and then the residue was dissolved in THF (1.0 M). To the mixture was *tert*-butyl (1*H*-pyrazol-1-yl)methylenedicarbamate (1.0 eq) and Hunig's base (0.6 eq). The resulting suspension was stirred at 25 °C for 48 h. After completely removing the solvent, the compound **9_R** was purified by flash chromatography (EtOAc/Hexanes).[6]

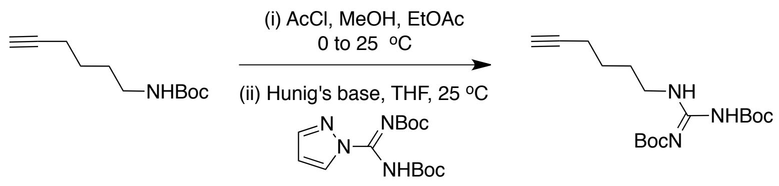

^{1}H NMR (500 MHz, DMSO-d_6) δ 11.50 (s, 1H), 8.30 (t, 1H, J = 5.5 Hz), 3.28 (td, 2H, J = 7.0 Hz, J = 12.5 Hz), 2.76 (t, 1H, J = 2.5 Hz), 2.18 (td, 2H, J = 3.0 Hz, J = 7.5 Hz), 1.58-1.55 (m, 2H), 1.47-1.43 (m, 11H), 1.38 (s, 9H

^{13}C NMR (125 MHz, DMSO-d_6) δ 163.8, 155.9, 152.8, 85.0, 83.5, 78.8, 72.1, 40.3, 28.7, 28.5, 28.3, 26.0, 18.1

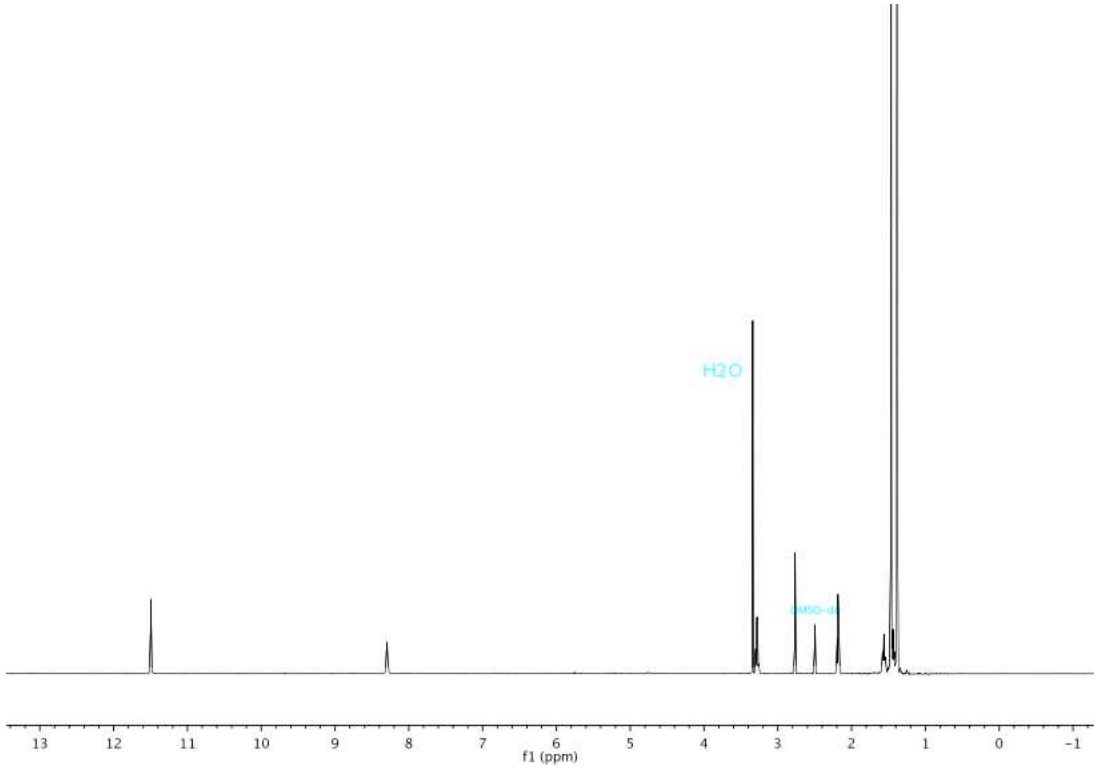

^{1}H NMR of **9_R**

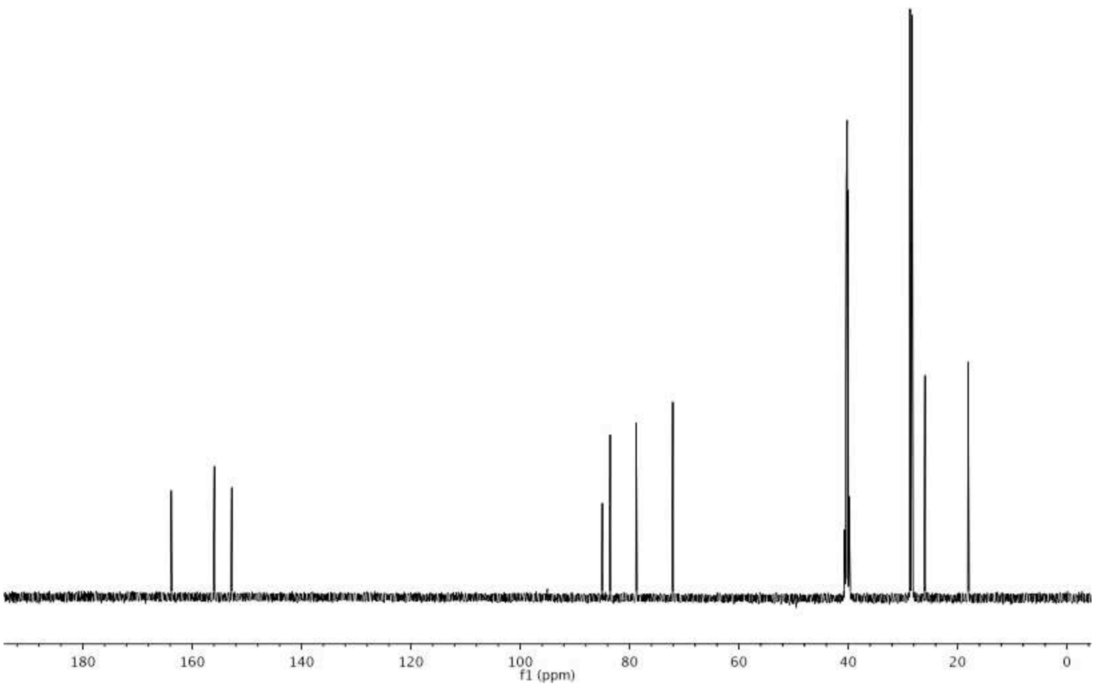

^{13}C NMR of **9_R**

General Procedure for Compounds 10

Compound **8** (1.05 eq) and **9** (1.0 eq.) were dissolved in THF:H_2O (5:1, 0.25 M), and then copper powder (1.0 eq.) and 1*N* $CuSO_4$ (aq., 0.01 eq.) were added. The mixture was stirred at 25 °C for 24 - 48 h. After the reaction, the copper powder was removed by filtration through Celite with CH_2Cl_2 or EtOAc. The filtrate was washed with *sat.* NH_4Cl (aq.): NH_4OH (v:v=9:1), and then brine. The organic layer was dried over Na_2SO_4. After completely removing the solvent, the compound **10** derivatives were purified by flash chromatography (MeOH:CH_2Cl_2).

Scheme S5. Synthesis of Compounds **10**

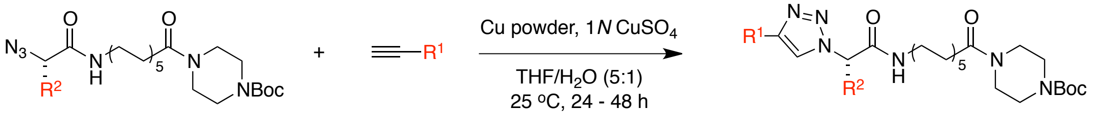

8 **9** **10**, 60-86%

R^{1}R^2 = IK, EK, GK, MS, SK, IR, TG (*i*+1-*i*+2)
KI, KE, KG, SM, KS, RI, GT (*i*+2-*i*+1)

Compound 10_IK

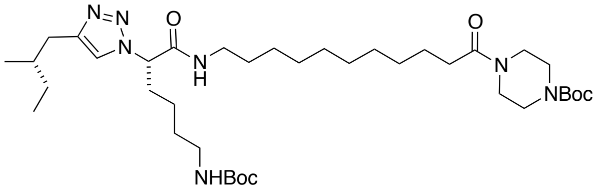

^{1}H NMR (500 MHz, $CDCl_3$) δ 7.51 (s, 1H), 6.74 (br, 1H), 5.09 (dd, 1H, J = 6.0 Hz, J = 9.0 Hz), 4.63 (s, 1H), 3.58-3.56 (m, 2H), 3.42 (s, 4H), 3.39-3.37 (m, 2H), 3.18 (td, 3H, J = 7.0 Hz, J = 13.0 Hz), 3.07-3.02 (m, 2H),

2.70 (dd, 1H, J = 6.0 Hz, J = 14.0 Hz), 2.51 (dd, 1H, J = 7.5 Hz, J = 13.0 Hz), 2.30 (t, 2H, J = 8.0 Hz), 2.27-2.23 (m, 1H), 2.13-2.09 (m, 2H), 1.75-1.73 (m, 1H), 1.61-1.56 (m, 3H), 1.52-1.46 (m, 2H), 1.45 (s, 9H), 1.44-1.41 (br, 11H), 1.32-1.15 (m, 14H), 0.89 (t, 3H, J = 7.5 Hz), 0.86 (d, 3H, J = 6.0 Hz)

^{13}C NMR (125 MHz, $CDCl_3$) δ N.A.

MS (MALDI, m/z) calcd for $C_{38}H_{70}N_7O_6$ $(M+H)^+$ 770.54, found 770.56

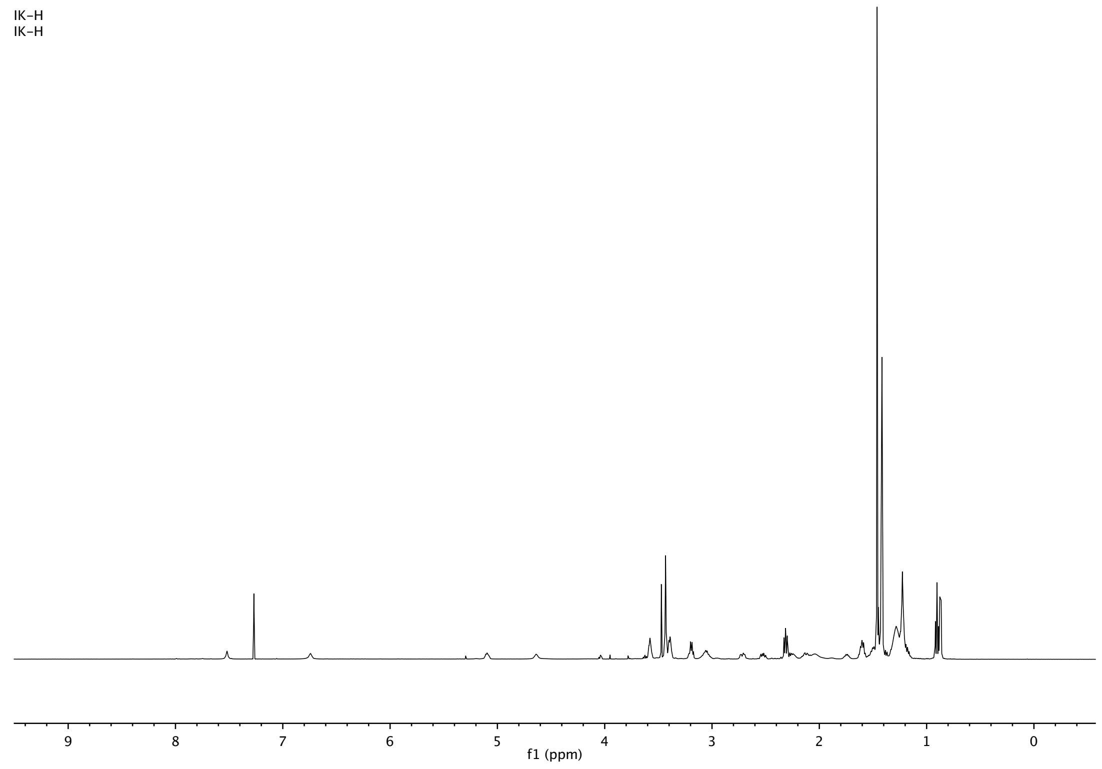

^{1}H NMR of **10_IK**

Compound 10_EK

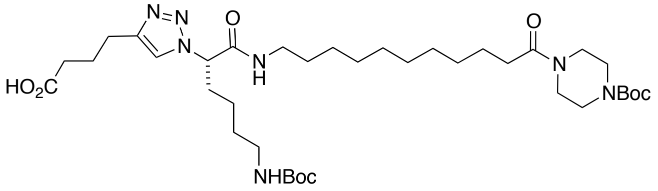

^{1}H NMR (500 MHz, $CDCl_3$) δ 7.68 (s, 1H), 7.02 (s, 1H), 5.19 (dd, 1H, J = 6.5 Hz, J = 9.0 Hz), 4.77 (s, 1H), 3.59-3.57 (m, 2H), 3.43 (br, 4H), 3.38-3.36 (m, 2H), 3.18 (td, 3H, J = 6.0 Hz, J = 13.0 Hz), 3.03 (br, 2H), 2.80 (t, 2H, 7.5 Hz), 2.40-2.36 (m, 2H), 2.31 (t, 2H, J = 7.5 Hz), 2.03-2.00 (m, 2H), 1.59-1.56 (m, 2H), 1.52-1.45 (m, 2H), 1.44 (s, 9H), 1.42 (br, 11H), 1.26-1.19 (m, 16H)

^{13}C NMR (125 MHz, $CDCl_3$) δ 176.1, 172.6, 168.5, 156.6, 155.0, 147.5, 121.6, 80.6, 79.5, 64.5, 45.7, 41.6, 40.2, 40.0, 33.6, 33.5, 32.8, 30.0, 29.6(2), 29.5, 29.3, 28.6(2), 27.0, 25.5, 25.0, 24.5, 23.1

MS (MALDI, m/z) calcd for $C_{37}H_{66}N_7O_8$ $(M+H)^+$ 736.50, found 736.48

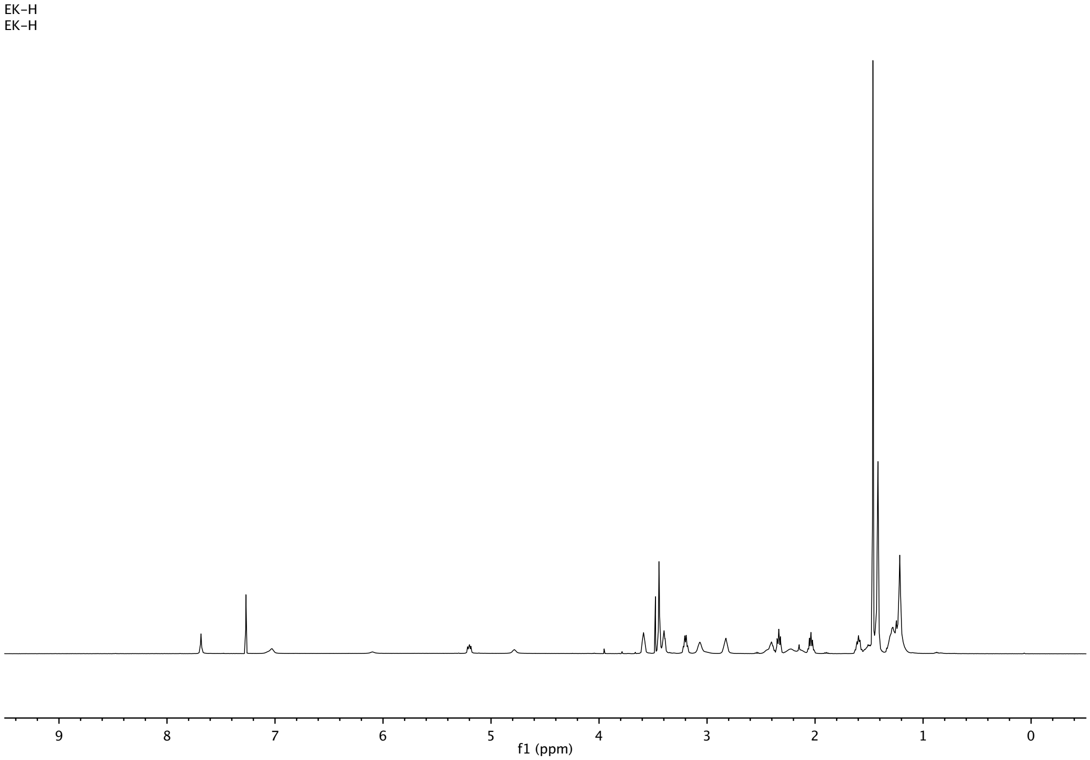

^{1}H NMR of **10_EK**

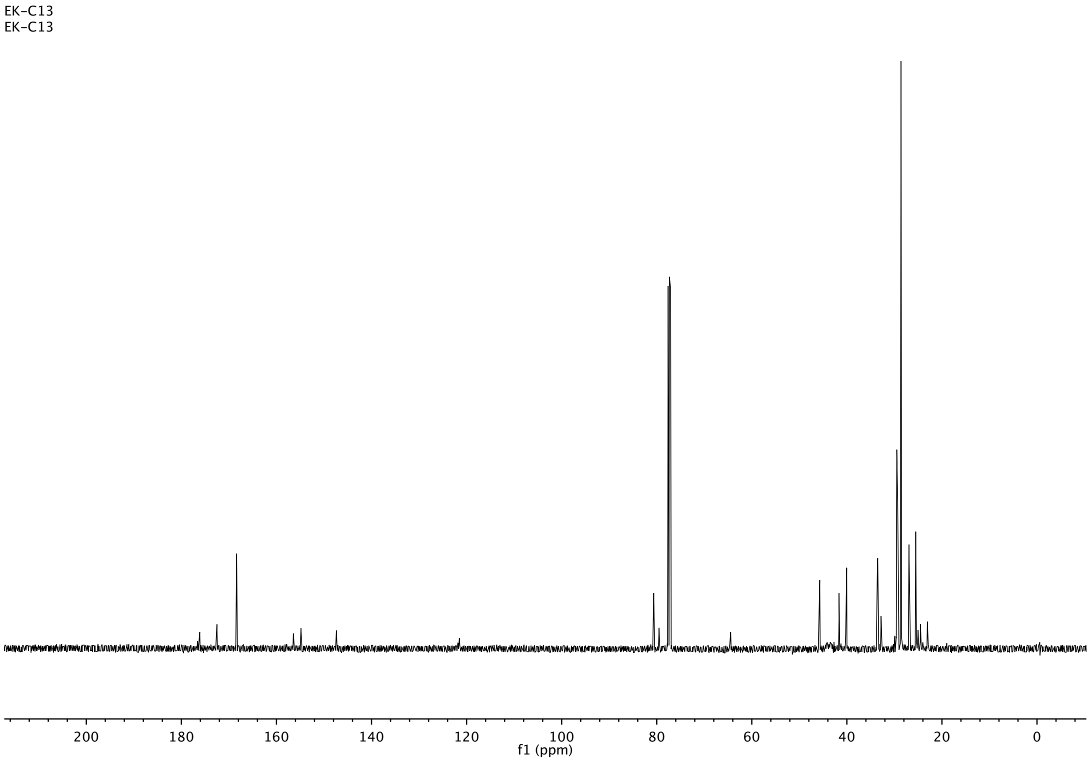

^{13}C NMR of **10_EK**

Compound 10_GK

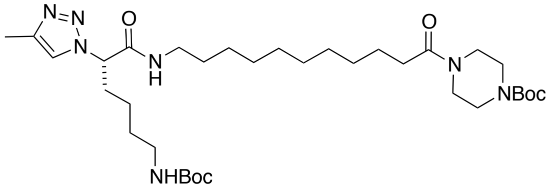

^{1}H NMR (500 MHz, $CDCl_3$) δ 7.51 (s, 1H), 6.71 (br, 1H), 5.06 (dd, 1H, J = 10.0 Hz, J = 15.5 Hz), 4.63 (br, 1H), 3.58-3.55 (m, 2H), 3.42 (br, 4H), 3.40-3.36 (m, 2H), 3.18 (td, 2H, J = 11.0 Hz, J = 22.0 Hz), 3.06-3.01 (m, 2H), 2.34 (s, 3H), 2.33 (t, 2H, J = 3.5 Hz), 2.78-1.97 (m, 2H), 1.16-1.56 (m, 2H), 1.54-1.47 (m, 2H), 1.44 (s, 9H), 1.43 (s, 11H), 1.30-1.18 (m, 14H)

^{13}C NMR (125 MHz, $CDCl_3$) δ 172.3, 168.4, 156.3, 154.9, 80.5, 79.4, 64.7, 45.7, 41.6, 40.2, 40.0, 33.6, 32.9, 29.6(3), 29.5(2), 29.4, 29.3, 28.7, 28.6, 27.0, 25.5, 23.2, 11.1 (two carbon peaks on triazole ring are missed, but methyl group peak on the triazole ring is shown in 11.1. In addition, H NMR clearly shows a proton peak (7.51 pm) on the triazole.)

MS (MALDI, m/z) calcd for $C_{34}H_{62}N_7O_6$ $(M+H)^+$ 664.48, found 664.24

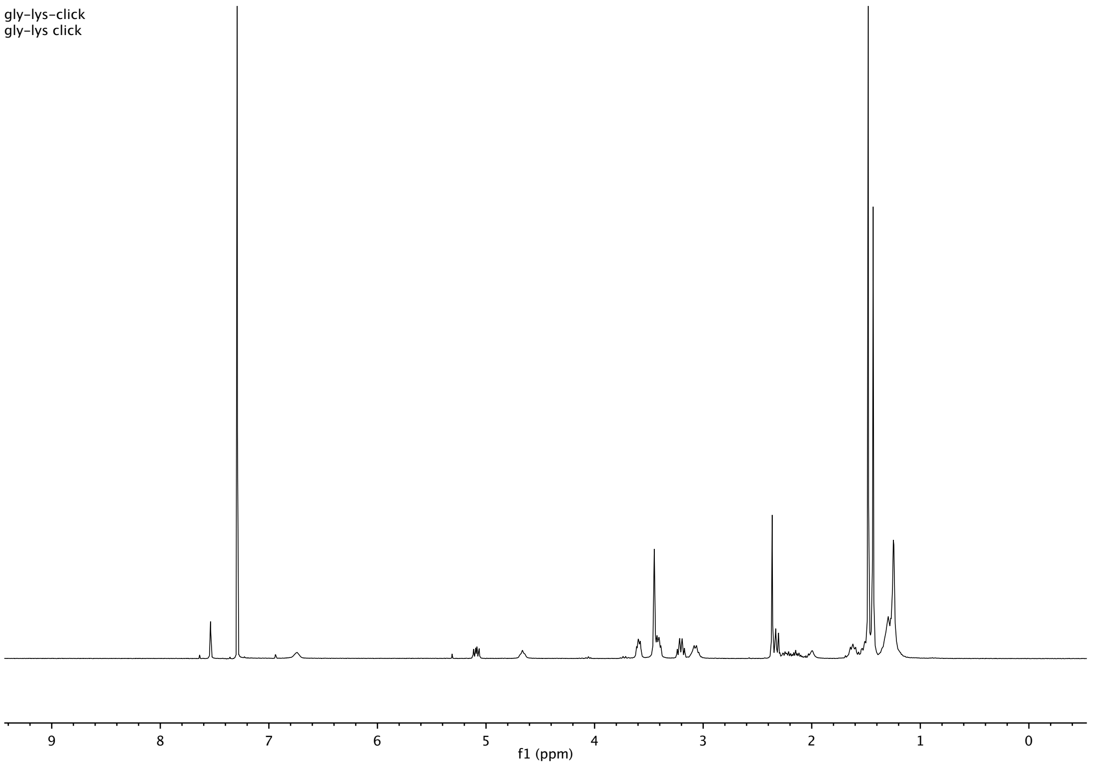

^{1}H NMR of **10_GK**

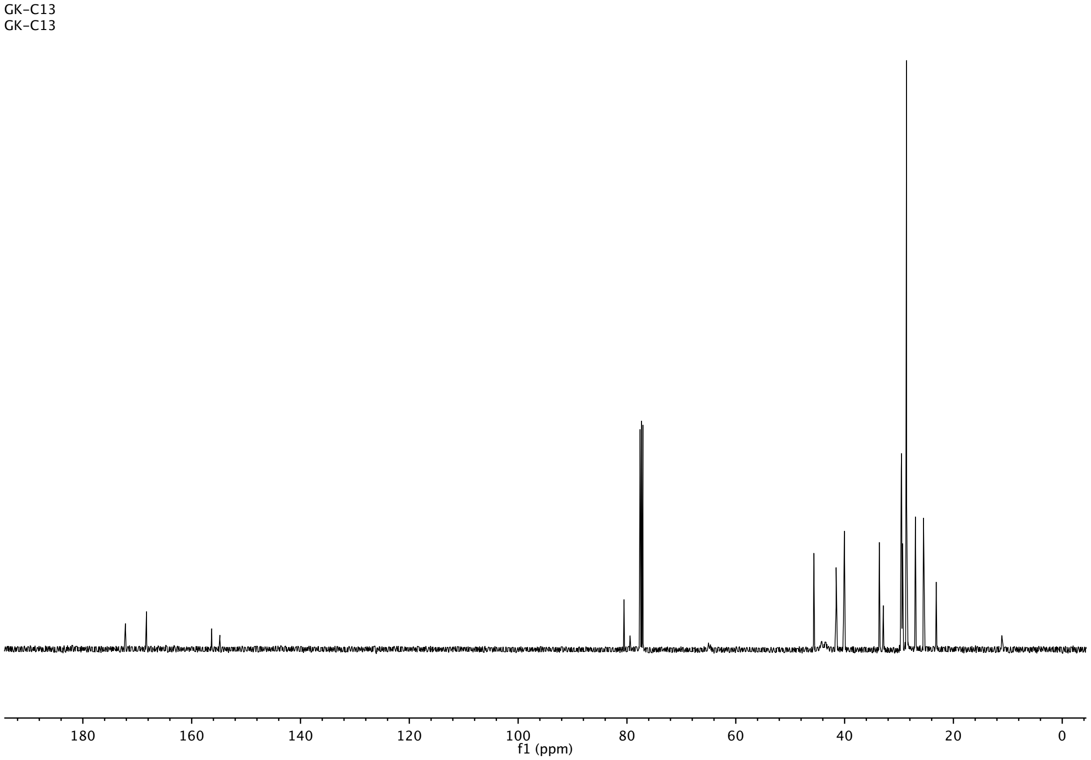

^{13}C NMR of **10_GK**

Compound 10_MS

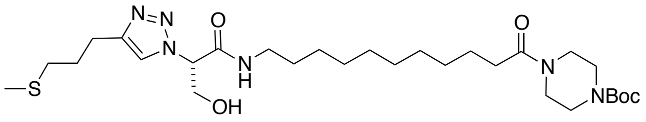

^{1}H NMR (500 MHz, CDCl$_3$) δ 7.82 (s, 1H), 6.98 (br, 1H), 6.37 (dd, 1H, J = 4.0 Hz, J = 5.0 Hz), 4.30 (dd, 1H, J = 5.5 Hz, J = 12.0 Hz), 4.19 (dd, 1H, J = 4.0 Hz, J = 12.0 Hz), 3.58-3.56 (m, 2H), 3.41 (s, 4H), 3.40-3.38 (m, 2H), 3.22 (td, 2H, J = 7.0 Hz, J = 13.0 Hz), 2.87 (t, 2H, J = 6.5 Hz), 2.56 (t, 2H, J = 7.5 Hz), 2.32 (t, 2H, J = 7.5 Hz), 2.09 (s, 3H), 2.03-1.97 (m, 2H), 1.63-1.57 (m, 2H), 1.47 (s, 11H), 1.33-1.24 (m, 12H)

^{13}C NMR (125 MHz, CDCl$_3$) δ 172.2, 167.1, 154.8, 147.6, 122.0, 80.5, 76.9, 65.1, 63.0, 45.6, 41.6, 40.1, 33.8, 33.6, 29.6, 29.5(2), 29.4, 29.3, 29.2, 28.6(2), 26.9, 25.4, 15.6

MS (MALDI, m/z) calcd for $C_{29}H_{53}N_6O_5S$ $(M+H)^+$ 597.38, found 597.38

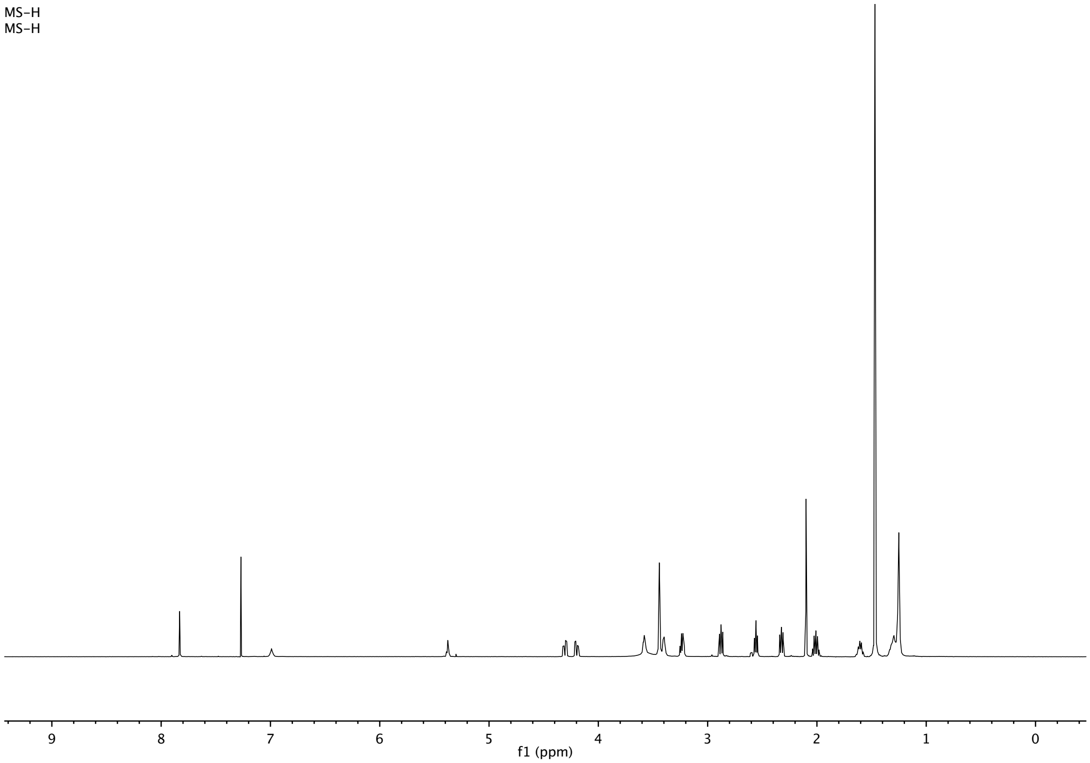

^{1}H NMR of **10_MS**

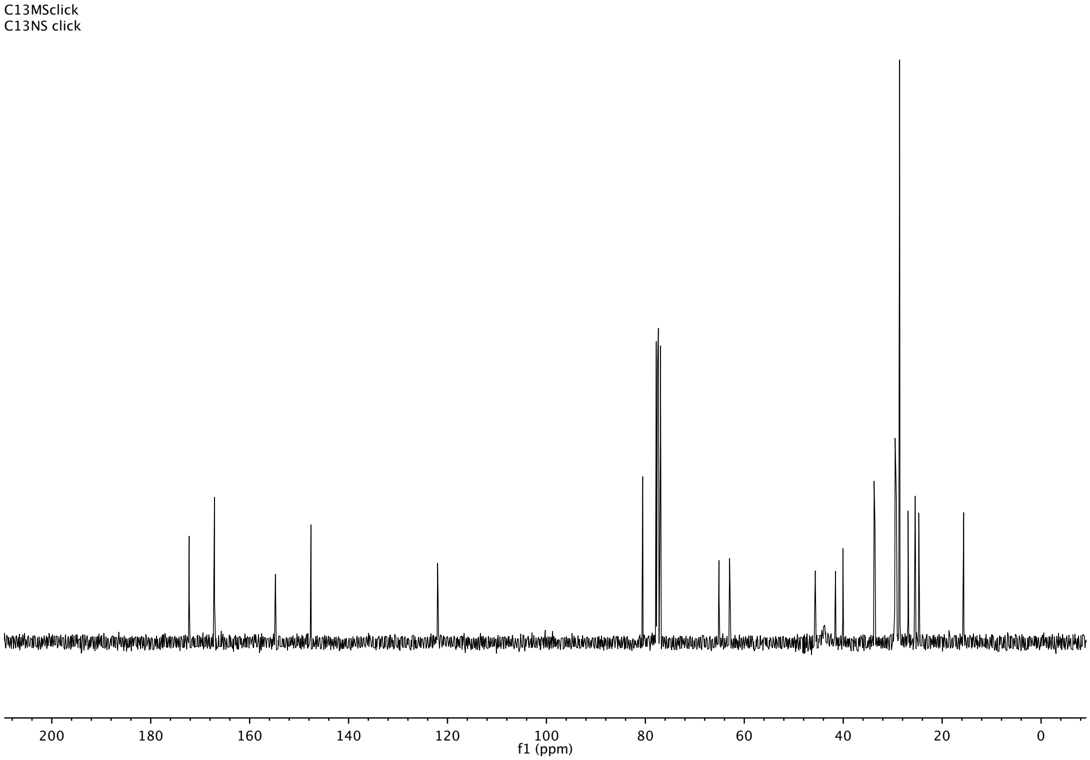

^{13}C NMR of **10_MS**

Compound 10_SK

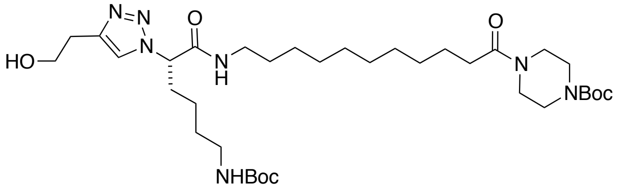

^{1}H NMR (500 MHz, $CDCl_3$) δ 7.74 (s, 1H), 7.10 (br, 1H), 5.18 (dd, 1H, J = 6.5 Hz, J = 9.0 Hz), 4.76 (br, 1H), 3.91 (t, 2H, J = 6.0 Hz), 3.58-3.56 (m, 2H), 3.42 (br, 4H), 3.39-3.37 (m, 2H), 3.18 (td, 2H, J = 6.0 Hz, J = 13.5 Hz), 3.07-3.01 (m, 2H), 2.95 (t, 2H, J = 6.0 Hz), 2.70 (br, 1H), 2.31 (t, 2H, J = 7.5 Hz), 2.25-2.19 (m, 1H), 2.13-2.06 (m, 1H), 1.62-1.56 (m, 2H), 1.50-1.47 (m, 2H), 1.45 (s, 9H), 1.41 (s, 11H), 1.32-1.29 (m, 14H)

^{13}C NMR (125 MHz, $CDCl_3$) δ 172.0, 167.9, 156.2, 154.7, 145.6, 121.8, 80.3, 79.2, 64.3, 61.2, 45.4, 41.3, 39.8, 33.3, 32.4, 29.6, 29.3(2), 29.2(2), 29.1, 29.0(2), 28.7, 28.4, 28.3, 26.6, 25.2, 22.7

MS (MALDI, m/z) calcd for $C_{35}H_{64}N_7O_7$ $(M+H)^+$ 694.49, found 694.43

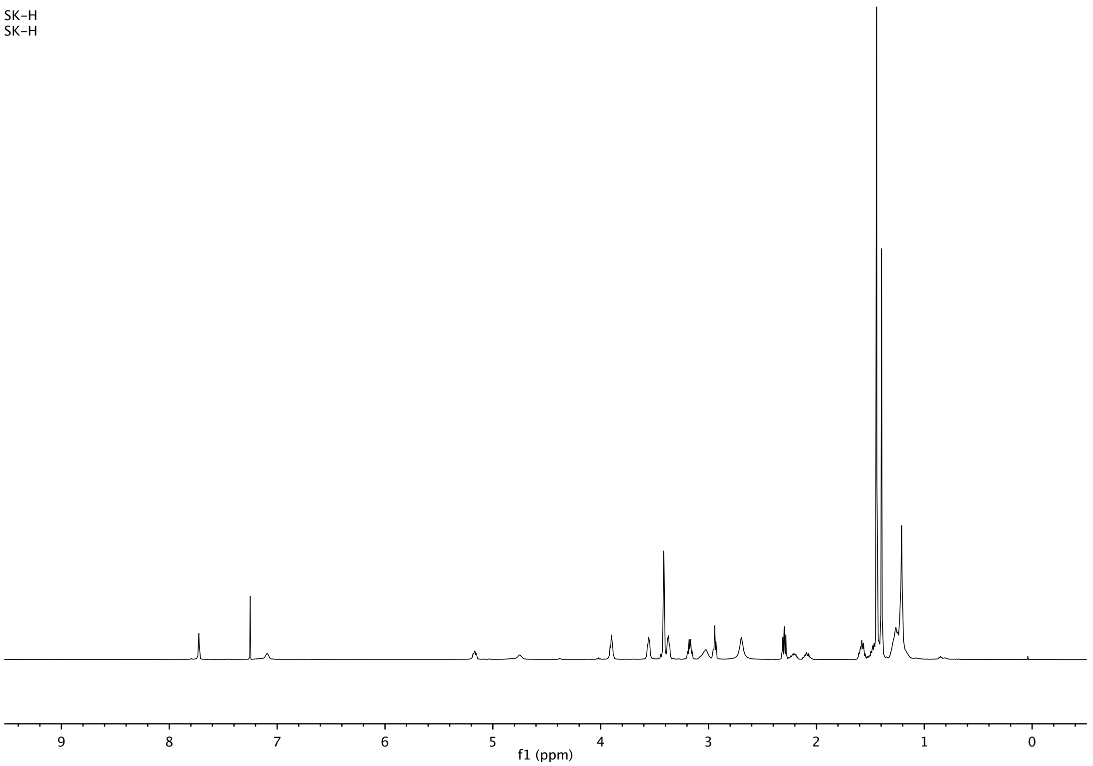

^{1}H NMR of **10_SK**

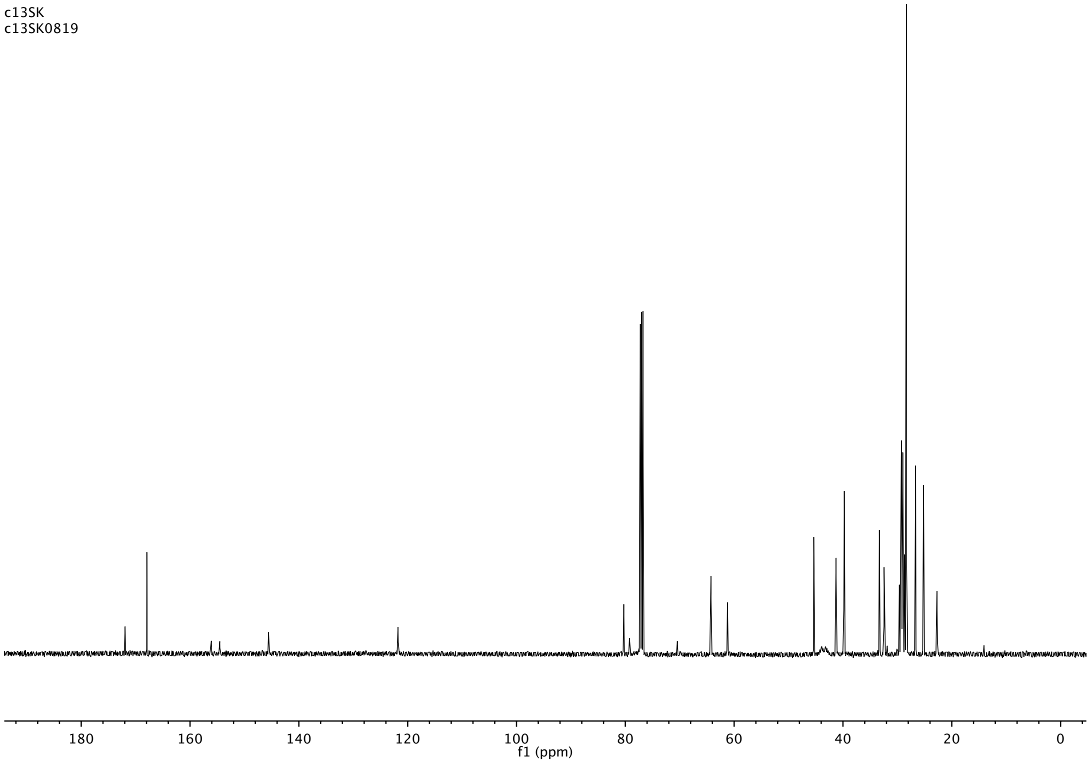

^{13}C NMR of **10_SK**

Compound 10_IR

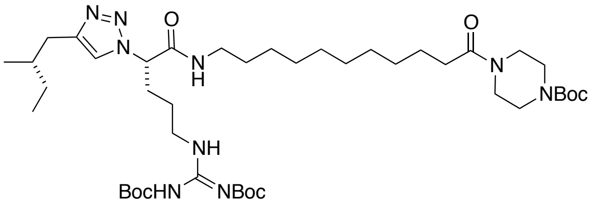

^{1}H NMR (500 MHz, $CDCl_3$) δ 8.36 (t, 1H, J = 9.5 Hz), 7.58 (s, 1H), 6.79 (t, 1H, J = 9.0 Hz) 5.29 (dd, 1H, J = 10.0 Hz, J = 15.0 Hz), 3.60-3.54 (m, 2H), 3.43 (s, 4H), 3.39-3.34 (m, 4H), 3.28-3.12 (m, 2H), 2.71 (dd, 1H, J = 10.0 Hz, J = 24.5 Hz), 2.51 (dd, 1H, J = 13.5 Hz, J = 24.0 Hz), 2.35-2.29 (m, 3H), 2.26-2.22 (m, 1H), 2.17-2.07 (m, 1H), 1.95 (br, 2H), 1.77-1.70 (m, 1H), 1.63-1.51 (m, 3H), 1.49 (s, 9H), 1.48 (s, 8H), 1.46-1.43 (m, 11H), 1.30-1.16 (m, 12H), 0.90 (t, 3H, J = 12.5 Hz), 0.87 (d, 3H, J = 12.5 Hz)

^{13}C NMR (125 MHz, $CDCl_3$) δ 171.8, 168.0, 162.5, 156.2, 154.6, 152.9, 147.3, 121.1, 83.5, 80.2, 79.9, 63.1, 45.3, 41.2, 39.7, 39.4, 34.8, 33.3, 32.6, 30.1, 29.3, 29.2(2). 29.1, 29.0, 28.2, 28.1, 27.9, 26.6, 25.3, 25.2, 18.9, 11.3

MS (MALDI, m/z) calcd for $C_{43}H_{78}N_9O_8$ $(M+H)^+$ 848.60, found 848.75

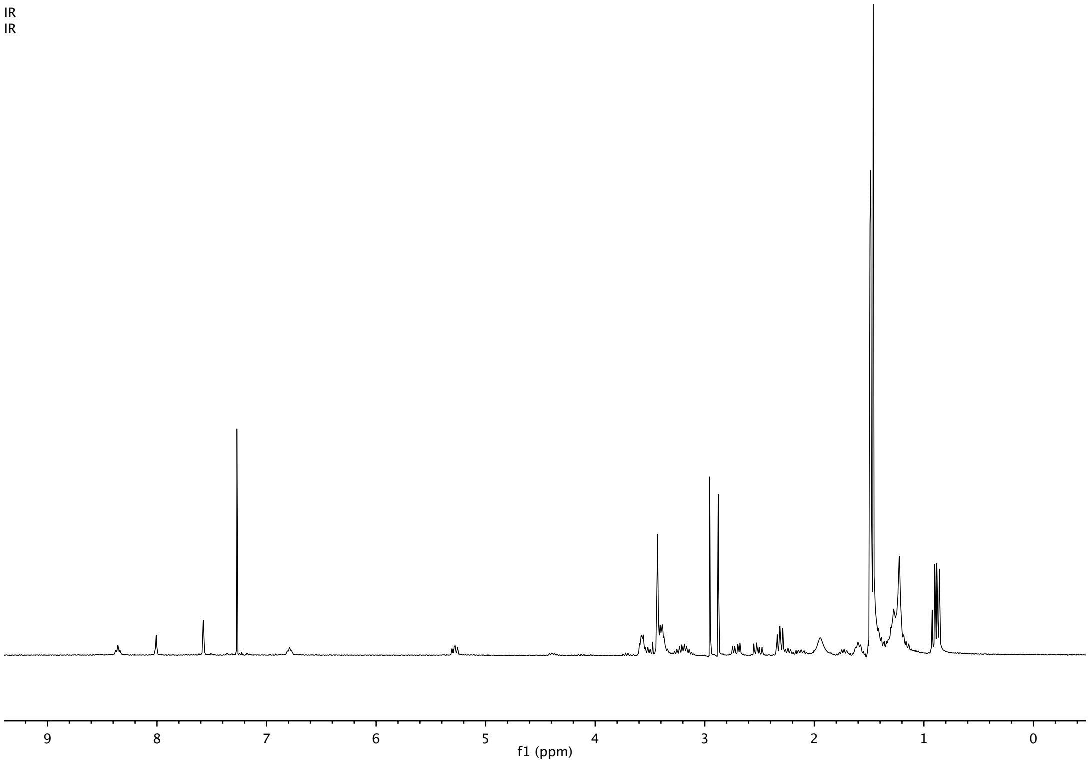

^{1}H NMR of **10_IR**

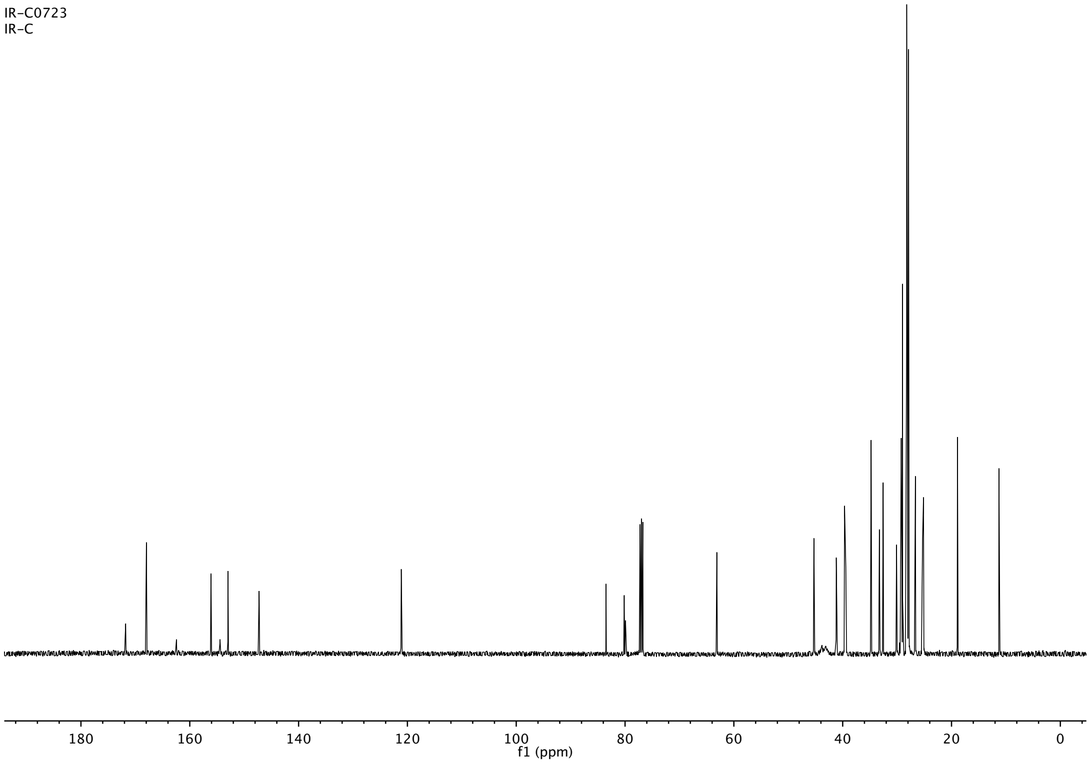

^{13}C NMR of **10_IR**

Compound 10_TG

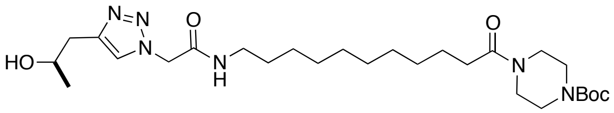

^{1}H NMR (500 MHz, $CDCl_3$) δ 7.56 (s, 1H), 6.30 (br, 1H) 5.00 (s, 1H), 4.17-4.11 (m, 1H), 3.59-3.56 (m, 2H), 3.43 (s, 4H), 3.43-3.37 (m, 2H), 3.21 (td, 2H, J = 11.5 Hz, J = 21.5 Hz), 2.94 (br, 1H) 2.91(dd, 1H, J = 6.5 Hz, J = 25.0 Hz), 2.79 (dd, 1H, J = 13.5 Hz, J = 25.0 Hz), 2.34-2.28 (m, 2H), 1.63-1.53 (m, 2H), 1.45 (s, 11H), 1.30-1.20 (m, 15H)

^{13}C NMR (125 MHz, $CDCl_3$) δ 172.2, 165.5, 161.5, 154.8, 147.5, 123.7, 80.6, 67.2, 53.3, 45.7, 41.6, 40.1, 35.1, 33.6, 29.6, 29.5, 29.4(2), 29.3, 28.7, 26.9, 25.5, 23.2

MS (MALDI, m/z) calcd for $C_{27}H_{49}N_6O_5$ $(M+H)^+$ 537.38, found 537.35

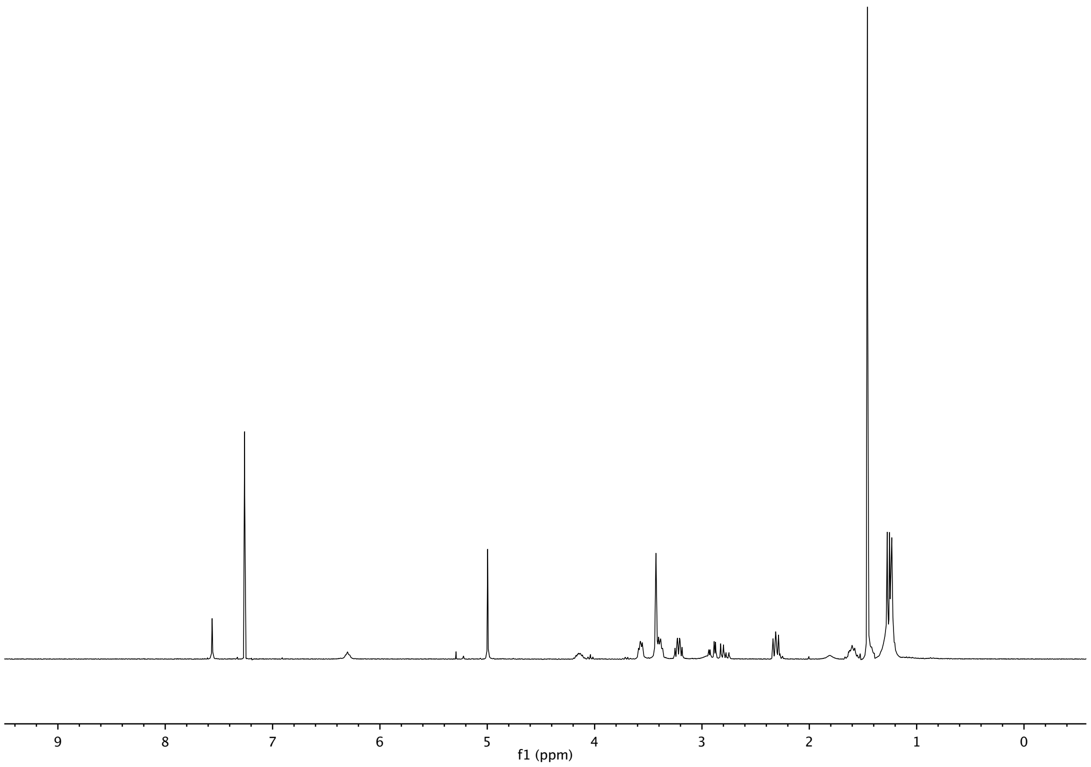

^{1}H NMR of **10_TG**

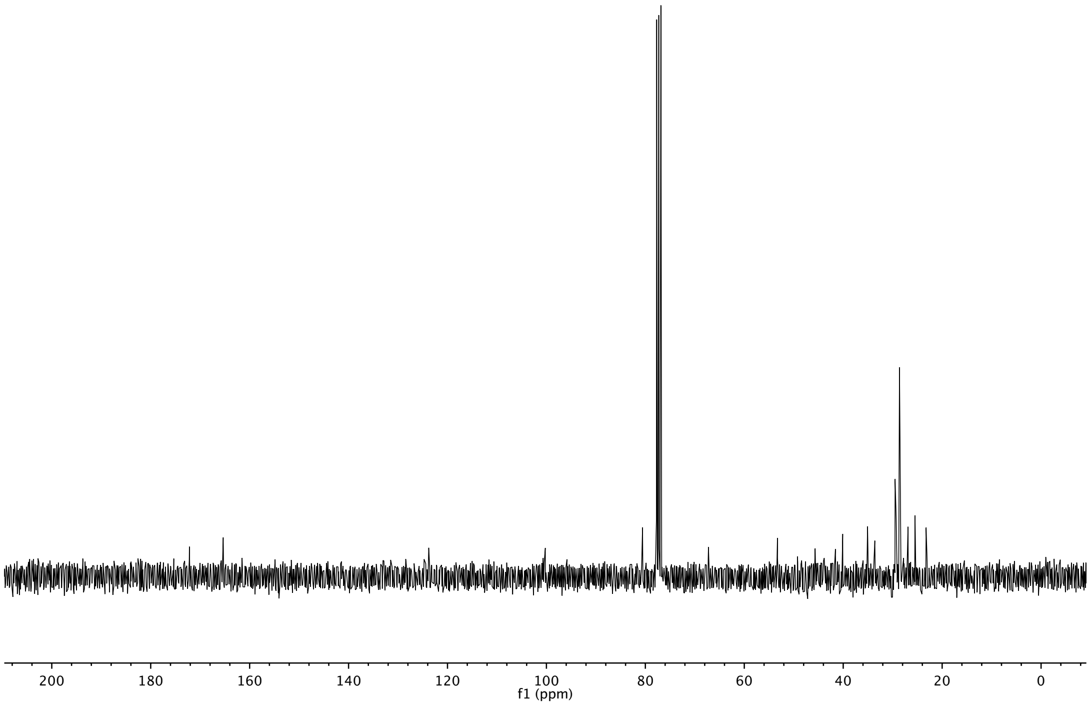

^{13}C NMR of **10_TG**

Compound 10_KI

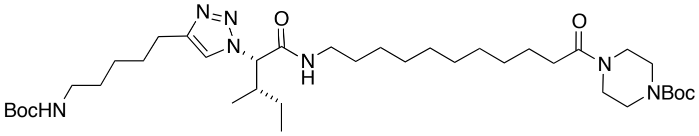

^{1}H NMR (500 MHz, DMSO-d_6) δ 8.55 (t, 1H, J = 5.5 Hz), 7.88 (s, 1H), 6.77 (t, 1H, J = 6.0 Hz), 4.95 (d, 1H, J = 11.0 Hz), 3.41-3.25 (m, 8H), 3.15-2.95 (m, 2H), 2.89 (q, 2H, J = 7.5 Hz), 2.59 (t, 2H, J = 6.0 Hz), 2.29 (t, 2H, J = 6.0 Hz), 2.20 (m, 1H), 1.58 (m, 2H), 1.39 (m, 24H), 1.26 (m, 15H), 0.90 (d, 3H, J = 6.5 Hz), 0.75 (t, 3H, J = 7 Hz)

^{13}C NMR (126 MHz, DMSO-d_6) δ 171.5, 168.1, 156.2, 154.4, 147.6, 120.9, 95.0, 79.8, 77.9, 67.8, 45.31, 41.4, 39.2, 37.2, 33.0, 29.9, 29.6, 29.6, 29.5, 29.3, 29.3, 28.9, 28.7, 26.9, 26.6, 25.7, 25.4, 25.0,15.7, 10.5

MS (MALDI, m/z) calcd for $C_{38}H_{70}N_7O_8$ $(M+H)^+$ 720.54, found 720.58

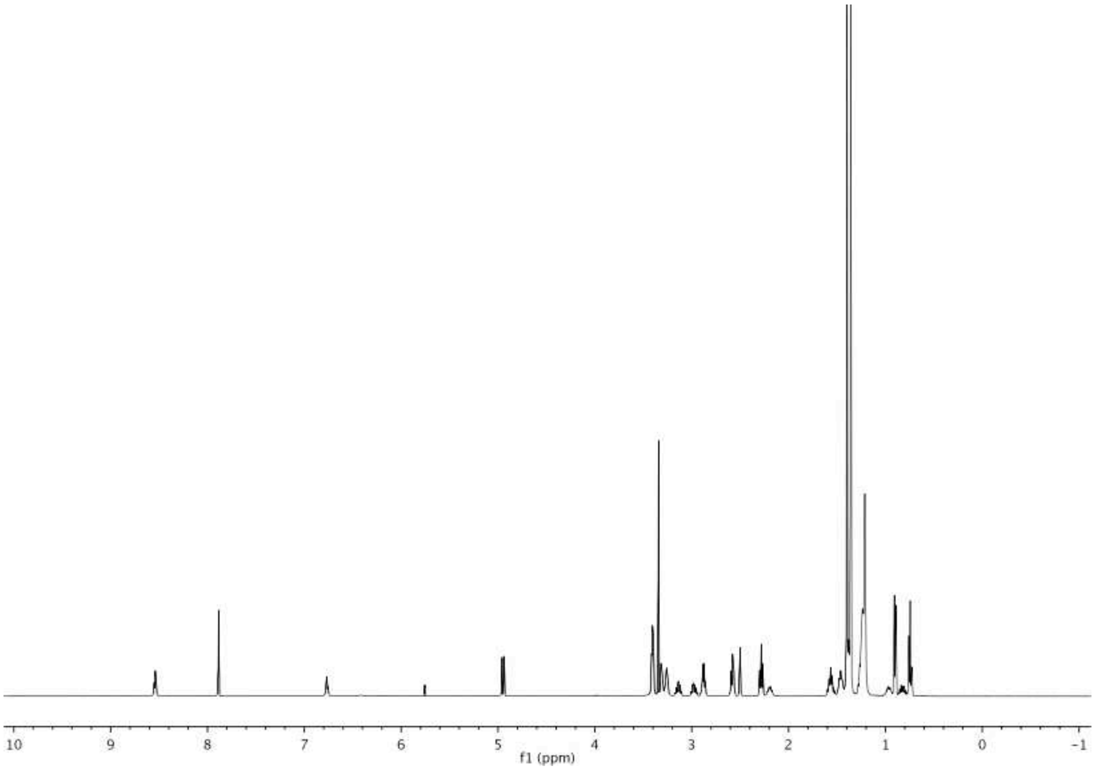

^{1}H NMR of **10_KI**

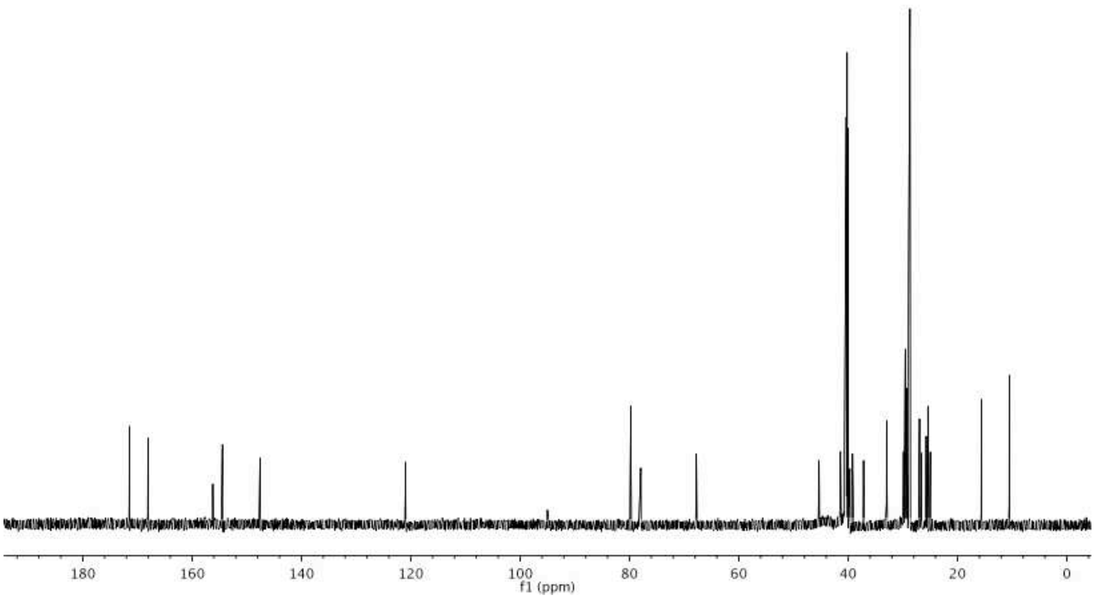

^{13}C NMR of **10_KI**

Compound 10_KE

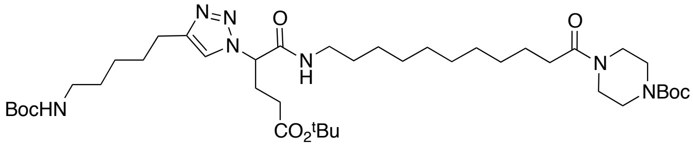

^{1}H NMR (500 MHz, DMSO-d_6) δ 8.42 (t, 1H, J = 5.5 Hz), 7.88 (s, 1H), 6.78 (t, 1H, J = 6.0 Hz), 5.19 (t, 1H, J = 8.0 Hz), 3.40-3.25 (m, 8H), 3.11-2.99 (m, 2H), 2.90 (q, 2H, J = 6.5 Hz), 2.59 (t, 2H, J = 6.0 Hz), 2.29-2.04 (m, 6H), 1.58 (m, 2H), 1.39 (m, 37H), 1.21 (m, 14H)

^{13}C NMR (126 MHz, DMSO-d_6) δ 171.5(2), 167.8, 156.2, 154.4, 147.6, 121.4, 80.7, 79.8, 77.9, 62.5, 45.3, 41.4, 39.3, 33.0, 31.5, 29.9, 29.7, 29.6(2), 29.5, 29.3(2), 28.9, 28.7, 28.4, 28.0, 26.9, 26.6, 25.7, 25.4

MS (MALDI, m/z) calcd for $C_{41}H_{74}N_7O_8$ $(M+H)^+$ 792.06, found 792.64

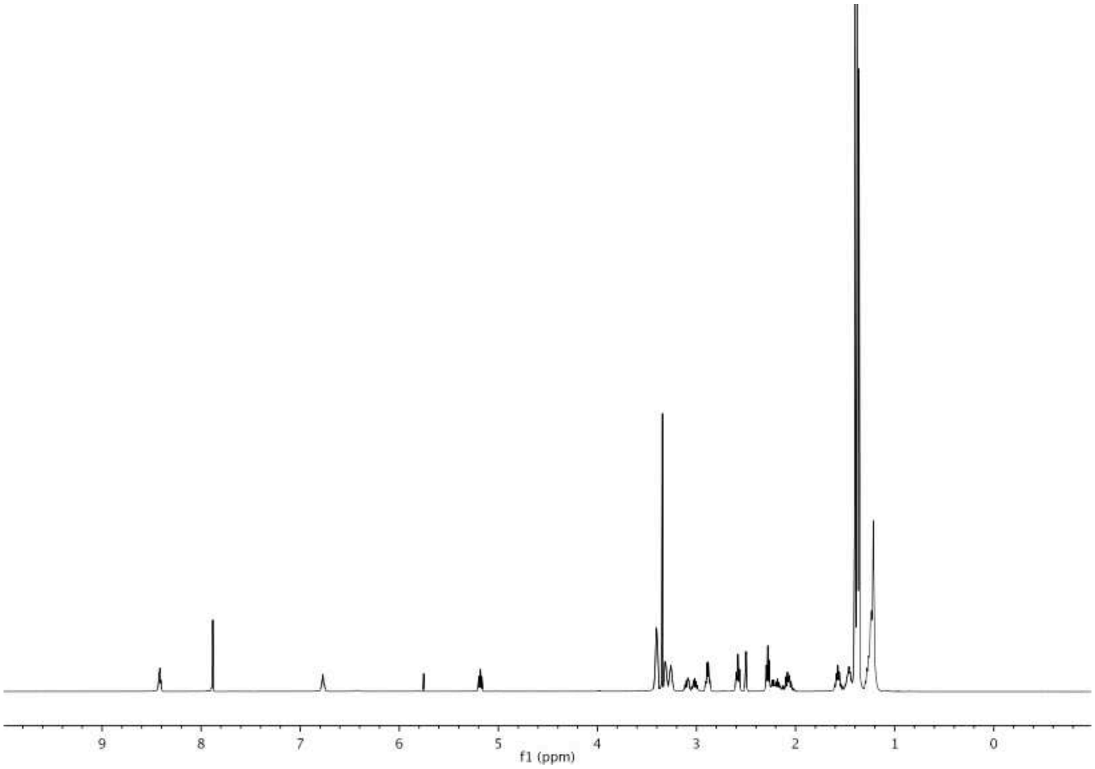

^{1}H NMR of **10_KE**

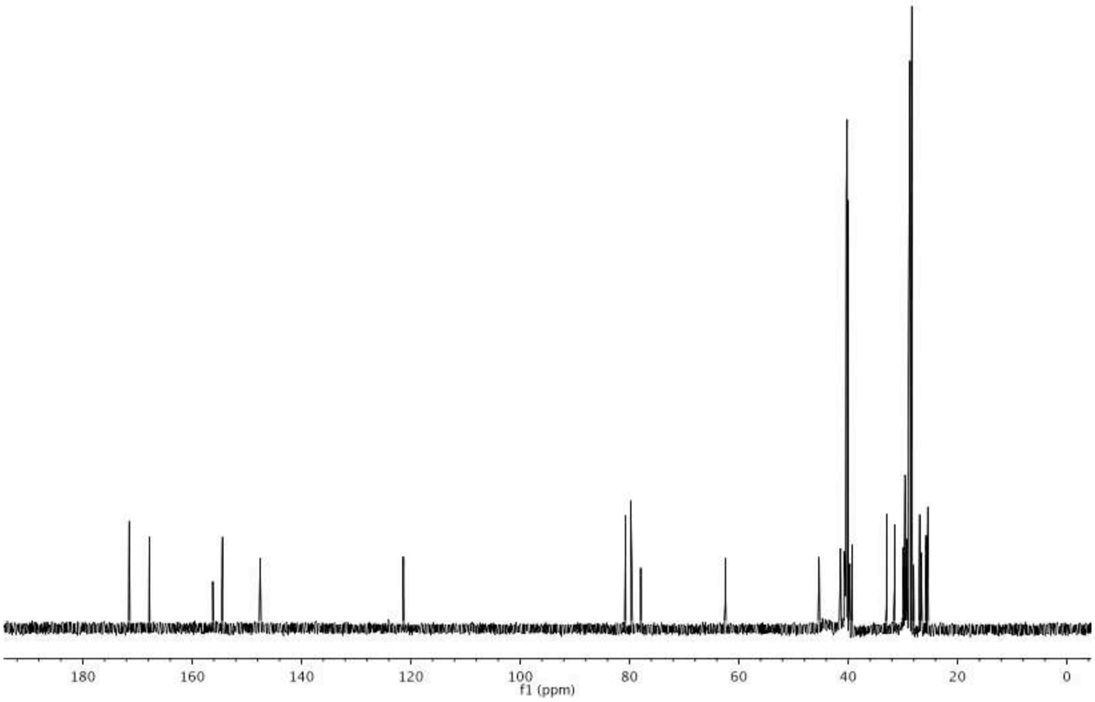

^{13}C NMR of **10_KE**

Compound 10_KG

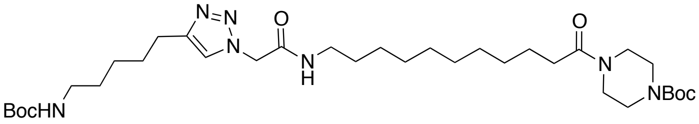

^{1}H NMR (500 MHz, DMSO-d_6) δ 8.26 (t, 1H, J = 5.5 Hz), 7.76 (s, 1H), 6.79 (t, 1H, J = 6.0 Hz), 4.98 (s, 2H), 3.41-3.25 (m, 10H), 3.08 (q, 2H, J = 7.5 Hz), 2.90 (q, 2H, J = 6.5 Hz), 2.60 (t, 2H, J = 7.5), 2.29 (t, 2H, J = 7.5), 1.40-1.36 (m, 30H), 1.24 (m, 16H)

^{13}C NMR (126 MHz, DMSO-d_6) δ 171.5, 165.9, 156.2, 154.5, 147.2, 123.9, 79.8, 77.9, 55.6, 52.2, 45.3, 41.4, 39.4, 33.0, 30.0, 29.7, 29.6(2), 29.5, 29.4(2), 28.9, 28.7, 27.0, 26.6, 25.6, 25.4

MS (MALDI, m/z) calcd for $C_{34}H_{62}N_7O_6$ $(M+H)^+$ 664.48, found 664.49

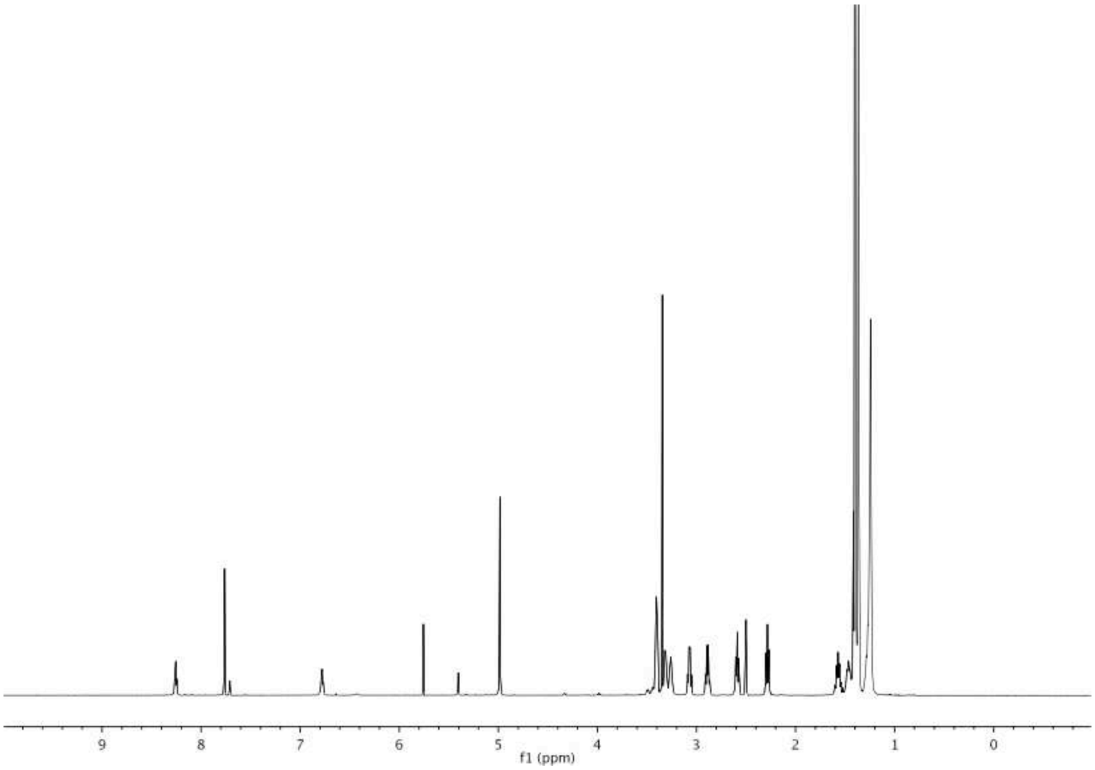

^{1}H NMR of **10_KG**

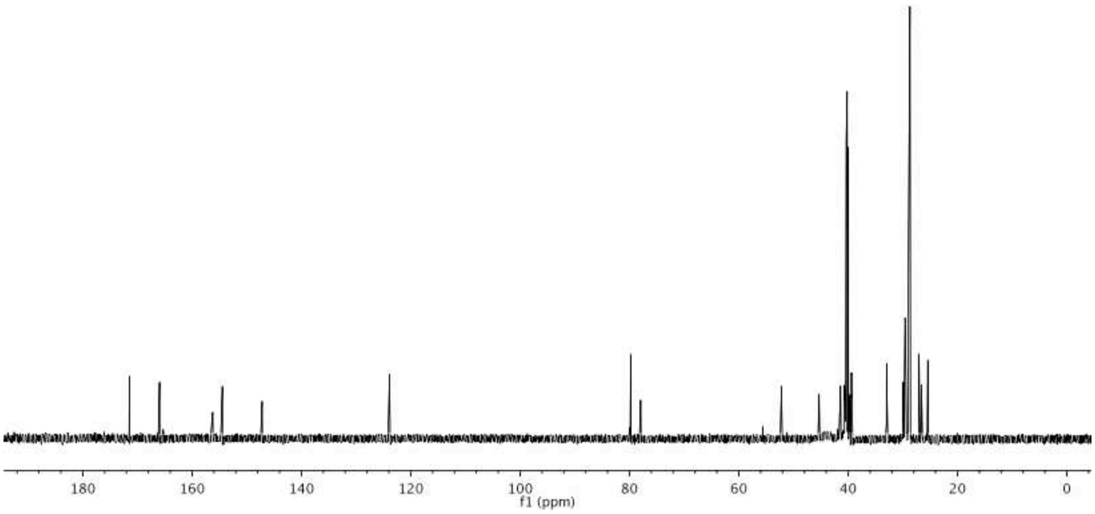

^{13}C NMR of **10_KG**

Compound 10_SM

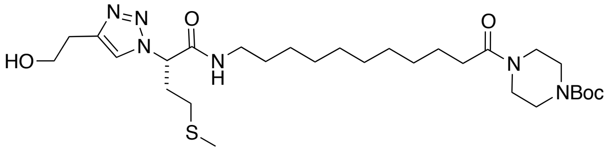

^{1}H NMR (500 MHz, DMSO-d_6) δ 8.46 (t, 1H, J = 5.5 Hz), 7.94 (s, 1H), 5.30 (m, 1H), 4.69 (t, 1H, J = 6.0 Hz), 3.64 (dd, 2H), 3.41-3.34 (m, 10H), 3.10-3.00 (m, 2H), 2.76 (t, 2H, J = 7.5 Hz), 2.29-2.26 (m, 6H), 2.03 (s, 3H), 1.40 (m, 11H), 1.22 (m, 12H)

^{13}C NMR (126 MHz, DMSO-d_6) δ 171.5, 167.9, 154.5, 145.1, 122.1, 79.8, 62.2, 61.0, 55.6, 41.4, 40.7, 39.4, 33.0, 32.3, 29.9, 29.6(3), 29.5, 29.4, 29.3, 28.7, 15.1

MS (MALDI, m/z) calcd for $C_{29}H_{53}N_6O_5S$ $(M+H)^+$ 597.38, found 597.60

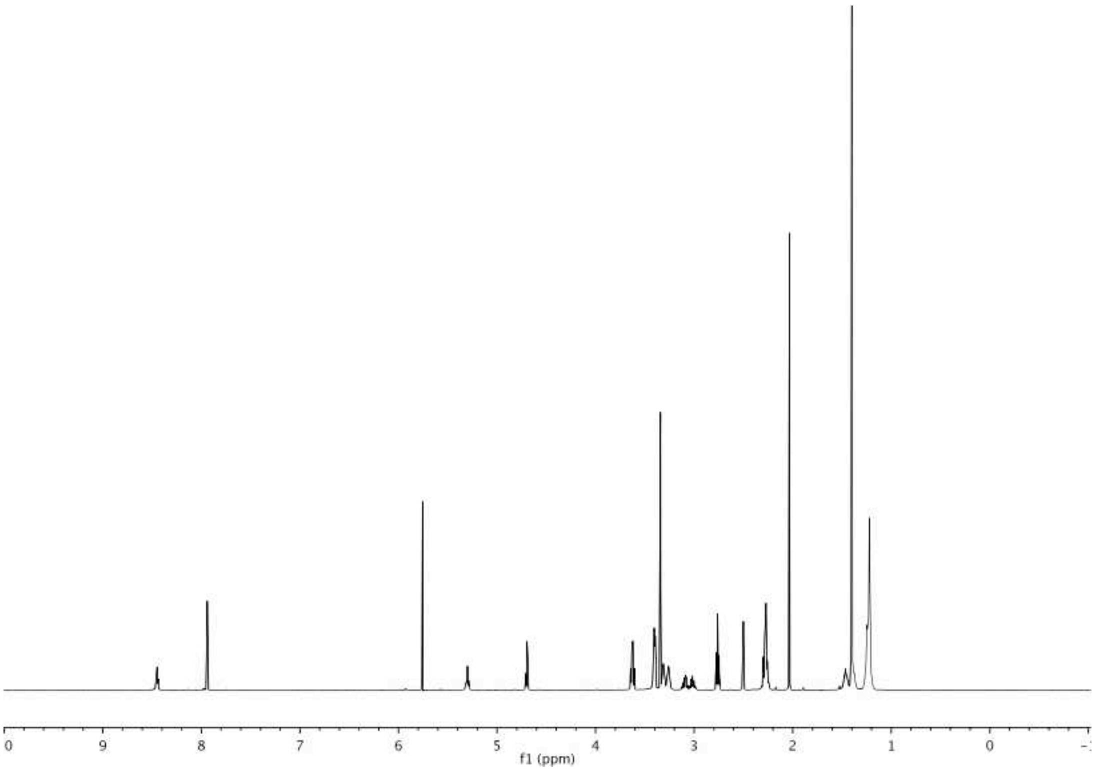

^{1}H NMR of **10_SM**

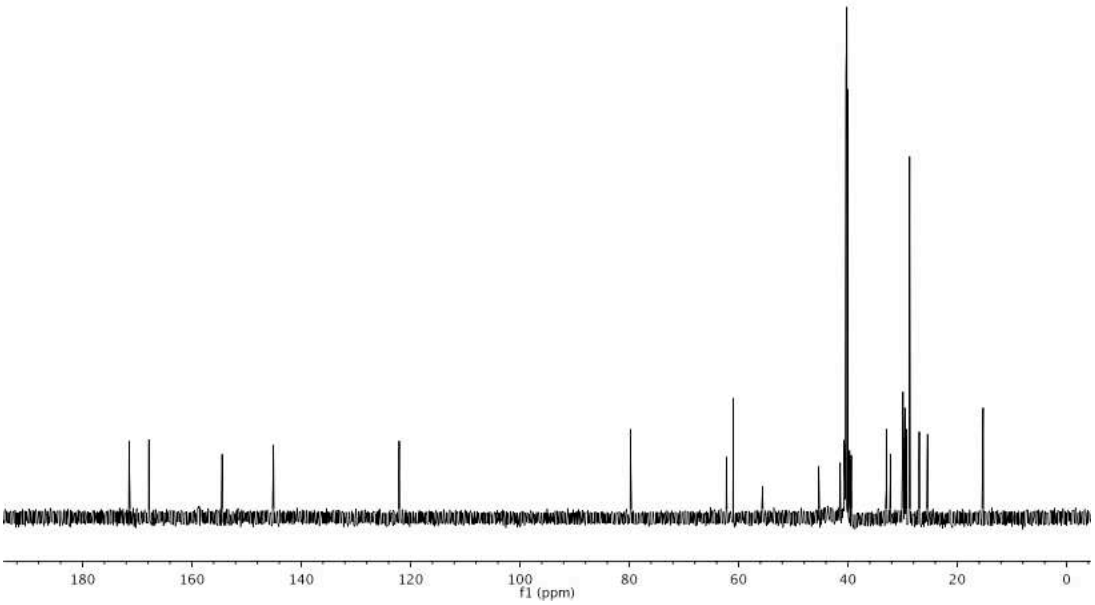

^{13}C NMR of **10_SM**

Compound 10_KS

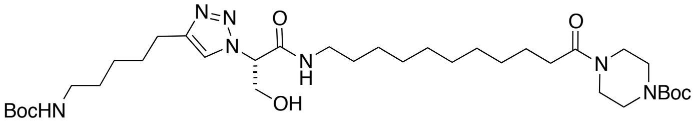

^{1}H NMR (500 MHz, DMSO-d_6) δ 8.40 (t, 1H, J = 5.5 Hz), 7.86 (s, 1H), 6.79 (t, 1H, J = 6.0 Hz), 5.28-5.22 (m, 2H), 3.92 (t, 2H, J = 6.0 Hz), 3.41-3.25 (m, 10H), 3.06 (q, 2H, J = 7.5 Hz), 2.97 (q, 2H, J = 7.5 Hz), 2.60 (t, 2H, J = 6.0 Hz), 2.29 (t, 2H, J = 6.0 Hz), 1.59 (m, 2H), 1.40 (m, 28H), 1.24 (m, 16H)

^{13}C NMR (126 MHz, DMSO-d_6) δ 171.5, 166.9, 156.2, 154.5, 147.0, 121.7, 79.8, 78.0, 65.5, 62.1, 45.3, 41.4, 39.4, 33.0, 30.0, 29.6(3), 29.5, 29.4, 29.3, 28.9, 28.7, 26.9, 26.7, 25.8, 25.4

MS (MALDI, m/z) calcd for $C_{35}H_{64}N_7O_7$ $(M+H)^+$ 694.49, found 694.51

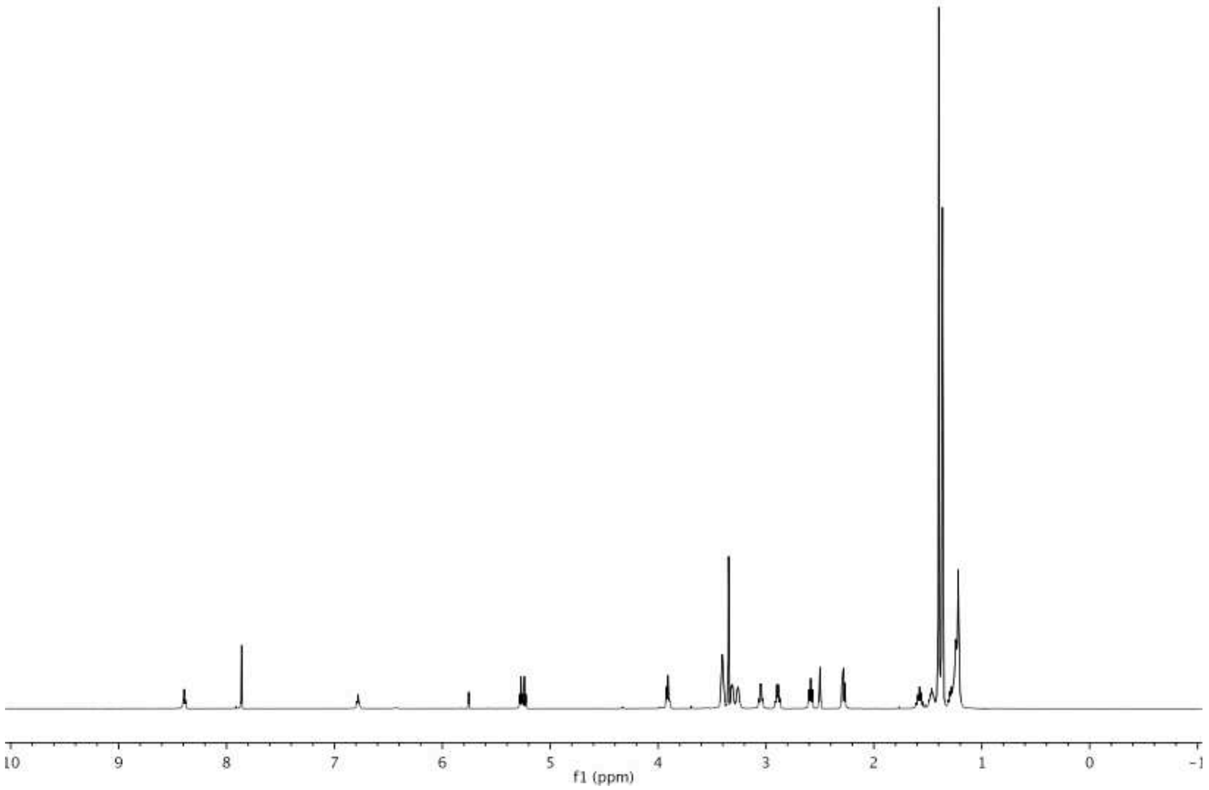

^{1}H NMR of **10_KS**

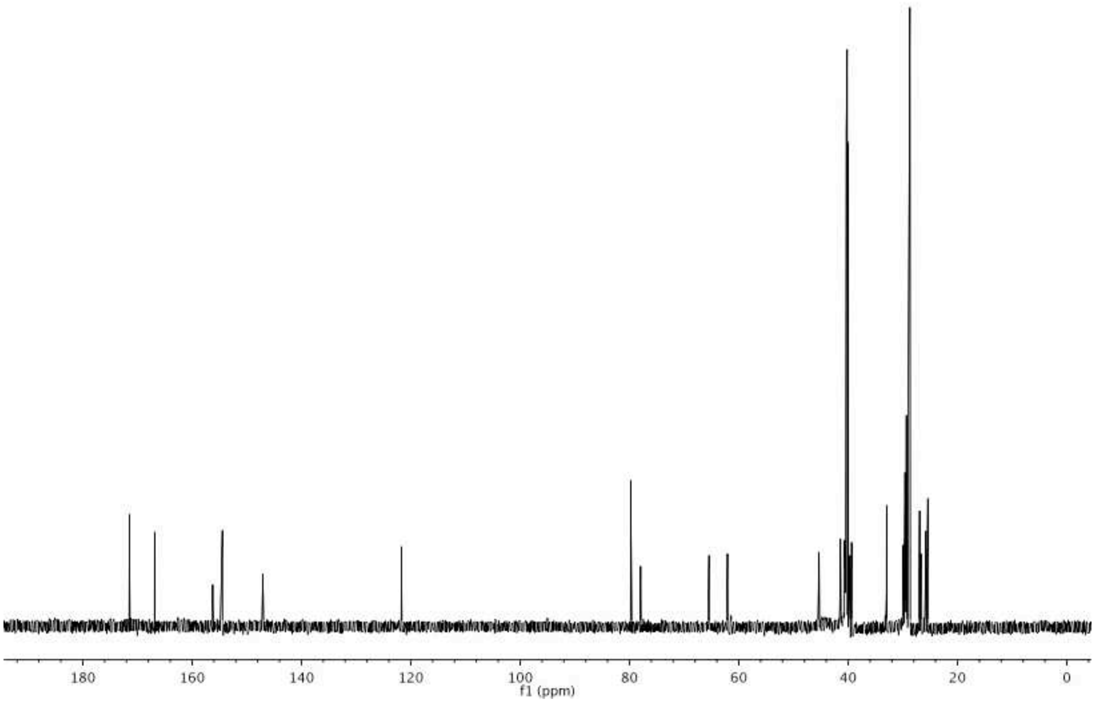

^{13}C NMR of **10_KS**

Compound 10_RI

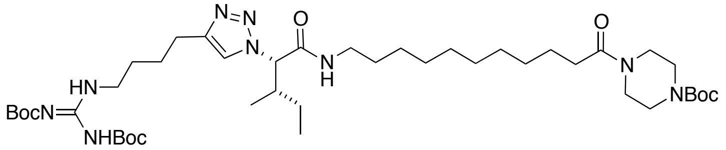

^{1}H NMR (500 MHz, DMSO-d_6) δ 11.50 (s, 1H), 8.54 (t, 1H, J = 5.5 Hz), 8.30 (t, 1H, J = 5.5 Hz), 7.90 (s, 1H), 4.96 (d, 1H, J = 11.0 Hz), 3.40-3.25 (m, 10H), 3.16 (m, 1H), 3.10 (m, 1H), 2.64 (t, 2H, J = 6.0 Hz), 2.29 (t, 2H, J = 6.0 Hz), 2.19 (m, 1H), 1.60 (m, 2H), 1.51 (m, 15H), 1.39 (m, 22H), 1.20 (m, 12H), 0.95 (d, 3H, J = 6.5 Hz), 0.75 (t, 3H, J = 7.0 Hz)

^{13}C NMR (126 MHz, DMSO-*d*$_6$) δ 171.4, 168.0, 163.8, 155.9, 154.4, 152.8, 147.4,121.1, 83.5, 79.8, 78.7, 67.8, 55.6, 45.3, 41.4, 39.2, 37.2, 33.0, 29.6(2), 29.5, 29.3(2), 28.8, 28.7(2), 28.3, 26.9, 25.4(2), 25.0, 15.7, 10.5

MS (MALDI, m/z) calcd for $C_{43}H_{78}N_9O_8$ $(M+H)^+$ 848.60, found 848.57

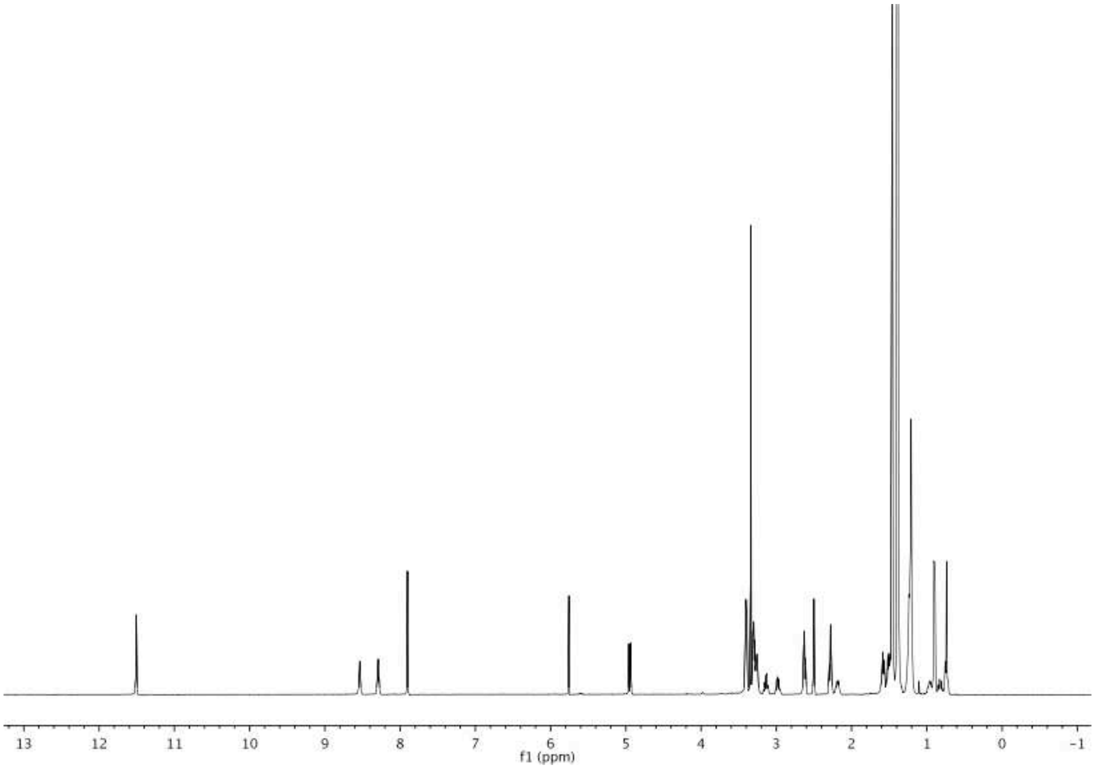

^{1}H NMR of **10_RI**

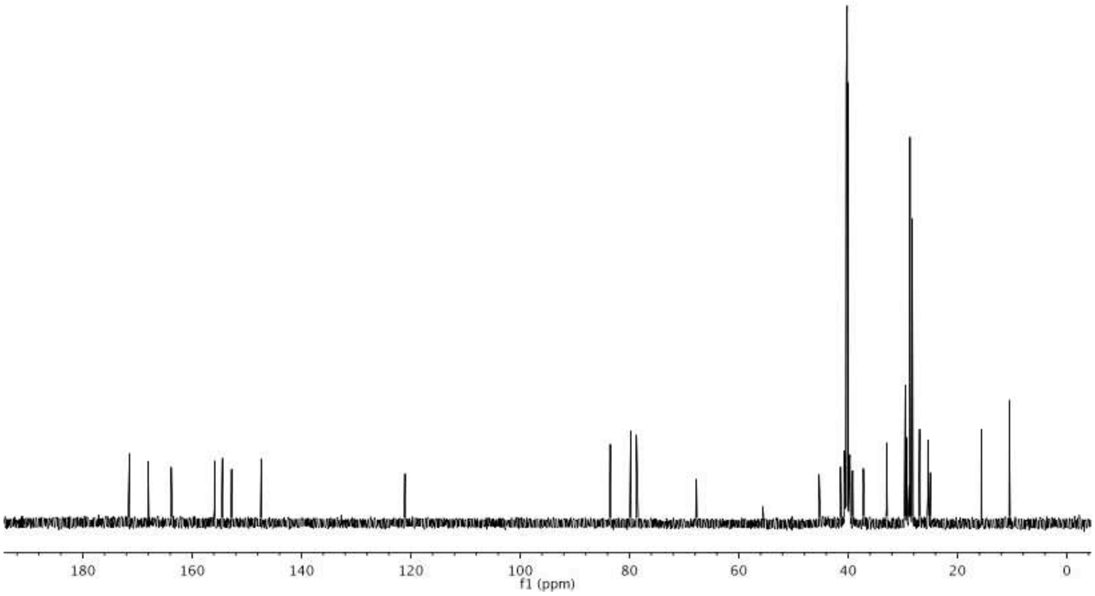

^{13}C NMR of **10_RI**

Compound 10_GT

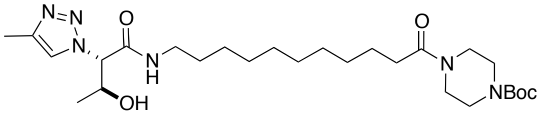

^{1}H NMR (500 MHz, DMSO-d_6) δ 8.49 (t, 1H, J = 5.5 Hz), 7.85 (s, 1H), 5.13 (d, 1H, J = 6.0 Hz), 5.012 (d, 1H, J = 6.0 Hz), 4.23 (m, 1H), 3.40-3.26 (m, 10H), 3.11-2.98 (m, 2H), 2.29 (t, 2H, J = 7.5 Hz), 2.22 (s, 3H), 1.47 (m, 14H), 1.24 (m, 12H), 1.06 (d, 3H, J = 6.0 Hz)

^{13}C NMR (126 MHz, DMSO-d_6) δ 171.5, 167.2, 154.5, 141.9, 122.3, 79.8, 69.8, 67.1, 45.3, 41.4, 39.3, 33.0, 29.6(3), 29.5, 29.3(2), 28.7, 27.0, 25.4, 20.9, 11.3

MS (MALDI, m/z) calcd for $C_{27}H_{49}N_6O_5$ $(M+H)^+$ 537.38, found 537.49

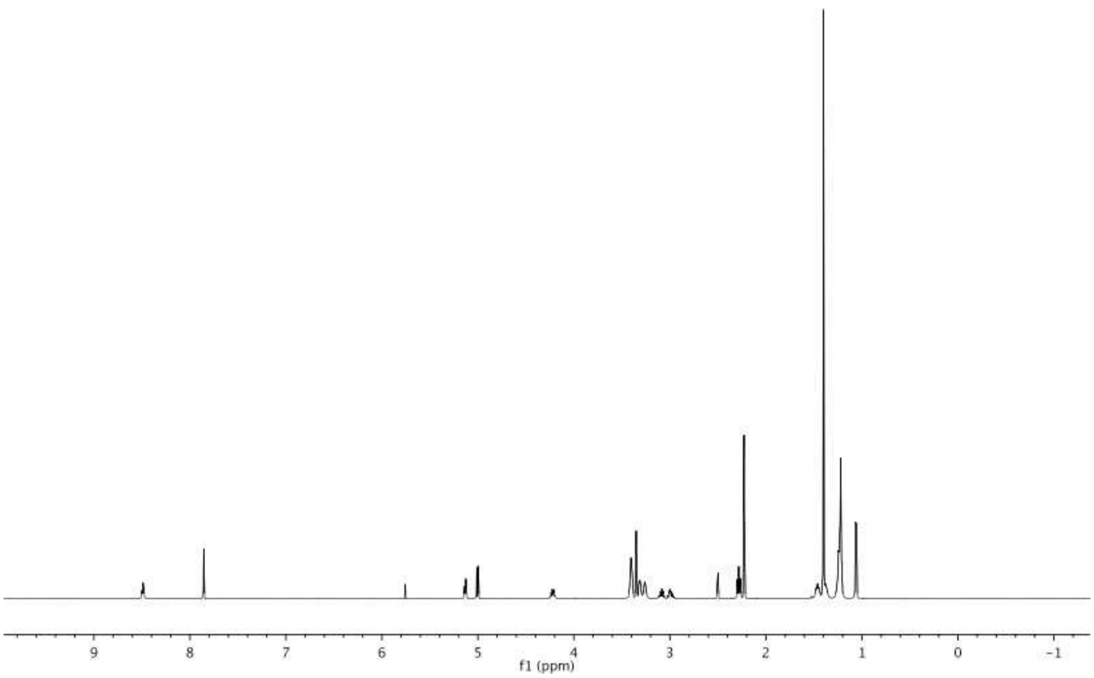

^{1}H NMR of **10_GT**

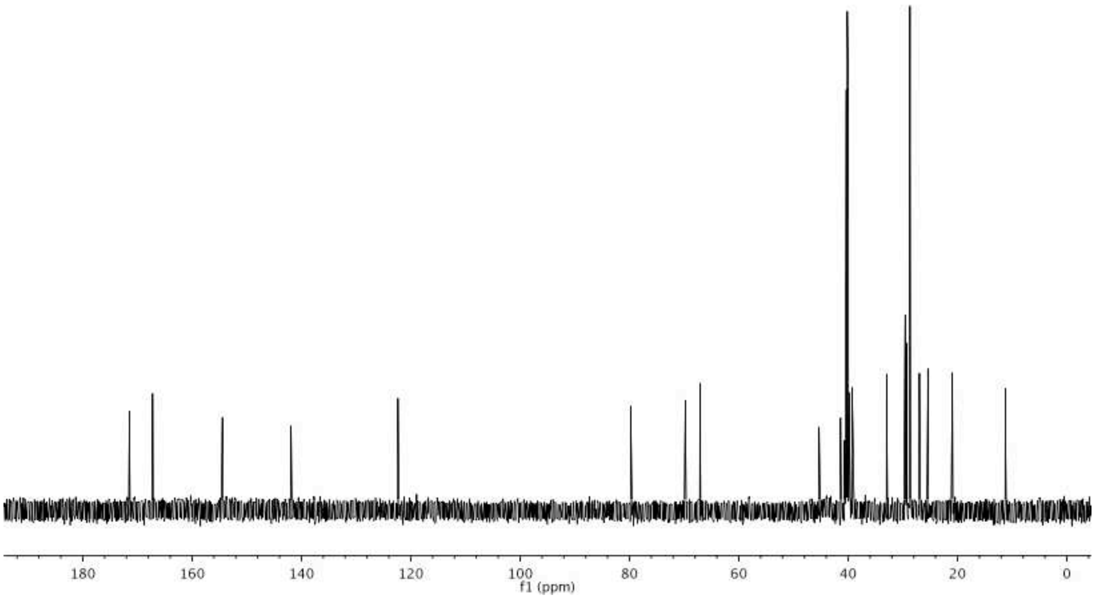

^{13}C NMR of **10_GT**

D. General Procedure and Preparation of Bivalent Mimics

General syntheses for bivalent mimics were modified from the published papers.[6,8] Compounds **10** (1.0 eq.) were treated with 50% TFA in CH_2Cl_2 for 12h at 25 ºC and then the solvent was removed. The resulting residue was dissolved in THF (0.04 M), and then tag **11** (1.0 eq) and K_2CO_3 (4.0 eq.) were added. The suspension was stirred for 18 h at 25 ºC and then the solvent was removed. Crude product **12** was used for the next step without further purification. The resulting crude product **12** was dissolved in DMSO (0.04 M), and then another deprotected compound **10'** and K_2CO_3 (4.0 eq.) were added. The suspension was stirred for 10 – 14 days at 25 or 50 °C and the reaction was monitored by analytical HPLC. After the starting material was consumed, the mixture was lyophilized to remove DMSO. The materials were re-dissolved in 1:1 mixture of H_2O/CH_3CN, and then purified by preparative HPLC to yield the final products **1**.

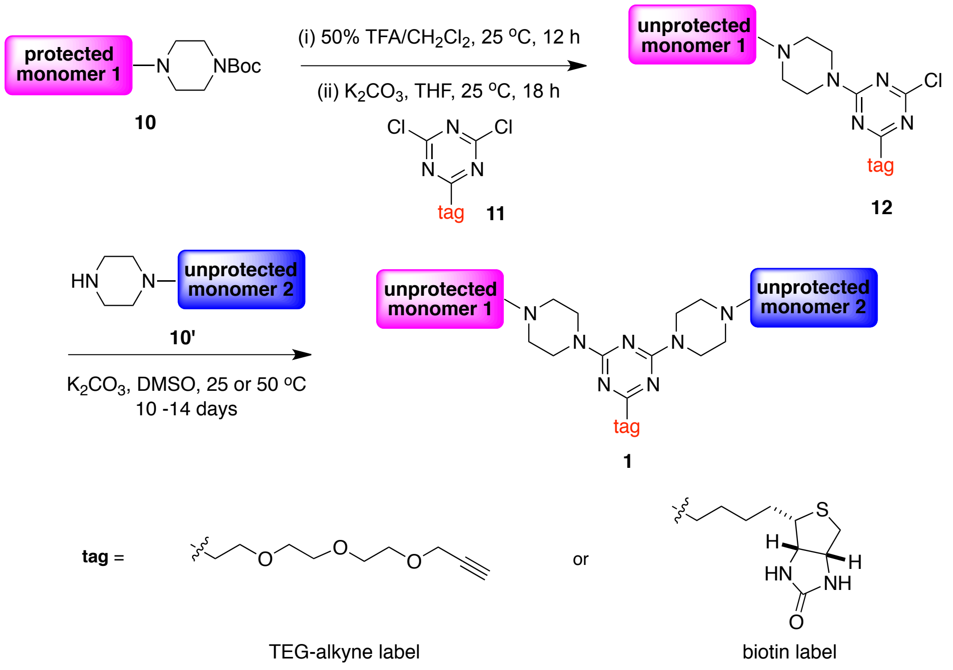

E. Table S2. Characterization of Compounds with TEG-alkyne Label.

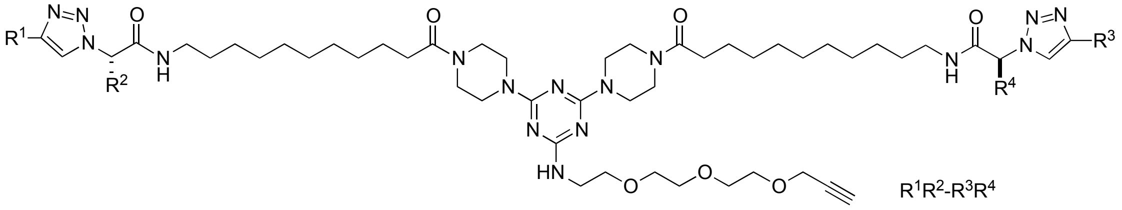

R^1R^2-R^3R^4

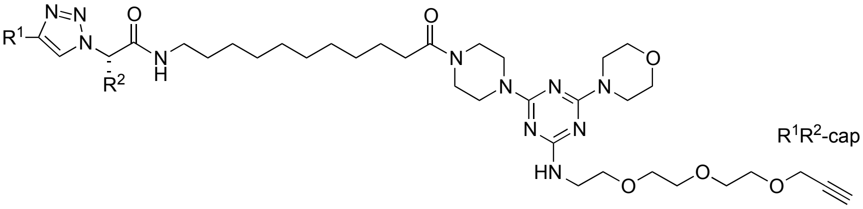

R^1R^2-cap

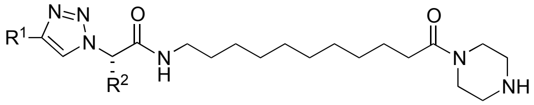

R^1R^2

	compound code	sequence (R^1R^2-R^3R^4)	SEDEX purity (%)	retention time (min)	chemical formula	$[M+H]^+$ calculated	$[M+H]^+$ found
**1**	**KB1365**	KI-KI	100	19.0	$C_{68}H_{121}N_{18}O_7$	1302.0	1302.0
**2**	**KB1366**	KI-KG	100	17.0	$C_{64}H_{113}N_{18}O_7$	1245.7	1245.9
**3**	**KB1367**	KI-KE	100	17.2	$C_{67}H_{117}N_{18}O_9$	1317.9	1317.9
**4**	**KB1368**	KI-KS	100	16.6	$C_{65}H_{115}N_{18}O_8$	1275.9	1275.8
**5**	**KB1369**	KI-GT	100	16.7	$C_{62}H_{108}N_{17}O_8$	1218.9	1218.9
**6**	**KB1370**	KI-RI	100	19.6	$C_{68}H_{121}N_{20}O_7$	1330.0	1330.0
**7**	**KB1371**	KI-SM	100	17.0	$C_{64}H_{112}N_{17}O_8S$	1278.9	1278.9
**8**	**KB1372**	KG-KG	100	15.6	$C_{60}H_{105}N_{18}O_7$	1189.8	1189.8
**9**	**KB1373**	KG-KE	100	14.4	$C_{63}H_{109}N_{18}O_9$	1261.9	1262.0
**10**	**KB1374**	KG-KS	100	13.2	$C_{61}H_{107}N_{18}O_8$	1219.9	1219.8
**11**	**KB1375**	KG-GT	100	14.8	$C_{58}H_{100}N_{17}O_8$	1162.8	1162.7
**12**	**KB1376**	KG-RI	100	17.6	$C_{64}H_{113}N_{20}O_7$	1273.9	1273.8
**13**	**KB1377**	KG-SM	100	16.9	$C_{60}H_{104}N_{17}O_8S$	1222.8	1222.8
**14**	**KB1378**	KE-KE	100	15.0	$C_{66}H_{113}N_{18}O_{11}$	1333.9	1333.9
**15**	**KB1379**	KE-KS	91	14.9	$C_{64}H_{111}N_{18}O_{10}$	1291.9	1291.9
**16**	**KB1380**	KE-GT	100	16.7	$C_{61}H_{104}N_{17}O_{10}$	1234.8	1234.7
**17**	**KB1381**	KE-RI	100	17.1	$C_{67}H_{117}N_{20}O_9$	1345.9	1345.8
**18**	**KB1382**	KE-SM	100	16.7	$C_{63}H_{108}N_{17}O_{10}S$	1294.8	1294.8

**19**	**KB1383**	KS-KS	100	14.7	$C_{62}H_{109}N_{18}O_{9}$	1249.9	1249.9
**20**	**KB1384**	KS-GT	100	14.4	$C_{59}H_{102}N_{17}O_{9}$	1192.8	1192.8
**21**	**KB1385**	KS-RI	100	17.3	$C_{65}H_{115}N_{20}O_{8}$	1303.9	1303.9
**22**	**KB1386**	KS-SM	100	17.0	$C_{61}H_{106}N_{17}O_{9}S$	1252.8	1252.8
**23**	**KB1387**	GT-GT	100	15.3	$C_{56}H_{95}N_{16}O_{9}$	1135.7	1135.6
**24**	**KB1388**	GT-RI	100	18.8	$C_{62}H_{108}N_{19}O_{8}$	1246.9	1246.9
**25**	**KB1389**	GT-SM	98	18.1	$C_{58}H_{99}N_{16}O_{9}S$	1195.8	1195.6
**26**	**KB1390**	RI-RI	100	18.9	$C_{68}H_{121}N_{22}O_{7}$	1358.0	1357.9
**27**	**KB1391**	RI-SM	100	19.7	$C_{64}H_{112}N_{19}O_{8}S$	1306.9	1306.9
**28**	**KB1392**	SM-SM	100	18.8	$C_{60}H_{103}N_{16}O_{9}S_{2}$	1255.8	1255.7
**29**	**KB1393**	SM	100	15.7	$C_{24}H_{45}N_{6}O_{3}S$	497.3	497.1
**30**	**KB1394**	RI	100	11.3	$C_{28}H_{54}N_{9}O_{2}$	548.4	548.3
**31**	**KB1395**	KI	100	11.5	$C_{28}H_{54}N_{7}O_{2}$	520.4	520.2
**32**	**KB1396**	KG	100	10.0	$C_{24}H_{46}N_{7}O_{2}$	464.4	464.1
**33**	**KB1397**	KE	100	10.1	$C_{27}H_{50}N_{7}O_{4}$	536.4	536.2
**34**	**KB1398**	KS	100	8.6	$C_{25}H_{48}N_{7}O_{3}$	494.4	494.1
**35**	**KB1399**	GT	100	9.7	$C_{22}H_{41}N_{6}O_{3}$	437.3	437.1
**36**	**KB1445**	TG-TG	98	14.5	$C_{56}H_{95}N_{16}O_{9}$	1135.8	1135.5
**37**	**KB1446**	TG-MS	100	15.8	$C_{58}H_{99}N_{16}O_{9}S$	1195.8	1195.6
**38**	**KB1447**	TG-EK	96	13.8	$C_{61}H_{104}N_{17}O_{10}$	1234.8	1234.6
**39**	**KB1448**	TG-GK	100	14.2	$C_{58}H_{100}N_{17}O_{8}$	1162.8	1162.5
**40**	**KB1449**	TG-SK	100	14.4	$C_{59}H_{102}N_{17}O_{9}$	1192.8	1192.8
**41**	**KB1450**	TG-IK	100	16.3	$C_{62}H_{108}N_{17}O_{8}$	1218.9	1218.6
**42**	**KB1451**	TG-IR	94	17.1	$C_{62}H_{108}N_{19}O_{8}$	1246.9	1246.7
**43**	**KB1452**	MS-MS	90	17.2	$C_{60}H_{103}N_{16}O_{9}S_{2}$	1255.8	1255.8
**44**	**KB1453**	MS-EK	93	14.9	$C_{63}H_{108}N_{17}O_{10}S$	1294.8	1294.8
**45**	**KB1454**	MS-GK	100	16.8	$C_{60}H_{104}N_{17}O_{8}S$	1222.8	1222.6
**46**	**KB1455**	MS-SK	100	14.9	$C_{61}H_{106}N_{17}O_{9}S$	1252.8	1252.7
**47**	**KB1456**	MS-IK	92	17.7	$C_{64}H_{112}N_{17}O_{8}S$	1278.9	1278.7
**48**	**KB1457**	MS-IR	100	17.8	$C_{64}H_{112}N_{19}O_{8}S$	1306.9	1306.7
**49**	**KB1458**	EK-EK	100	12.9	$C_{66}H_{113}N_{18}O_{11}$	1333.9	1333.8
**50**	**KB1459**	EK-GK	97	13.0	$C_{63}H_{109}N_{18}O_{9}$	1261.9	1261.9
**51**	**KB1460**	EK-SK	100	12.5	$C_{64}H_{111}N_{18}O_{10}$	1291.9	1292.0
**52**	**KB1461**	EK-IK	84	15.7	$C_{67}H_{117}N_{18}O_{9}$	1317.9	1318.0
**53**	**KB1462**	EK-IR	98	17.0	$C_{67}H_{117}N_{20}O_{9}$	1345.9	1346.0
**54**	**KB1463**	GK-GK	100	13.4	$C_{60}H_{105}N_{18}O_{7}$	1189.8	1190.1

**55**	**KB1464**	GK-SK	95	13.5	$C_{61}H_{106}N_{18}O_8$	1218.8	1218.7
**56**	**KB1465**	GK-IK	99	15.4	$C_{64}H_{113}N_{18}O_7$	1245.9	1245.7
**57**	**KB1466**	GK-IR	96	15.6	$C_{64}H_{113}N_{20}O_7$	1273.9	1273.8
**58**	**KB1467**	SK-SK	100	12.9	$C_{62}H_{109}N_{18}O_9$	1249.9	1249.7
**59**	**KB1468**	SK-IK	100	14.8	$C_{65}H_{115}N_{18}O_8$	1275.9	1275.7
**60**	**KB1469**	SK-IR	89	15.4	$C_{65}H_{115}N_{20}O_8$	1303.9	1303.7
**61**	**KB1470**	IK-IK	100	16.5	$C_{68}H_{121}N_{18}O_7$	1302.0	1302.0
**62**	**KB1471**	IK-IR	100	17.8	$C_{68}H_{121}N_{20}O_7$	1330.0	1329.8
**63**	**KB1472**	IR-IR	100	17.6	$C_{68}H_{121}N_{22}O_7$	1358.0	1357.7
**64**	**KB1473**	TG	100	8.9	$C_{22}H_{41}N_6O_3$	437.3	437.1
**65**	**KB1474**	MS	97	11.1	$C_{24}H_{45}N_6O_3S$	497.3	497.2
**66**	**KB1475**	EK	100	8.6	$C_{27}H_{50}N_7O_4$	536.4	536.3
**67**	**KB1476**	GK	100	8.8	$C_{24}H_{46}N_7O_2$	464.4	464.2
**68**	**KB1477**	SK	94	8.0	$C_{25}H_{48}N_7O_3$	494.4	494.2
**69**	**KB1478**	IK	94	11.4	$C_{28}H_{54}N_7O_2$	520.8	520.3
**70**	**KB1479**	IR	100	11.4	$C_{28}H_{54}N_9O_2$	548.4	548.4
**71**	**KB1480**	TG-CAP	100	13.6	$C_{38}H_{64}N_{11}O_7$	786.5	786.3
**72**	**KB1481**	MS-CAP	100	15.8	$C_{40}H_{68}N_{11}O_7S$	846.5	845.3
**73**	**KB1482**	EK-CAP	100	10.9	$C_{43}H_{73}N_{12}O_8$	885.6	885.2
**74**	**KB1483**	GK-CAP	100	14.2	$C_{40}H_{69}N_{12}O_6$	813.5	813.3
**75**	**KB1484**	SK-CAP	99	12.3	$C_{41}H_{71}N_{12}O_7$	843.6	843.3
**76**	**KB1485**	IK-CAP	100	16.1	$C_{44}H_{77}N_{12}O_6$	869.6	869.4
**77**	**KB1486**	IR-CAP	100	18.0	$C_{44}H_{77}N_{14}O_6$	897.6	897.4
**78**	**KB1551**	KI-IK	100	17.5	$C_{68}H_{121}N_{18}O_7$	1302.0	1301.8
**79**	**KB1552**	KI-GK	100	15.6	$C_{64}H_{112}N_{18}NaO_7$	1267.9	1267.8
**80**	**KB1553**	KI-EK	100	16.9	$C_{67}H_{117}N_{18}O_9$	1317.9	1318.1
**81**	**KB1554**	KI-SK	100	15.1	$C_{65}H_{115}N_{18}O_8$	1275.9	1275.6
**82**	**KB1555**	KI-TG	100	16.9	$C_{62}H_{108}N_{17}O_8$	1218.9	1219.0
**83**	**KB1556**	KI-MS	100	18.7	$C_{64}H_{112}N_{17}O_8S$	1278.9	1278.9
**84**	**KB1557**	KG-IK	100	17.0	$C_{64}H_{113}N_{18}O_7$	1245.9	1246.0
**85**	**KB1558**	KG-GK	100	14.7	$C_{60}H_{104}N_{18}NaO_7$	1211.8	1211.8
**86**	**KB1559**	KG-EK	100	14.6	$C_{63}H_{109}N_{18}O_9$	1261.9	1261.9
**87**	**KB1560**	KG-SK	100	14.1	$C_{61}H_{107}N_{18}O_8$	1219.9	1219.8
**88**	**KB1561**	KG-TG	100	14.1	$C_{58}H_{100}N_{17}O_8$	1162.8	1162.9
**89**	**KB1562**	KG-IR	100	18.0	$C_{64}H_{113}N_{20}O_7$	1273.9	1274.1
**90**	**KB1563**	KG-MS	100	16.6	$C_{60}H_{104}N_{17}O_8S$	1222.8	1222.6

**91**	**KB1564**	KE-IK	100	16.4	$C_{67}H_{117}N_{18}O_9$	1317.9	1318.1
**92**	**KB1565**	KE-GK	99	13.5	$C_{63}H_{109}N_{18}O_9$	1261.9	1261.8
**93**	**KB1566**	KE-EK	100	15.0	$C_{66}H_{113}N_{18}O_{11}$	1333.9	1333.9
**94**	**KB1567**	KE-SK	100	13.2	$C_{64}H_{110}N_{18}NaO_{10}$	1313.9	1313.9
**95**	**KB1568**	KE-TG	87	14.3	$C_{61}H_{104}N_{17}O_{10}$	1234.8	1234.9
**96**	**KB1569**	KE-IR	99	16.7	$C_{67}H_{117}N_{20}O_9$	1345.9	1346.0
**97**	**KB1570**	KE-MS	100	15.4	$C_{63}H_{108}N_{17}O_{10}S$	1294.8	1294.6
**98**	**KB1571**	KS-IK	100	16.0	$C_{65}H_{115}N_{18}O_8$	1275.9	1275.8
**99**	**KB1572**	KS-GK	97	13.6	$C_{61}H_{107}N_{18}O_8$	1219.9	1219.9
**100**	**KB1573**	KS-EK	100	14.9	$C_{64}H_{111}N_{18}O_{10}$	1291.9	1291.9
**101**	**KB1574**	KS-SK	100	14.9	$C_{62}H_{109}N_{18}O_9$	1249.9	1249.8
**102**	**KB1575**	KS-TG	100	14.6	$C_{59}H_{102}N_{17}O_9$	1192.8	1192.7429
**103**	**KB1576**	KS-MS	99	15.4	$C_{61}H_{106}N_{17}O_9S$	1252.8	1252.5
**104**	**KB1577**	GT-IK	100	17.6	$C_{62}H_{108}N_{17}O_8$	1218.9	1218.7
**105**	**KB1578**	GT-GK	100	14.4	$C_{58}H_{100}N_{17}O_8$	1162.8	1162.9
**106**	**KB1579**	GT-EK	100	16.2	$C_{61}H_{104}N_{17}O_{10}$	1234.8	1234.8
**107**	**KB1580**	GT-SK	100	14.4	$C_{59}H_{102}N_{17}O_9$	1192.8	1192.9
**108**	**KB1581**	GT-TG	100	14.9	$C_{56}H_{95}N_{16}O_9$	1135.7	1135.9
**109**	**KB1582**	GT-IR	100	17.1	$C_{62}H_{108}N_{19}O_8$	1246.9	1247.0
**110**	**KB1583**	GT-MS	100	16.4	$C_{58}H_{99}N_{16}O_9S$	1195.8	1195.7
**111**	**KB1584**	RI-IK	100	19.3	$C_{68}H_{121}N_{20}O_7$	1330.0	1330.0
**112**	**KB1585**	RI-GK	100	16.5	$C_{64}H_{113}N_{20}O_7$	1273.9	1273.9
**113**	**KB1586**	RI-EK	100	17.3	$C_{67}H_{117}N_{20}O_9$	1345.9	1346.1
**114**	**KB1587**	RI-SK	100	15.7	$C_{65}H_{115}N_{20}O_8$	1303.9	1304.1
**115**	**KB1588**	RI-TG	100	17.2	$C_{62}H_{108}N_{19}O_8$	1246.9	1246.6
**116**	**KB1589**	RI-MS	100	18.2	$C_{64}H_{112}N_{19}O_8S$	1306.9	1306.6
**117**	**KB1590**	SM-IK	100	17.8	$C_{64}H_{112}N_{17}O_8S$	1278.9	1278.7
**118**	**KB1591**	SM-GK	100	15.7	$C_{60}H_{104}N_{17}O_8S$	1222.8	1222.8
**119**	**KB1592**	SM-EK	100	14.6	$C_{63}H_{108}N_{17}O_{10}S$	1294.8	1294.9
**120**	**KB1593**	SM-SK	100	15.0	$C_{61}H_{106}N_{17}O_9S$	1252.8	1252.6
**121**	**KB1594**	SM-TG	100	15.6	$C_{58}H_{99}N_{16}O_9S$	1195.8	1195.6
**122**	**KB1595**	SM-IR	100	18.3	$C_{64}H_{112}N_{19}O_8S$	1306.9	1306.8
**123**	**KB1596**	SM-MS	96	15.5	$C_{60}H_{103}N_{16}O_9S_2$	1255.8	1255.6
**124**	**KB1597**	KI-Cap	100	16.1	$C_{44}H_{77}N_{12}O_6$	869.6	869.6
**125**	**KB1598**	KG-Cap	100	13.5	$C_{40}H_{69}N_{12}O_6$	813.5	813.4
**126**	**KB1599**	KE-Cap	94	14.6	$C_{43}H_{73}N_{12}O_8$	885.6	885.5

**127**	**KB1600**	KS-Cap	100	13.0	$C_{41}H_{71}N_{12}O_7$	843.6	843.5
**128**	**KB1601**	GT-Cap	100	16.6	$C_{38}H_{64}N_{11}O_7$	786.5	786.5
**129**	**KB1602**	RI-Cap	100	15.9	$C_{44}H_{77}N_{14}O_6$	897.6	897.6
**130**	**KB1603**	SM-Cap	100	14.8	$C_{40}H_{68}N_{11}O_7S$	846.5	846.3
**131**	**KB1604**	KI-IR	100	18.9	$C_{68}H_{121}N_{20}O_7$	1330.0	1329.9
**132**	**KB1605**	KS-IR	100	15.3	$C_{65}H_{115}N_{20}O_8$	1303.9	1303.9
**133**	**KB1606**	RI-IR	100	19.6	$C_{68}H_{121}N_{22}O_7$	1358.0	1357.7

F. Table S3. Characterization of Compounds with Biotin.

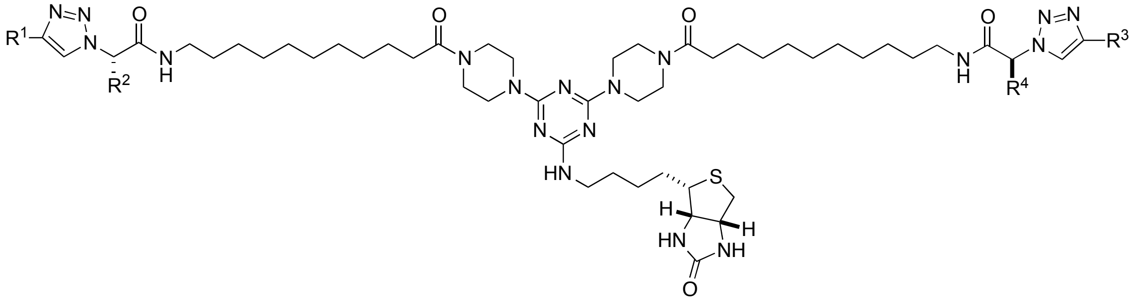

	compound code	TEG code	sequence (R^1R^2-R^3R^4)	SEDEX purity (%)	retention time (min)	chemical formula	$[M+H]^+$ calculated	$[M+H]^+$ found
**1**	KB1923	KB1468	SK-IK	100	13.2	$C_{65}H_{115}N_{20}O_6S$	1303.9	1303.6
**2**	KB1924	KB1471	IK-IR	100	14.8	$C_{68}H_{121}N_{22}O_5S$	1358.0	1358.0
**3**	KB1925	KB1579	GT-EK	100	13.2	$C_{61}H_{104}N_{19}O_8S$	1262.8	1262.8
**4**	KB1926	KB1588	RI-TG	100	14.2	$C_{62}H_{108}N_{21}O_6S$	1274.9	1274.8
**5**	KB1927	KB1591	SM-GK	100	13.8	$C_{60}H_{104}N_{19}O_6S_2$	1250.7	1250.7
**6**	KB1811	KB1368	KI-KS	100	13.1	$C_{65}H_{116}N_{20}O_6S$	1304.9	1304.8

G. References

(1) Pattarawarapan, M.; Burgess, K. *J. Med. Chem.* **2003**, *46*, 5277-5291.

(2) Chaume, G.; Kuligowski, C.; Bezzenine-Laffolee, S.; Ricard, L.; Pancrazi, A.; Ardisson, J. *Synthesis* **2004**, 3029-3036.

(3) Aronica, Laura A.; Terreni, S.; Caporusso, Anna M.; Salvadori, P. *Eur. J. Org. Chem.* **2001**, *2001*, 4321-4329.

(4) Tojino, M.; Uenoyama, Y.; Fukuyama, T.; Ryu, I. *Chemical Communications* **2004**, *0*, 2482-2483.

(5) Hideto Ito, T. H., Hirohisa Ohmiya and Masaya Sawamura *Beilstein J. Org. Chem.* **2011**, *7*, 951-959.

(6) Angell, Y.; Chen, D.; Brahimi, F.; Saragovi, H. U.; Burgess, K. *J. Am. Chem. Soc.* **2008**, *130*, 556-565.

(7) Capella, L.; Montevecchi, P. C.; Nanni, D. *J. Org. Chem.* **1994**, *59*, 3368-3374.

(8) Chen, D.; Brahimi, F.; Angell, Y.; Li, Y.-C.; Moscowicz, J.; Saragovi, H. U.; Burgess, K. *ACS Chem. Biol.* **2009**, *4*, 769-781
